# Supplementary material for: Selective inhibition reveals the regulatory function of DYRK2 in protein synthesis and calcium entry
Source: eLife. 2022 Apr 19;11:e77696. doi: 10.7554/eLife.77696 (PMC9113749; doi:10.7554/eLife.77696)
Supplement: Figure 1—source data 1. [file elife-77696-fig1-data1.zip › Figure 1-source data 1.docx]

|  | DYRK2-C5 | DYRK2-C6 | DYRK2-C7 | DYRK2-C8 | DYRK2-C10 |
| --- | --- | --- | --- | --- | --- |
| PDB code | 7DH3 | 7DG4 | 7DH9 | 7DHV | 7DHC |
| **Data collection** |  |  |  |  |  |
| Space group | C 2 2 21 | C 2 2 21 | C 2 2 21 | C 2 2 21 | C 2 2 21 |
| Cell dimensions (Å)  α, β, γ (°) | 64.56 128.83 132.45  90, 90, 90 | 64.58 128.88 132.48  90, 90, 90 | 64.142 128.44 134.106  90, 90, 90 | 64.546 128.782 132.735  90, 90, 90 | 64.427 128.382 132.446  90, 90, 90 |
| Wavelength (Å) | 1.0000 | 1.0000 | 1.0000 | 1.0000 | 1.0000 |
| Resolution (Å)  CC1/2  Rpim | 50.00-2.33(2.41-2.33)  0.999(0.807)  0.028(0.311) | 50.00-2.58 (2.67-2.58)  0.99(0.818)  0.073(0.389) | 50.00-2.19 (2.27-2.19)  0.998(0.821)  0.037(0.296) | 50.00-2.68(2.78-2.68)  0.985(0.689)  0.054(0.420) | 50.00-2.59(2.69-2.59)  0.956(0.786)  0.066(0.448) |
| *R*_merge_ | 0.093(1.006) | 0.183(0.945) | 0.127(1.001) | 0.186(1.257) | 0.138(0.993) |
| *I* / σ*I* | 15.9(2.2) | 7.9(2.3) | 24.4(3.0) | 18.3(2.3) | 11.6(2.4) |
| Completeness (%) | 96.4(99.8) | 99.9 (99.8) | 100.0 (100.0) | 100.0 (100.0) | 99.4 (99.8) |
| redundancy | 12.9(13.5) | 7.7(7.3) | 12.7(12.3) | 12.7(9.7) | 5.4(5.6) |
| Wilson B-factor | 51.65 | 62.02 | 32.11 | 49.20 | 40.65 |
| **Refinement** |  |  |  |  |  |
| No. reflections | 23126 | 17733 | 28701 | 14996 | 16633 |
| *R*_work_ / *R*_free_ | 0.193 / 0.217 | 0.201 / 0.248 | 0.186 / 0.219 | 0.190 / 0.250 | 0.179 / 0.223 |
| No. of atoms |  |  |  |  |  |
| Protein | 2646 | 2646 | 2646 | 2646 | 2646 |
| Ligand/ion | 26 | 26 | 28 | 29 | 29 |
| Water | 65 | 23 | 215 | 34 | 38 |
| *B*-factors |  |  |  |  |  |
| Macromolecules | 56.73 | 66.21 | 37.25 | 52.04 | 41.31 |
| Ligand/ion | 54.40 | 59.48 | 33.68 | 55.95 | 39.52 |
| Water | 52.34 | 59.97 | 41.79 | 42.67 | 35.84 |
| R.m.s deviations |  |  |  |  |  |
| Bond lengths (Å) | 0.009 | 0.009 | 0.008 | 0.008 | 0.009 |
| Bond angles (°) | 1.35 | 1.31 | 1.18 | 1.10 | 1.32 |
| Ramachandran |  |  |  |  |  |
| Favored (%)  Allowed (%) | 93.73  6.27 | 94.36  5.64 | 94.36  5.64 | 93.73  5.96 | 93.10  6.9 |
| Outliers (%) | 0 | 0 | 0 | 0.31 | 0 |

|  | DYRK2-C13 | DYRK2-C14 | DYRK2-C17 | DYRK2-C18 | DYRK2-C19 | DYRK2-C20 |
| --- | --- | --- | --- | --- | --- | --- |
| PDB code | 7DHK | 7DHO | 7DJO | 7DL6 | 7DHH | 7DHN |
| **Data collection** |  |  |  |  |  |  |
| Space group | C 2 2 21 | C 2 2 21 | C 2 2 21 | C 2 2 21 | C 2 2 21 | C 2 2 21 |
| Cell dimensions (Å)  α, β, γ (°) | 64.557 127.838 132.027  90, 90, 90 | 64.61 128.87 133.74  90, 90, 90 | 64.955 128.929 133.548  90, 90, 90 | 64.662 128.495 132.553  90, 90, 90 | 64.984 128.466 132.676  90, 90, 90 | 64.76 128.55 132.64  90, 90, 90 |
| Wavelength (Å) | 1.0000 | 1.0000 | 1.0000 | 1.0000 | 1.0000 | 1.0000 |
| Resolution (Å)  CC1/2  Rpim | 50.00-2.34(2.43-2.34)  0.971(0.588)  0.120(0.456) | 50.00-3.29(3.41-3.29)  0.925(0.676)  0.203(0.424) | 50.00-2.49(2.58-2.49)  0.994(0.903)  0.039(0.247) | 50.00-2.65(2.74-2.65)  0.991(0.816)  0.053(0.219) | 50.00-2.21(2.29-2.21)  0.997(0.755)  0.034(0.405) | 50.00-2.38(2.47-2.38)  0.999(0.800)  0.025(0.334) |
| *R*_merge_ | 0.257(1.017) | 0.483(1.013) | 0.134(0.829) | 0.149(0.452) | 0.106(1.379) | 0.083(1.133) |
| *I* / σ*I* | 6.0(2.2) | 3.6(2.2) | 24.1(4.1) | 13.0(4.0) | 17.3(2.2) | 18.9(2.3) |
| Completeness (%) | 99.8 (99.9) | 99.9(99.8) | 100.0 (100.0) | 99.2 (93.2) | 99.7 (100.0) | 99.8 (99.8) |
| redundancy | 6.4(6.2) | 6.6(6.8) | 13.0(12.3) | 8.3(4.9) | 12.8(13.6) | 13.2(13.3) |
| Wilson B-factor | 33.31 | 52.26 | 35.49 | 42.16 | 45.92 | 58.58 |
| **Refinement** |  |  |  |  |  |  |
| No. reflections | 17793 | 8788 | 19677 | 15541 | 20545 | 22545 |
| *R*_work_ / *R*_free_ | 0.195 / 0.257 | 0.184 / 0.254 | 0.199/ 0.239 | 0.173 / 0.238 | 0.213 / 0.213 | 0.185 / 0.223 |
| No. of atoms |  |  |  |  |  |  |
| Protein | 2646 | 2646 | 2646 | 2646 | 2646 | 2646 |
| Ligand/ion | 32 | 28 | 27 | 27 | 26 | 25 |
| Water | 69 | 0 | 112 | 71 | 45 | 40 |
| *B*-factors |  |  |  |  |  |  |
| Macromolecules | 43.72 | 45.46 | 43.94 | 46.27 | 57.45 | 68.62 |
| Ligand/ion | 46.42 | 49.17 | 37.16 | 39.71 | 50.17 | 64.31 |
| Water | 38.66 | 0 | 41.16 | 43.68 | 48.11 | 57.95 |
| R.m.s deviations |  |  |  |  |  |  |
| Bond lengths (Å) | 0.008 | 0.011 | 0.009 | 0.008 | 0.009 | 0.009 |
| Bond angles (°) | 1.14 | 1.40 | 1.08 | 1.00 | 1.05 | 1.09 |
| Ramachandran |  |  |  |  |  |  |
| Favored (%)  Allowed (%) | 94.36  5.02 | 90.28  9.09 | 94.04  5.64 | 91.85  7.84 | 91.22  8.46 | 93.73  6.27 |
| Outliers (%) | 0.63 | 0.63 | 0.31 | 0.31 | 0.31 | 0 |

**Figure 1-source data 1.**  Data collection and refinement statistics. Each dataset was collected from a single crystal. Values in parentheses are for highest-resolution shell
