# Supplementary figures and images for: Selective inhibition reveals the regulatory function of DYRK2 in protein synthesis and calcium entry
Source: eLife. 2022 Apr 19;11:e77696. doi: 10.7554/eLife.77696 (PMC9113749; doi:10.7554/eLife.77696)

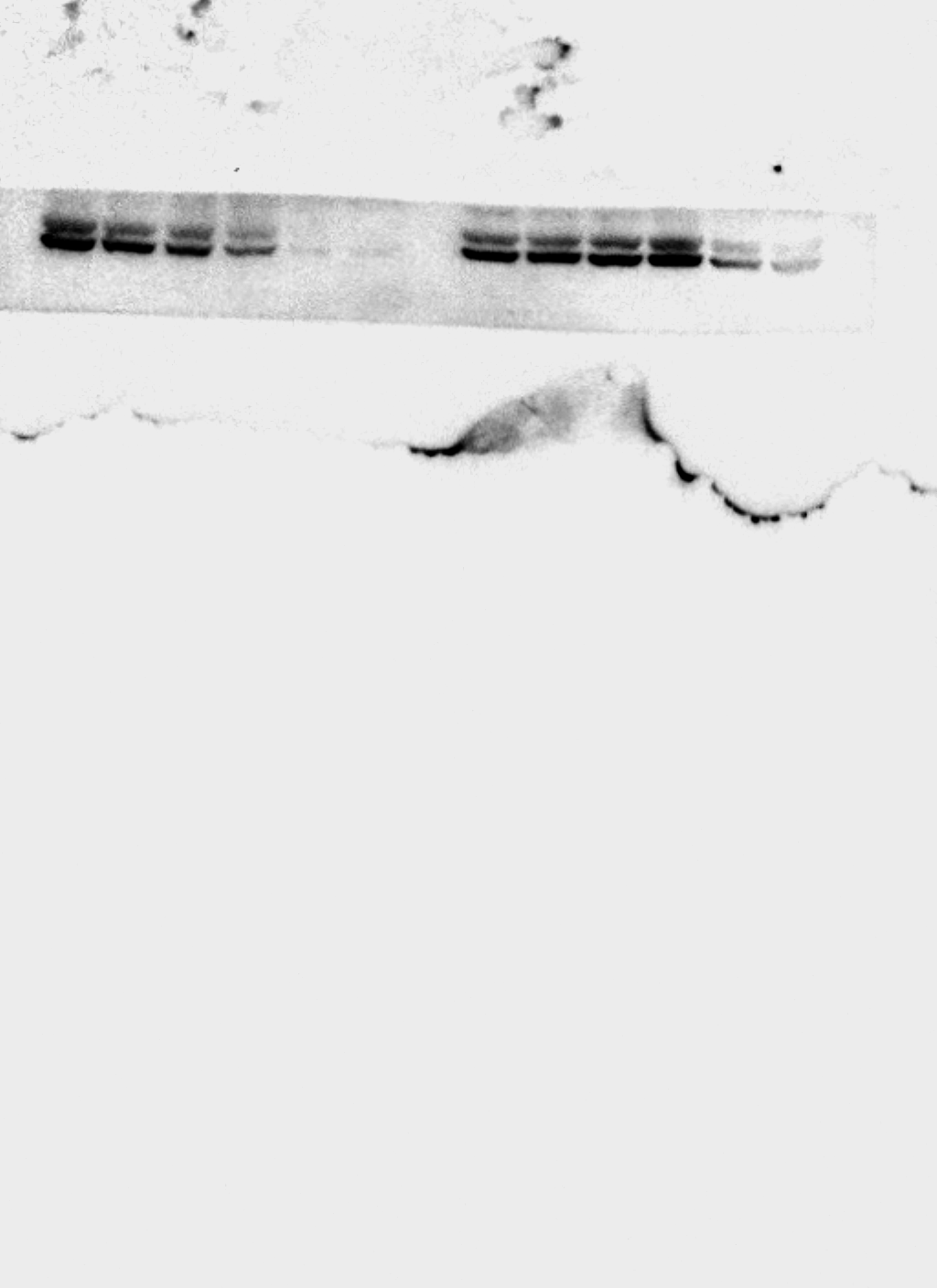

Supplement: Figure 1—figure supplement 1—source data 1. [file elife-77696-fig1-figsupp1-data1.zip › Figure 1-figure supplement 1-source data 1/pT25_sourcedata.tif]

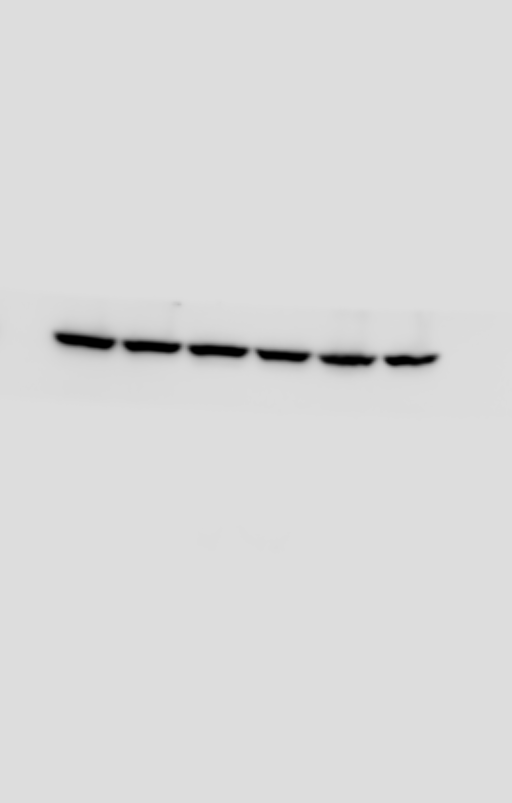

Supplement: Figure 1—figure supplement 1—source data 1. [file elife-77696-fig1-figsupp1-data1.zip › Figure 1-figure supplement 1-source data 1/RPT3_sourcedata.tif]

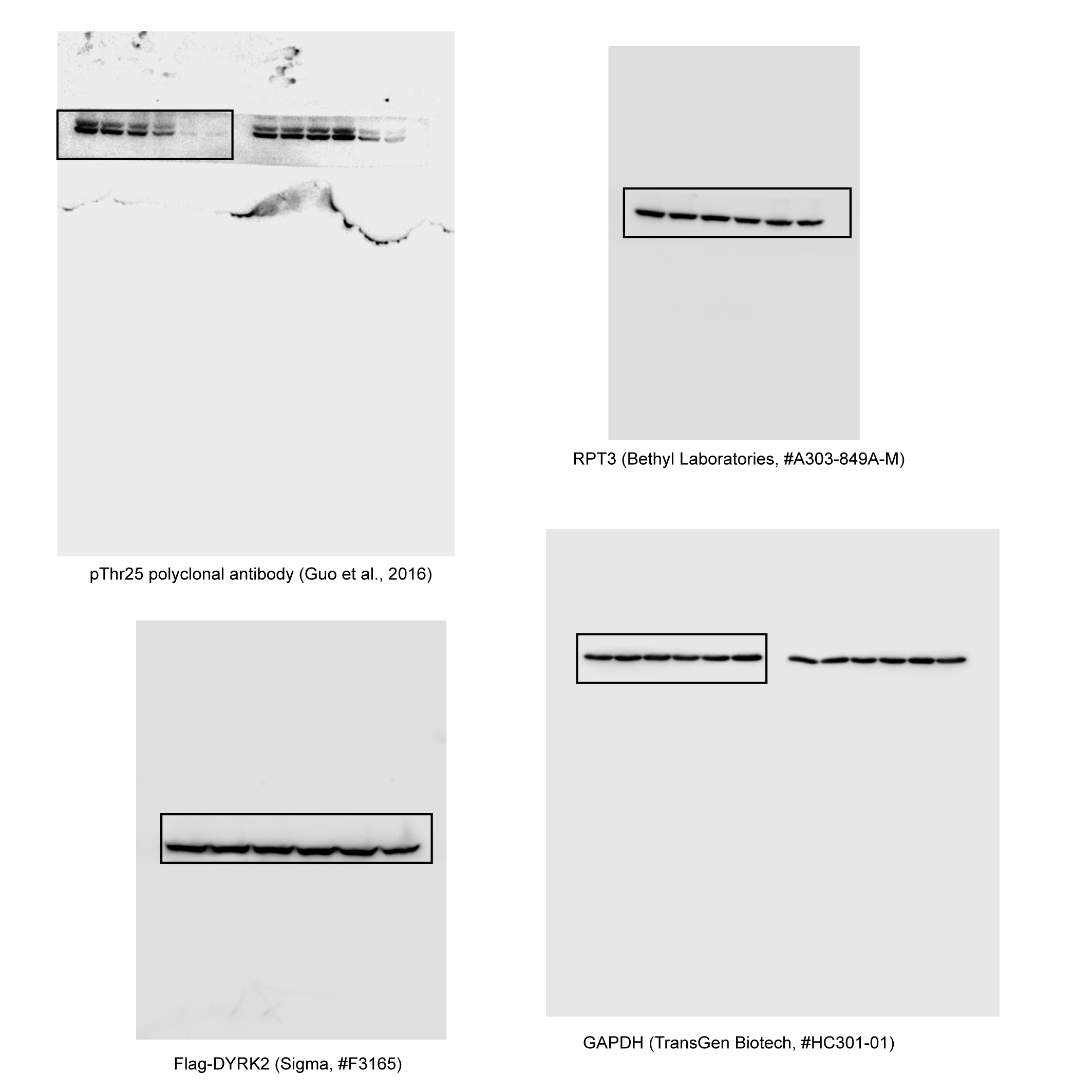

Supplement: Figure 1—figure supplement 1—source data 1. [file elife-77696-fig1-figsupp1-data1.zip › Figure 1-figure supplement 1-source data 1/Uncropped_Labeled_Gels_Fig1_figuresupplement1B.tif]

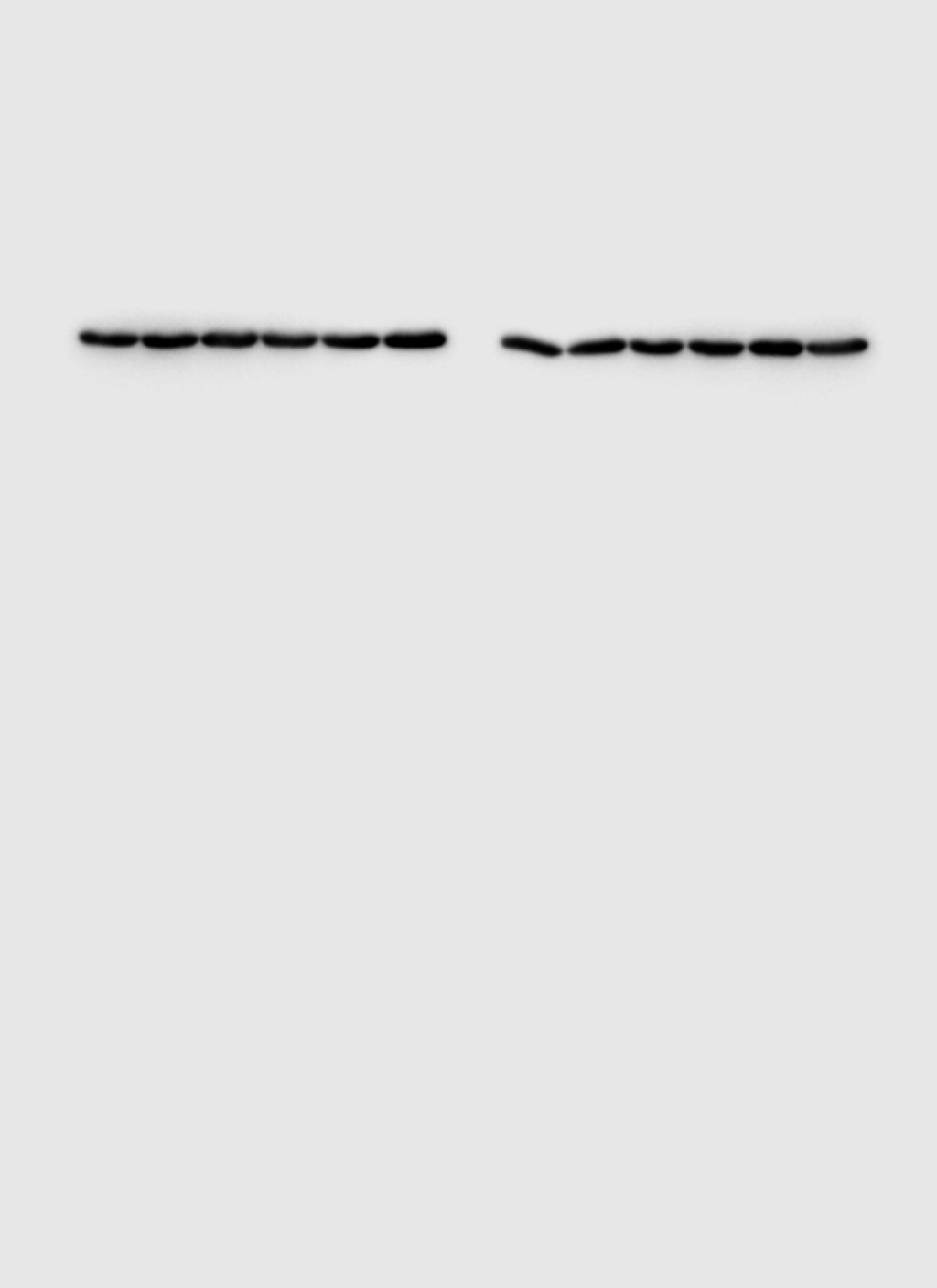

Supplement: Figure 1—figure supplement 1—source data 1. [file elife-77696-fig1-figsupp1-data1.zip › Figure 1-figure supplement 1-source data 1/GAPDH_sourcedata.tif]

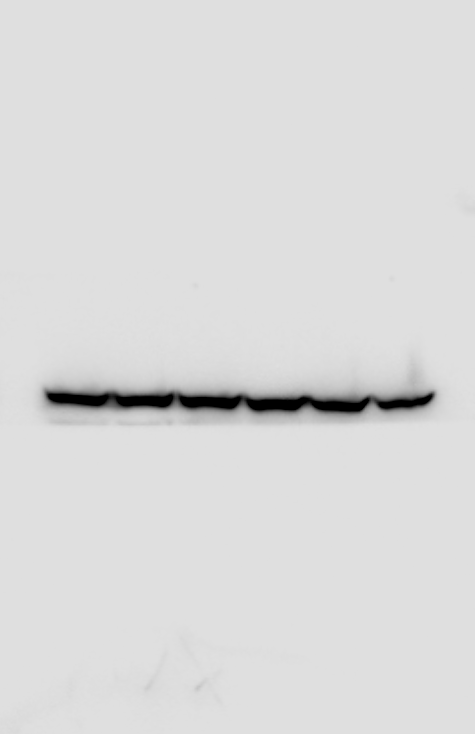

Supplement: Figure 1—figure supplement 1—source data 1. [file elife-77696-fig1-figsupp1-data1.zip › Figure 1-figure supplement 1-source data 1/FLAG_sourcedata.tif]

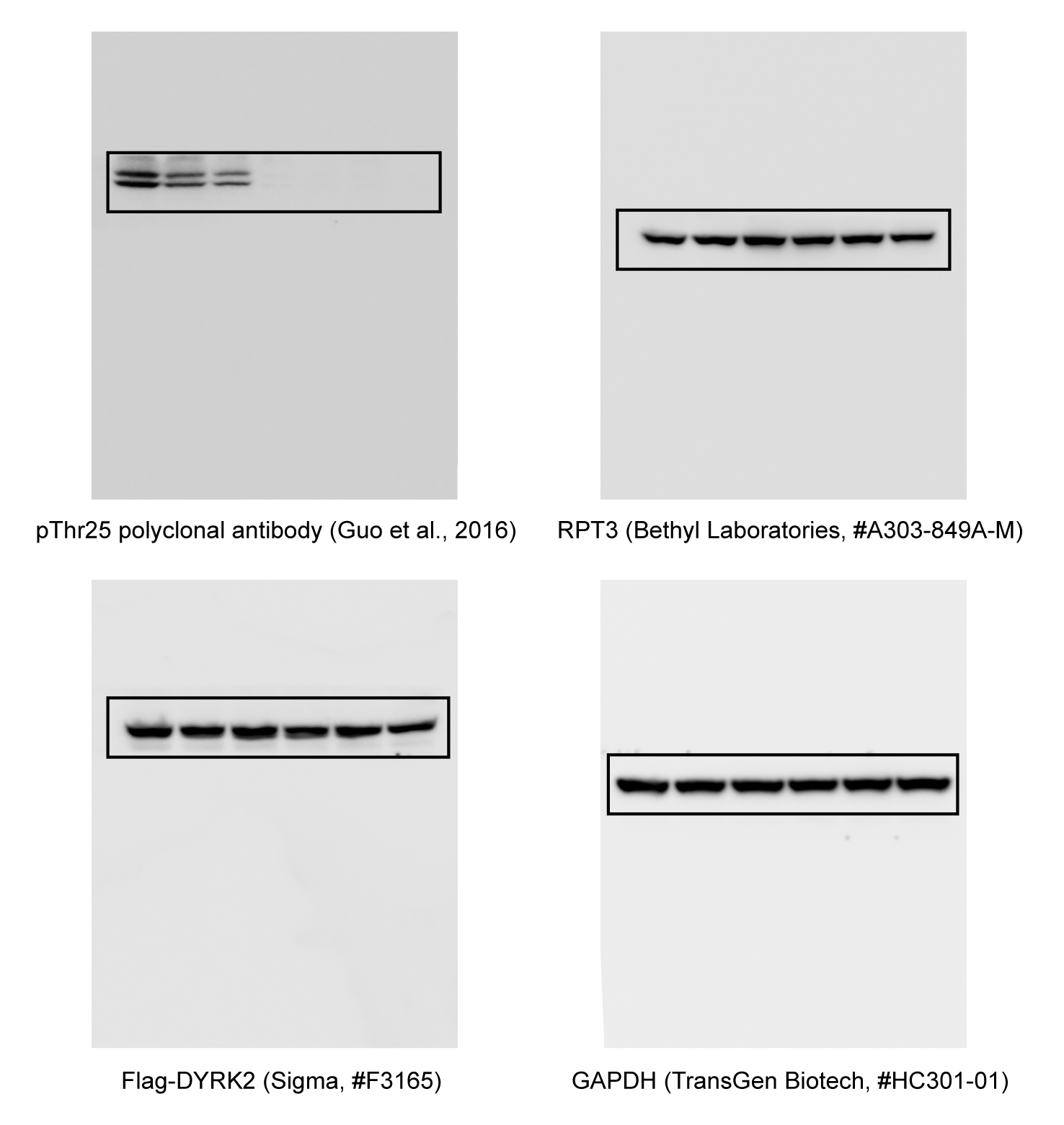

Supplement: Figure 2—source data 2. [file elife-77696-fig2-data2.zip › Figure2-source data 2/Uncropped_Labeled_Gels_Fig2D.tif]

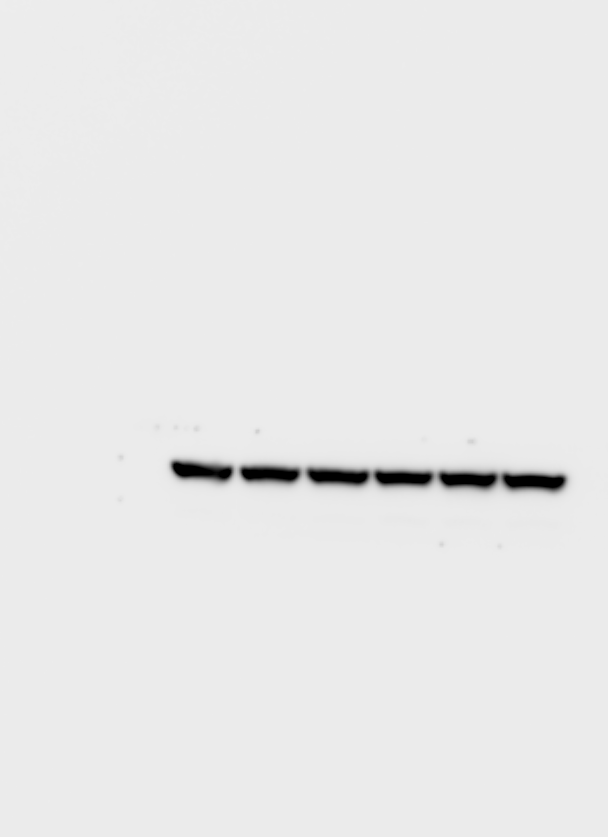

Supplement: Figure 2—source data 2. [file elife-77696-fig2-data2.zip › Figure2-source data 2/GAPDHWB_sourcedata.tif]

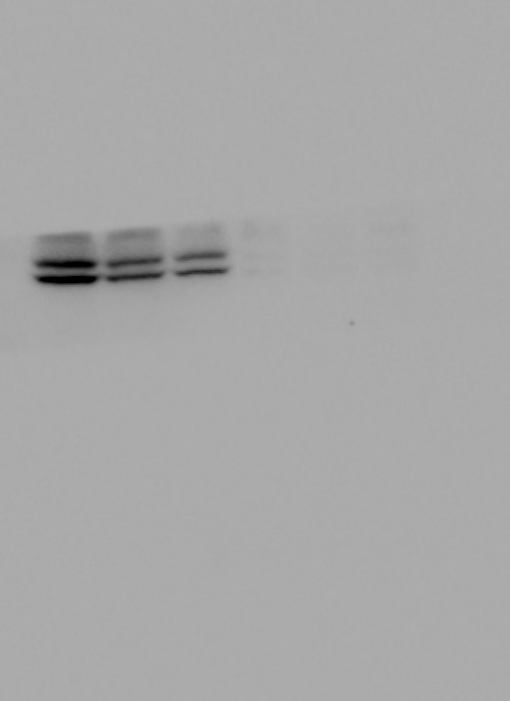

Supplement: Figure 2—source data 2. [file elife-77696-fig2-data2.zip › Figure2-source data 2/pT25_sourcedata.tif]

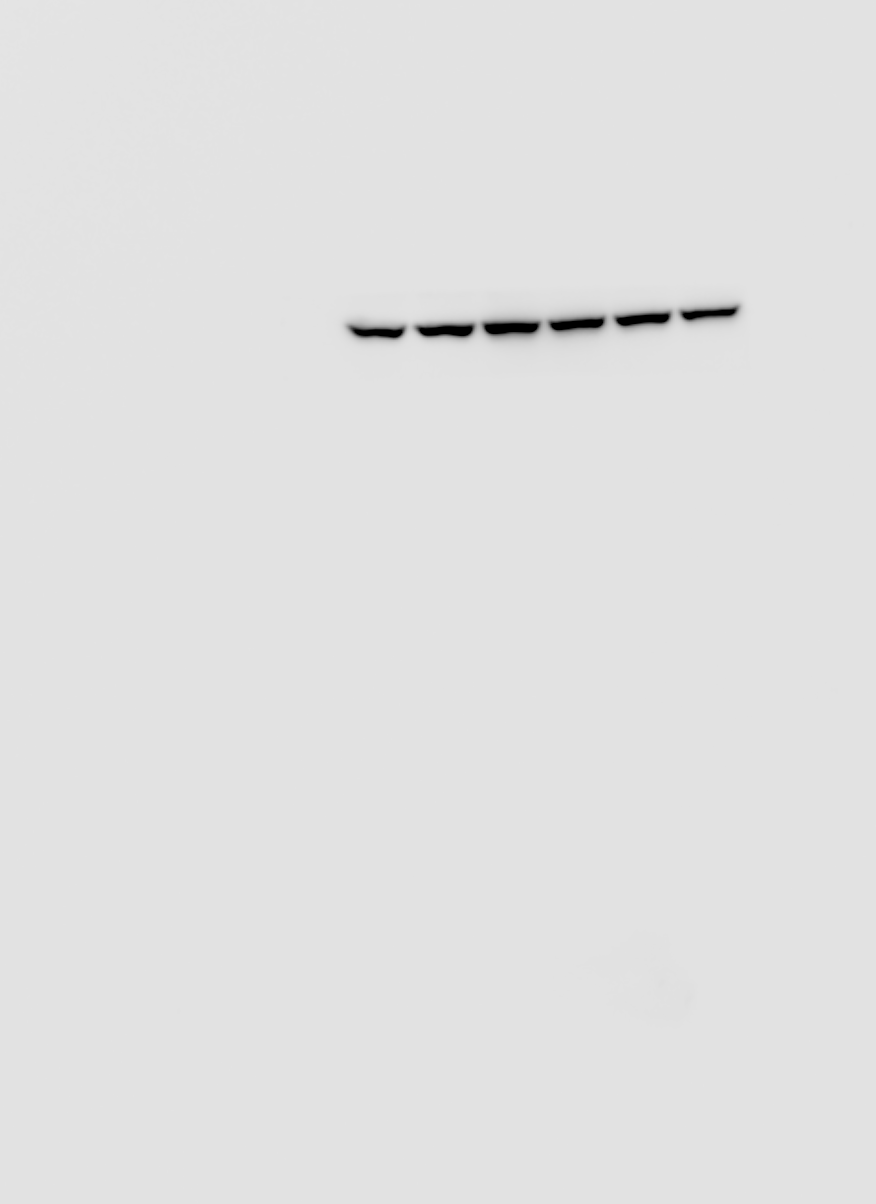

Supplement: Figure 2—source data 2. [file elife-77696-fig2-data2.zip › Figure2-source data 2/RPT3WB_sourcedata.tif]

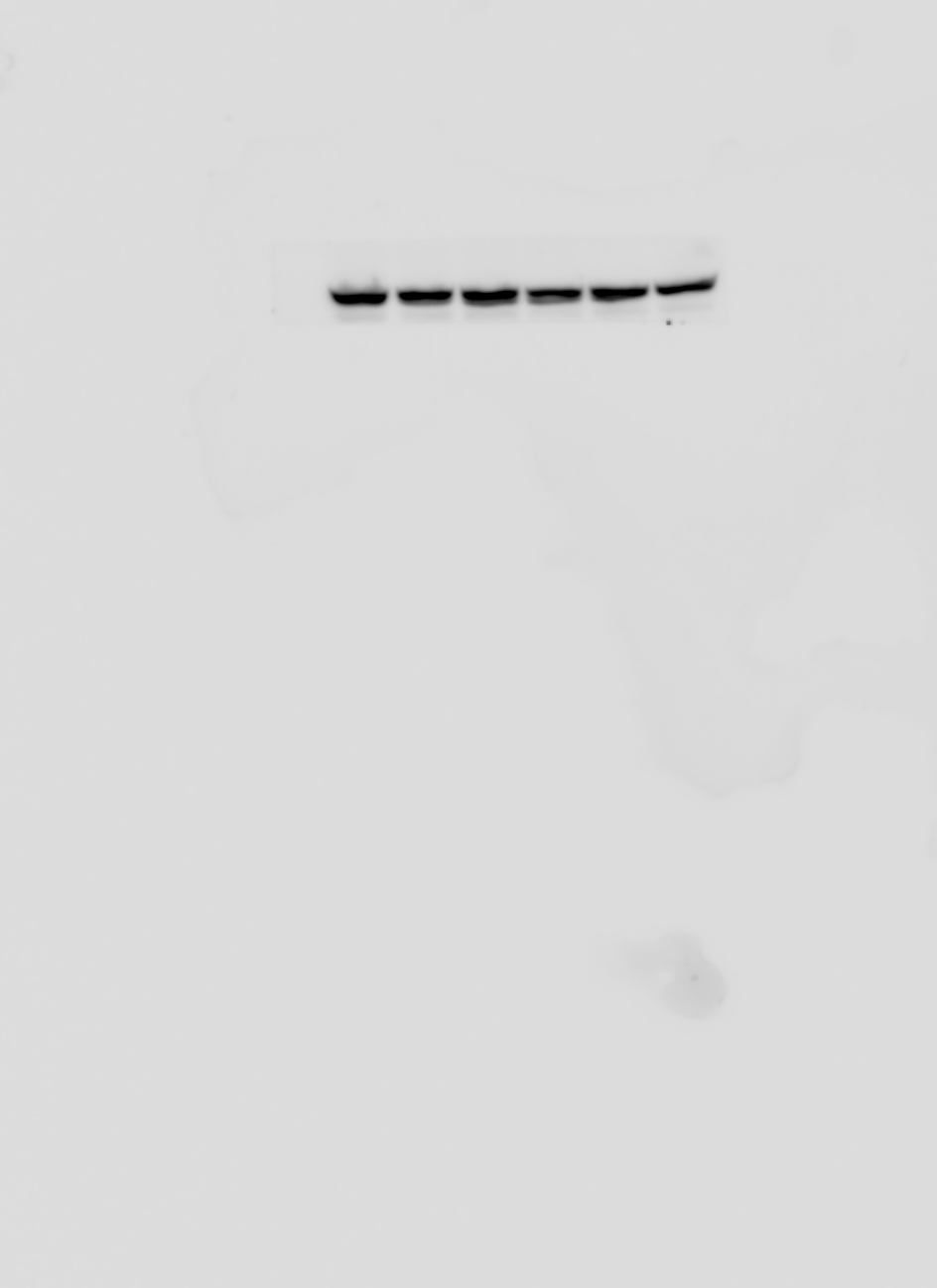

Supplement: Figure 2—source data 2. [file elife-77696-fig2-data2.zip › Figure2-source data 2/FlagWB_sourcedata.tif]

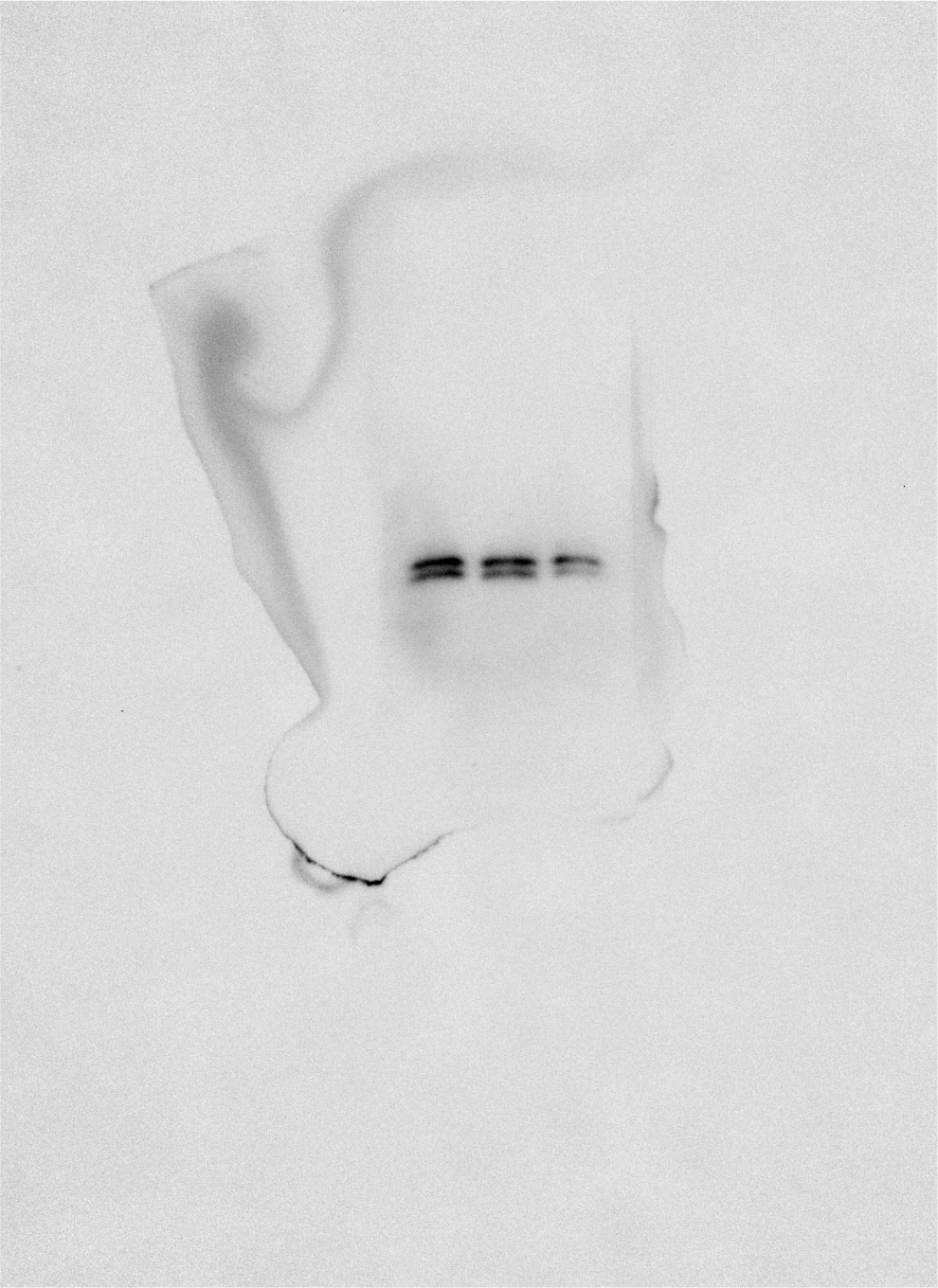

Supplement: Figure 4—source data 1. [file elife-77696-fig4-data1.zip › Figure 4-source data 1/Figure4A-sourcedata/4EBP1-pT70WB_sourcedata.tif]

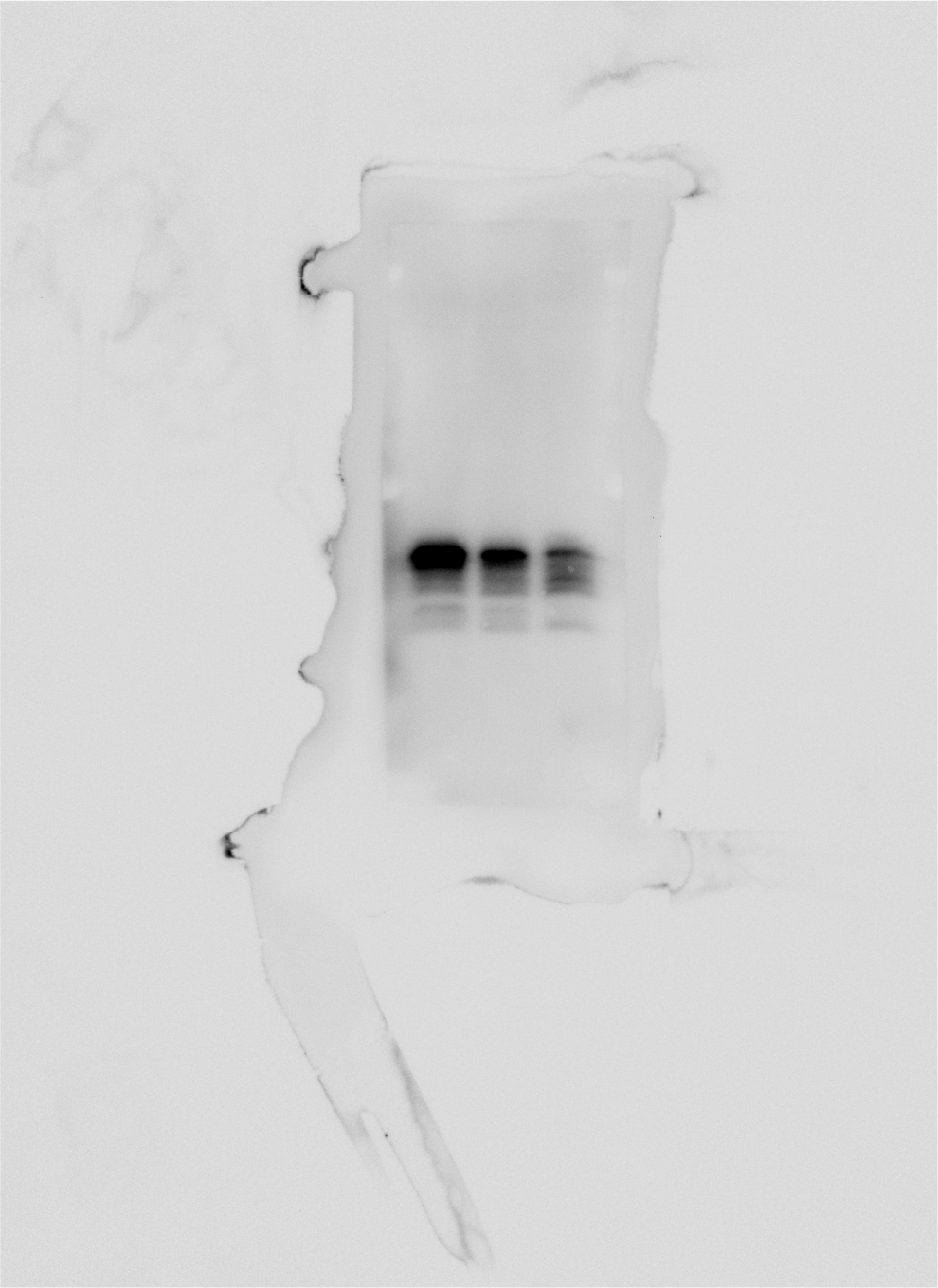

Supplement: Figure 4—source data 1. [file elife-77696-fig4-data1.zip › Figure 4-source data 1/Figure4A-sourcedata/4EBP1-pT37:46WB_sourcedata.tif]

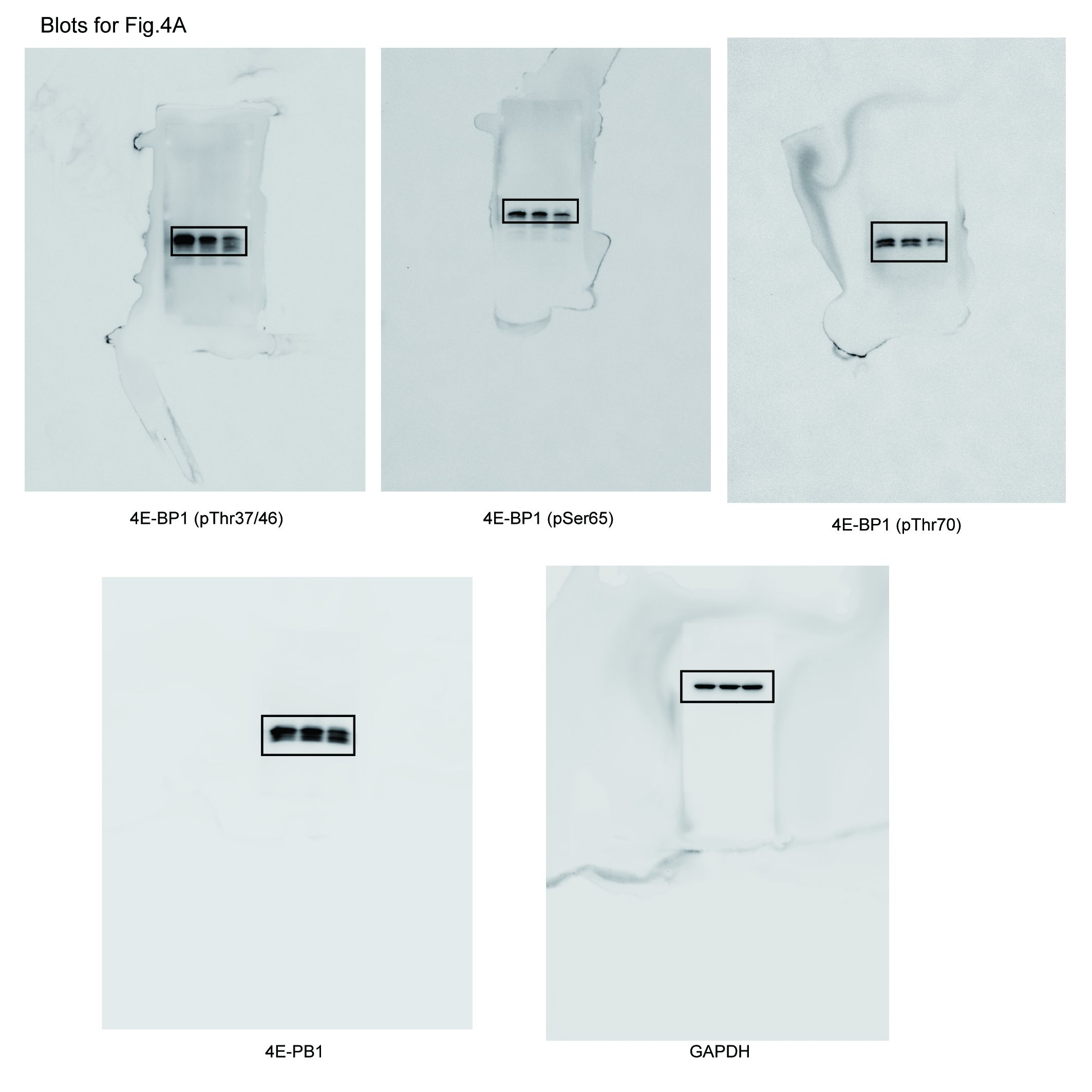

Supplement: Figure 4—source data 1. [file elife-77696-fig4-data1.zip › Figure 4-source data 1/Figure4A-sourcedata/Uncropped_Labeled_Gels_Fig4A.tif]

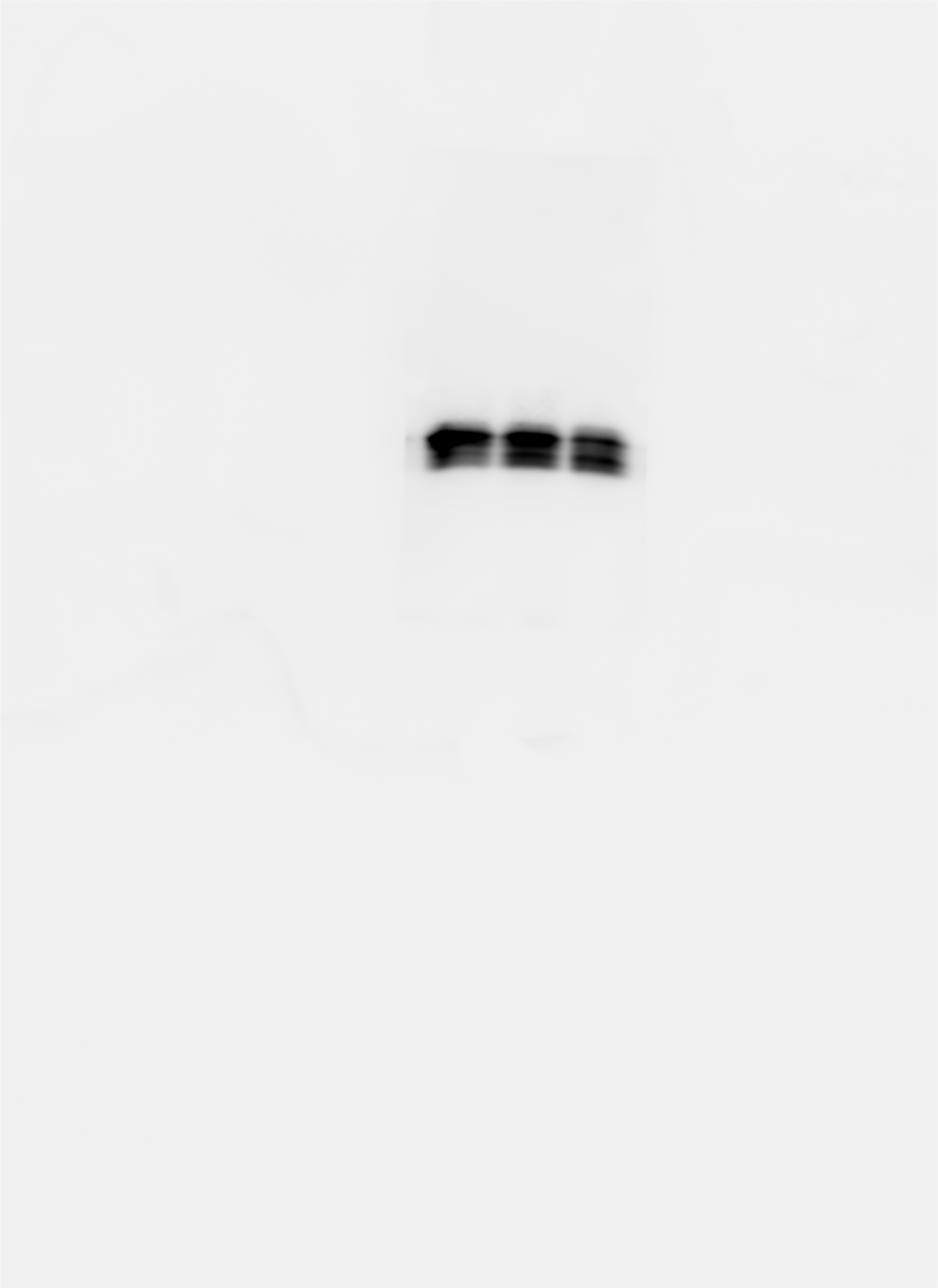

Supplement: Figure 4—source data 1. [file elife-77696-fig4-data1.zip › Figure 4-source data 1/Figure4A-sourcedata/4EBP1WB_sourcedata.tif]

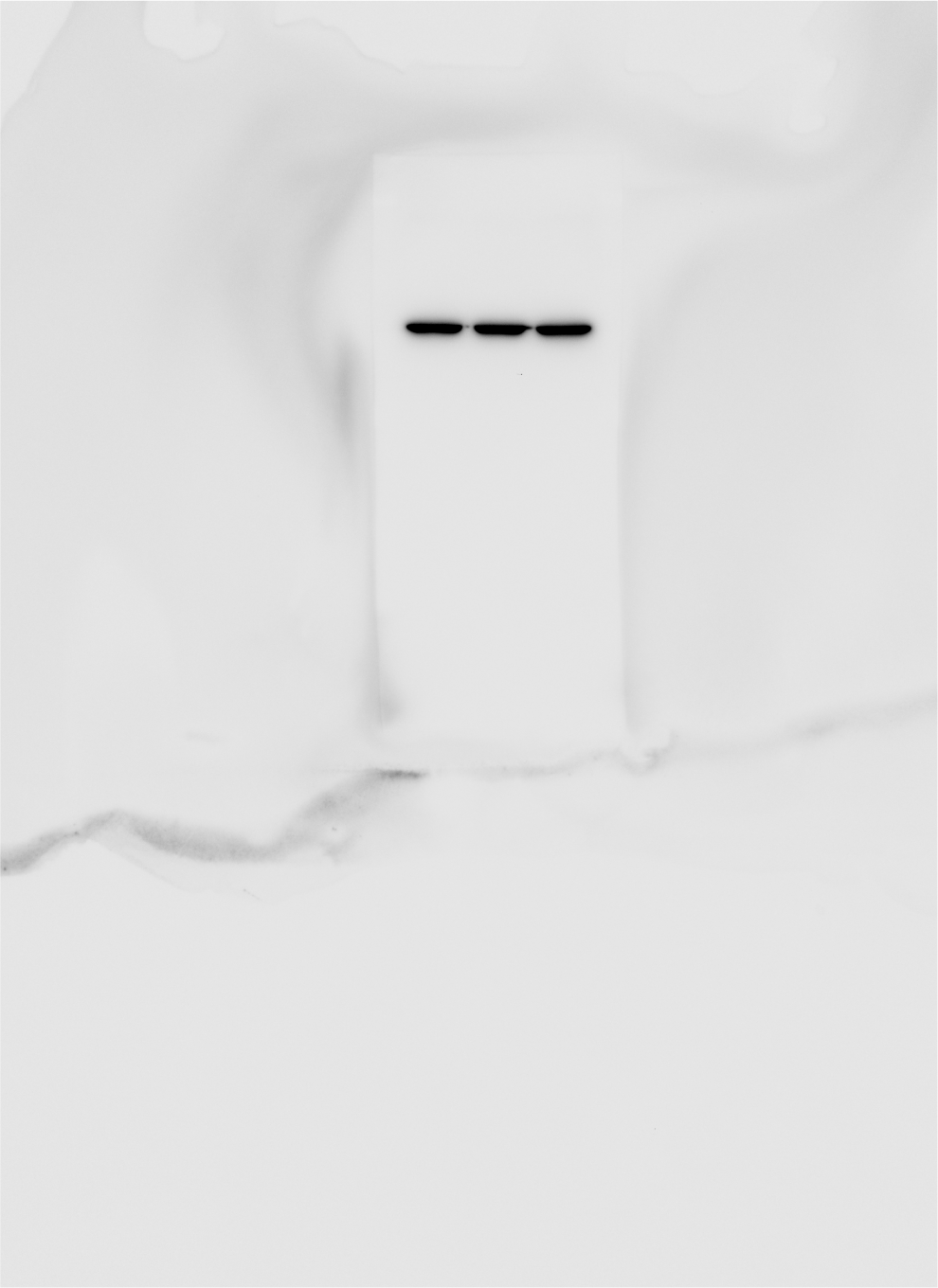

Supplement: Figure 4—source data 1. [file elife-77696-fig4-data1.zip › Figure 4-source data 1/Figure4A-sourcedata/GAPDH_WB_sourcedata.tif]

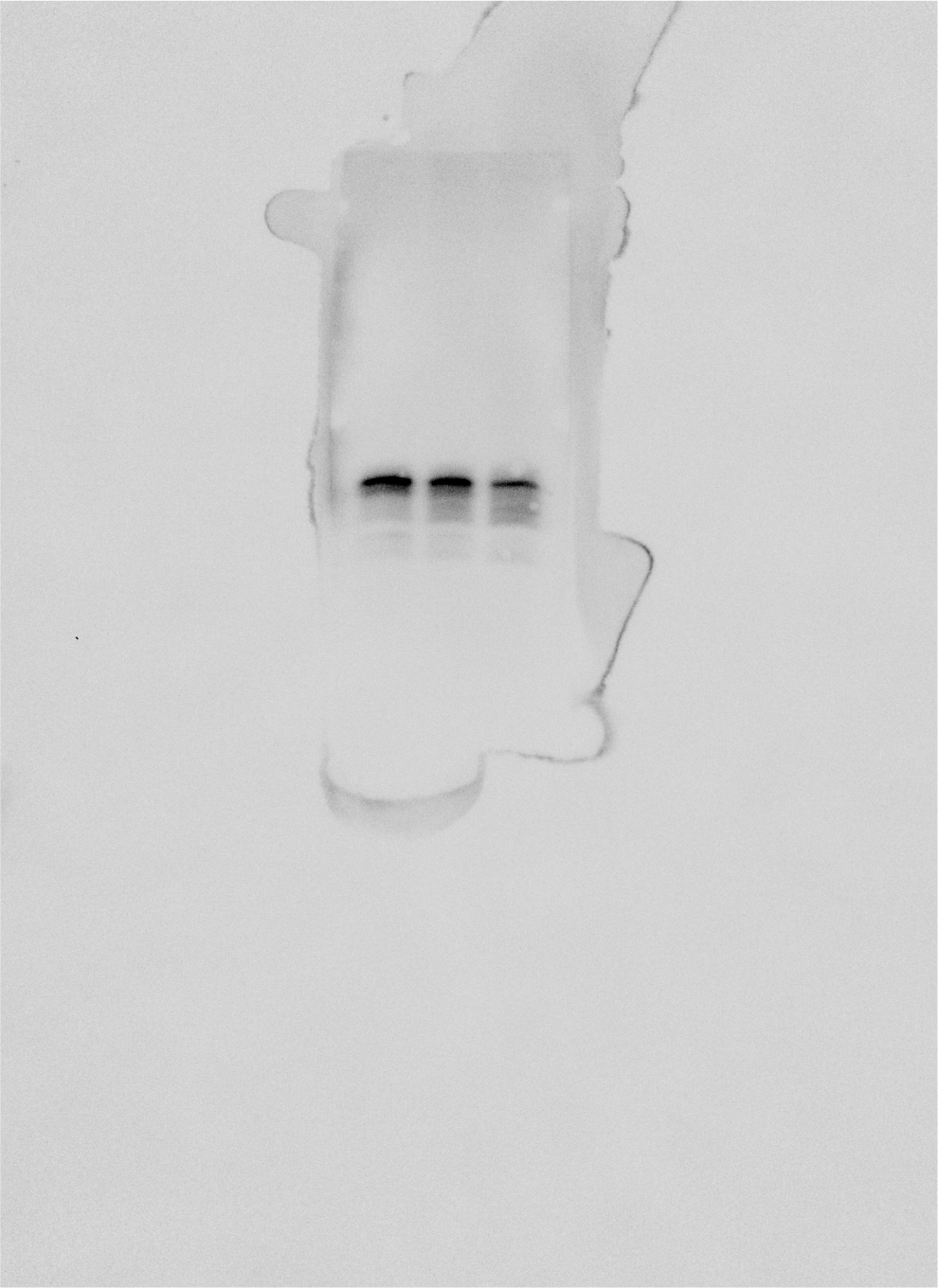

Supplement: Figure 4—source data 1. [file elife-77696-fig4-data1.zip › Figure 4-source data 1/Figure4A-sourcedata/4EBP1-pS65WB_sourcedata.tif]

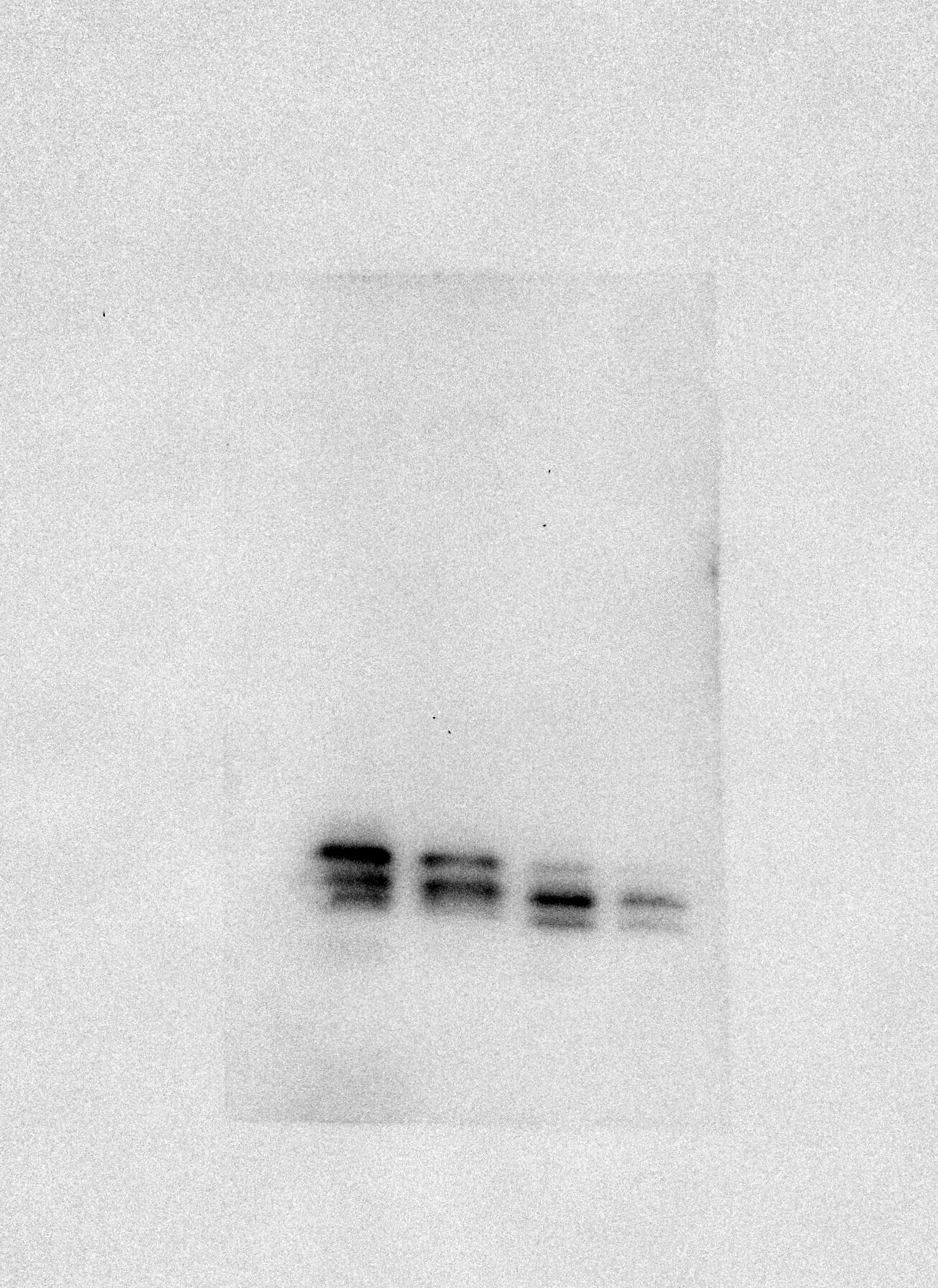

Supplement: Figure 4—source data 1. [file elife-77696-fig4-data1.zip › Figure 4-source data 1/Figure4B-sourcedata/4EBP1-pT70WB_sourcedata.tif]

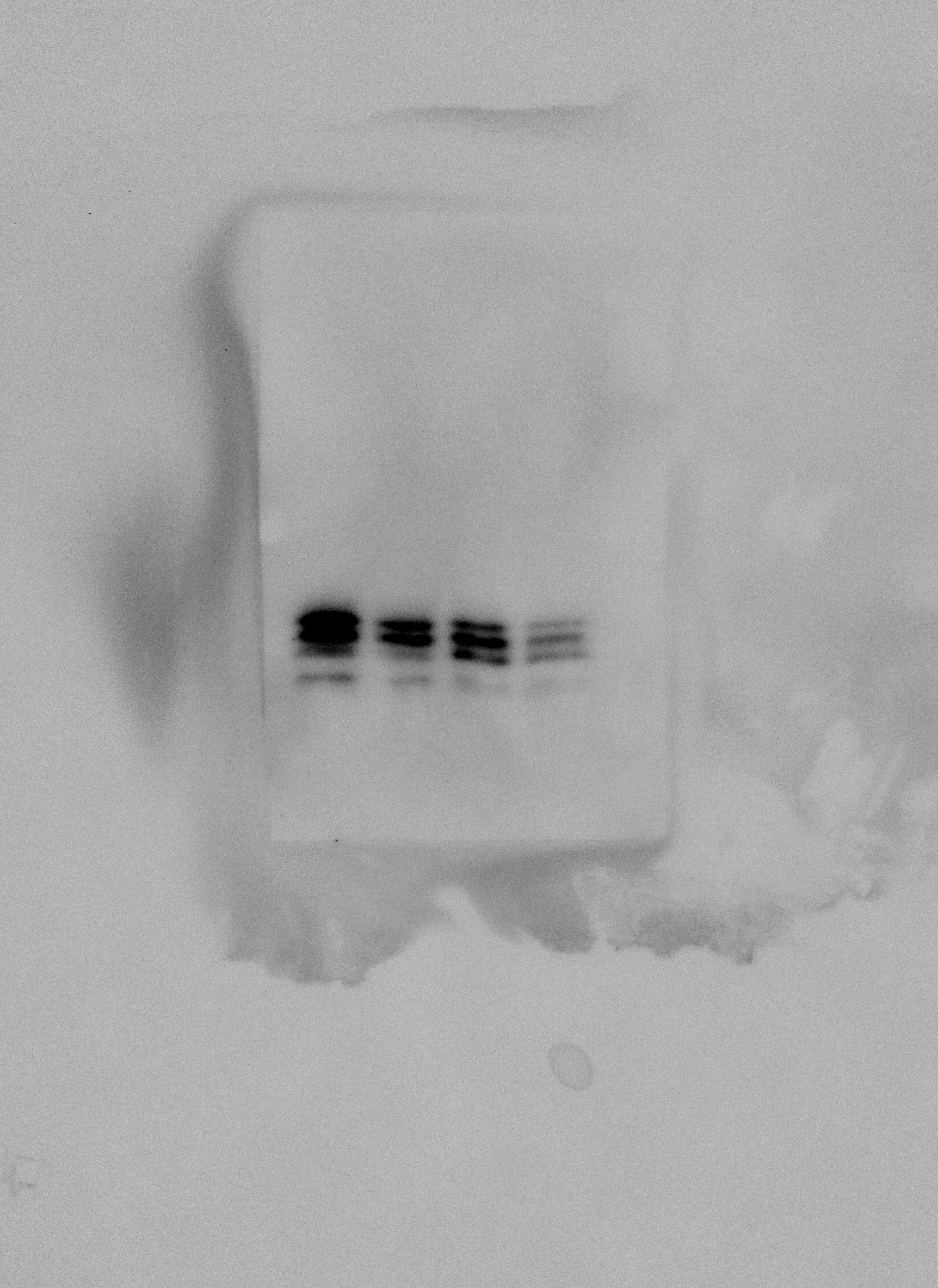

Supplement: Figure 4—source data 1. [file elife-77696-fig4-data1.zip › Figure 4-source data 1/Figure4B-sourcedata/4EBP1-pT37:46WB_sourcedata.tif]

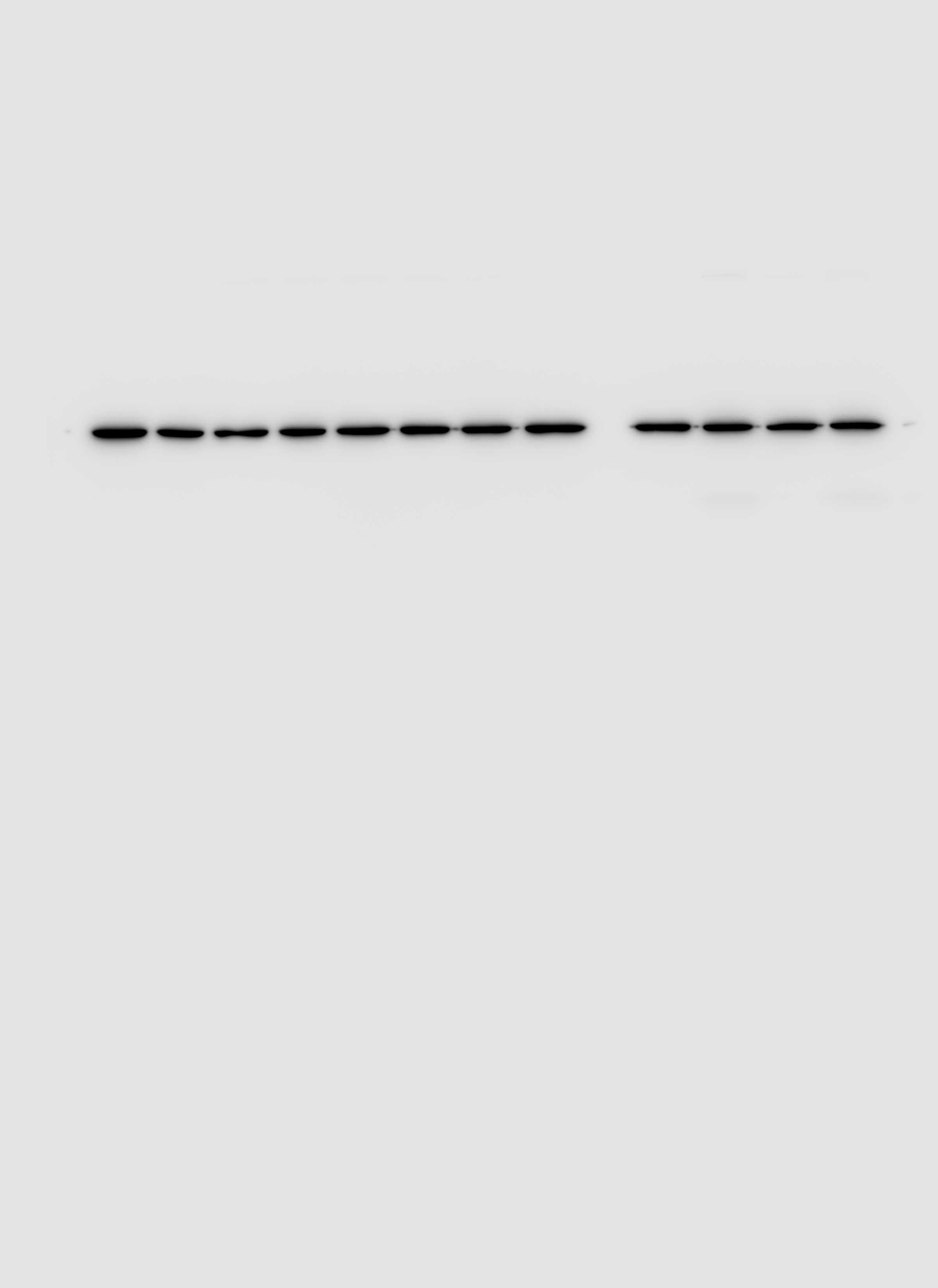

Supplement: Figure 4—source data 1. [file elife-77696-fig4-data1.zip › Figure 4-source data 1/Figure4B-sourcedata/GAPDHWB_sourcedata.tif]

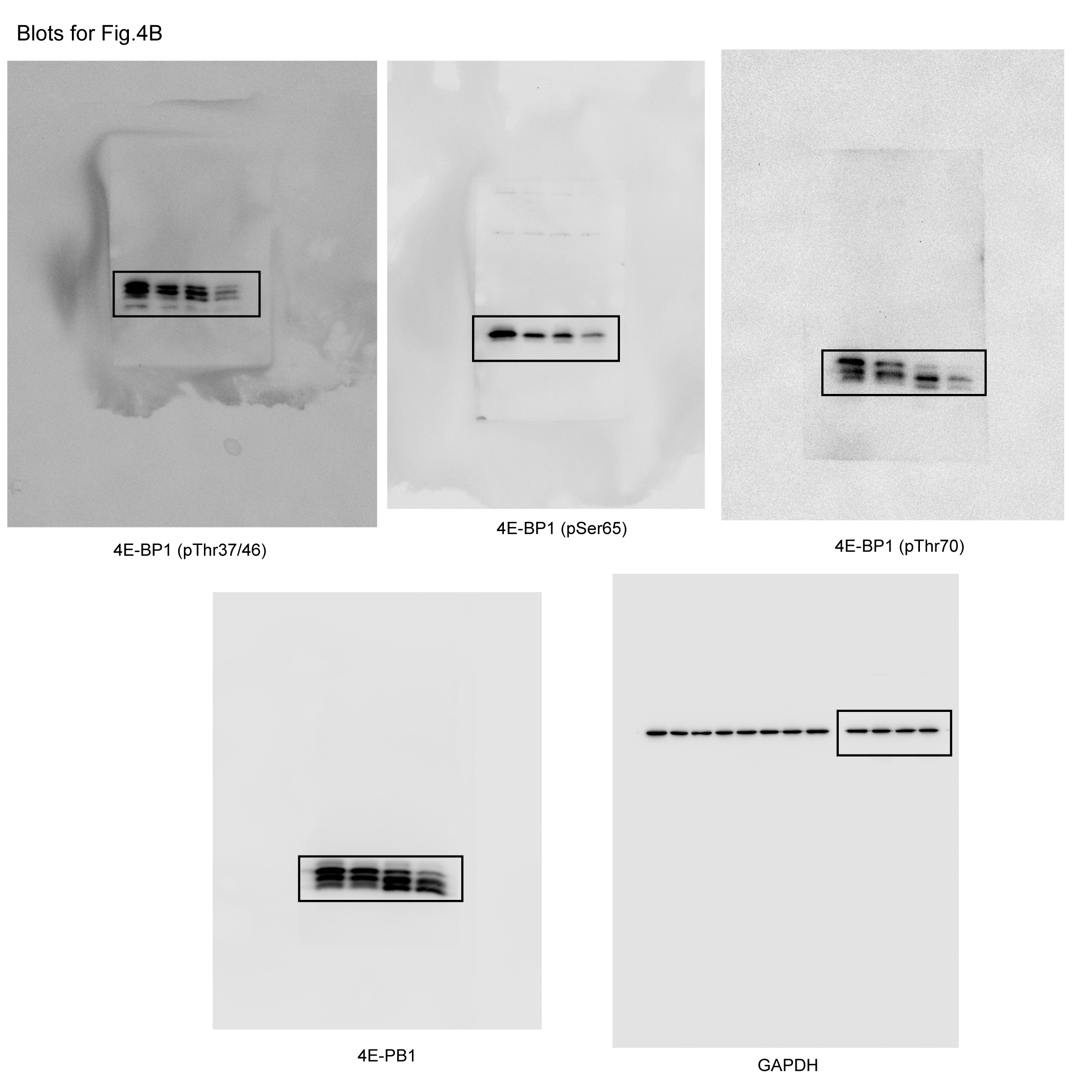

Supplement: Figure 4—source data 1. [file elife-77696-fig4-data1.zip › Figure 4-source data 1/Figure4B-sourcedata/Uncropped_Labeled_Gels_Fig4B.tif]

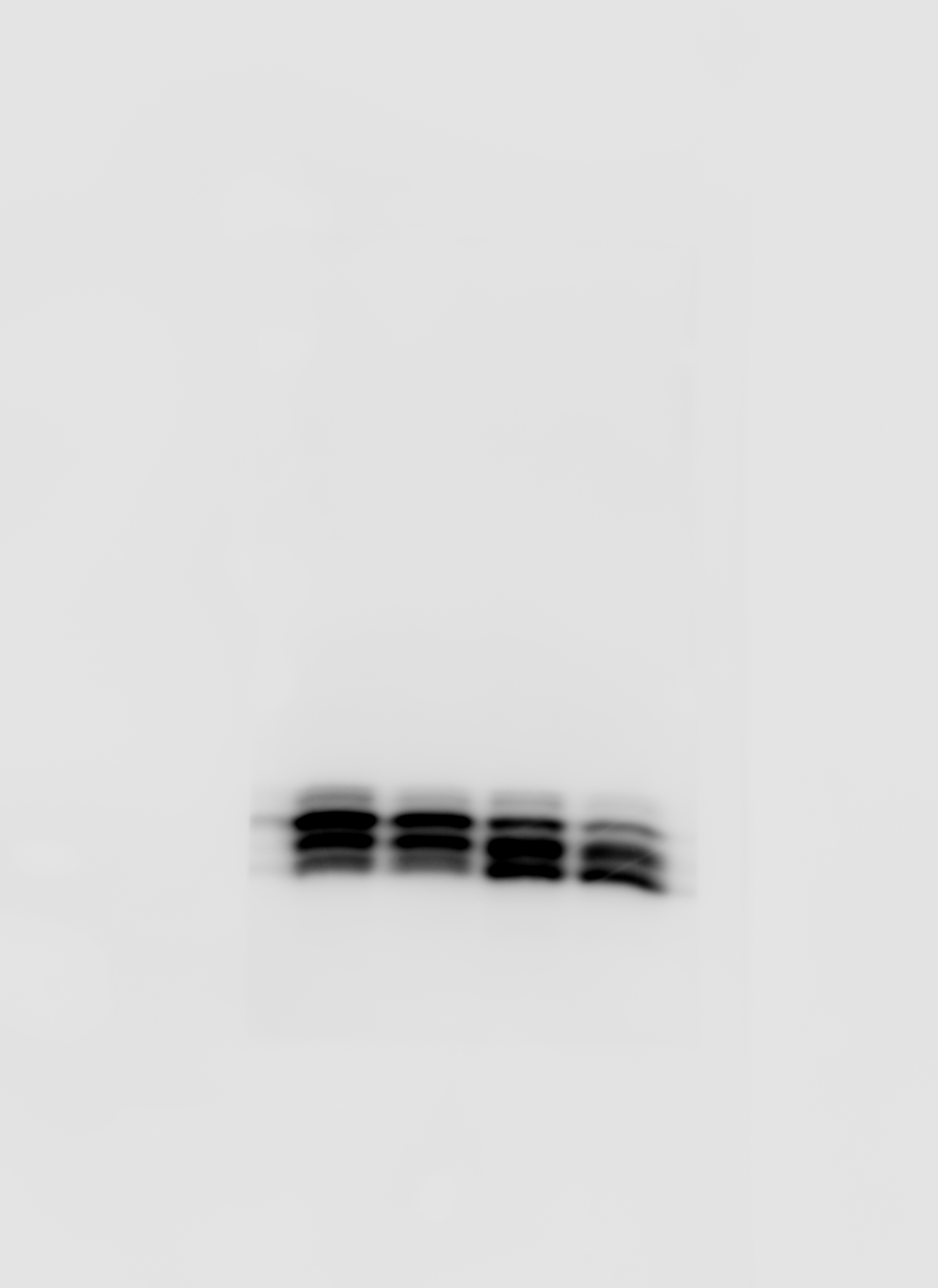

Supplement: Figure 4—source data 1. [file elife-77696-fig4-data1.zip › Figure 4-source data 1/Figure4B-sourcedata/4EBP1WB_sourcedata.tif]

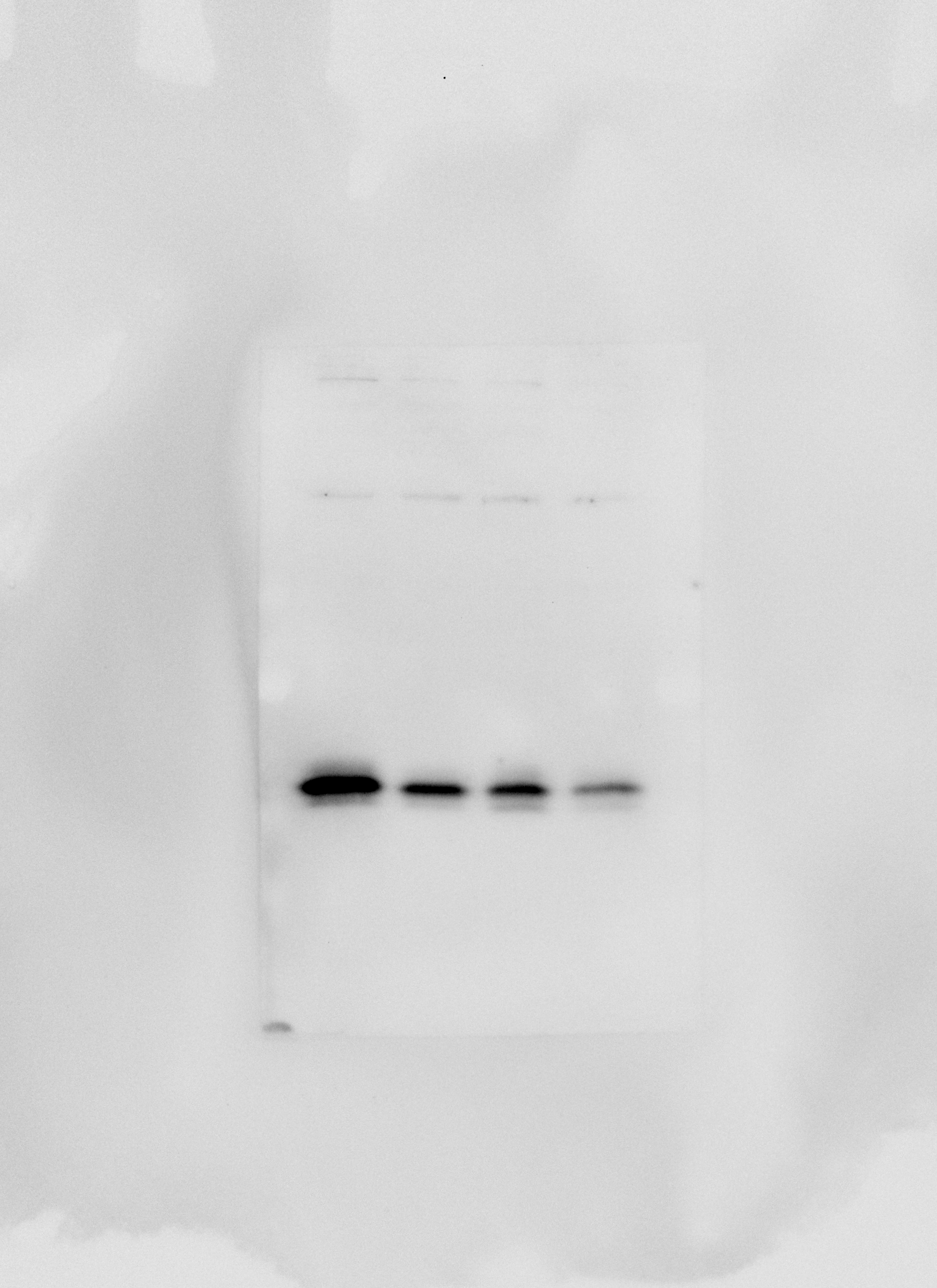

Supplement: Figure 4—source data 1. [file elife-77696-fig4-data1.zip › Figure 4-source data 1/Figure4B-sourcedata/4EBP1-pS65WB_sourcedata.tif]

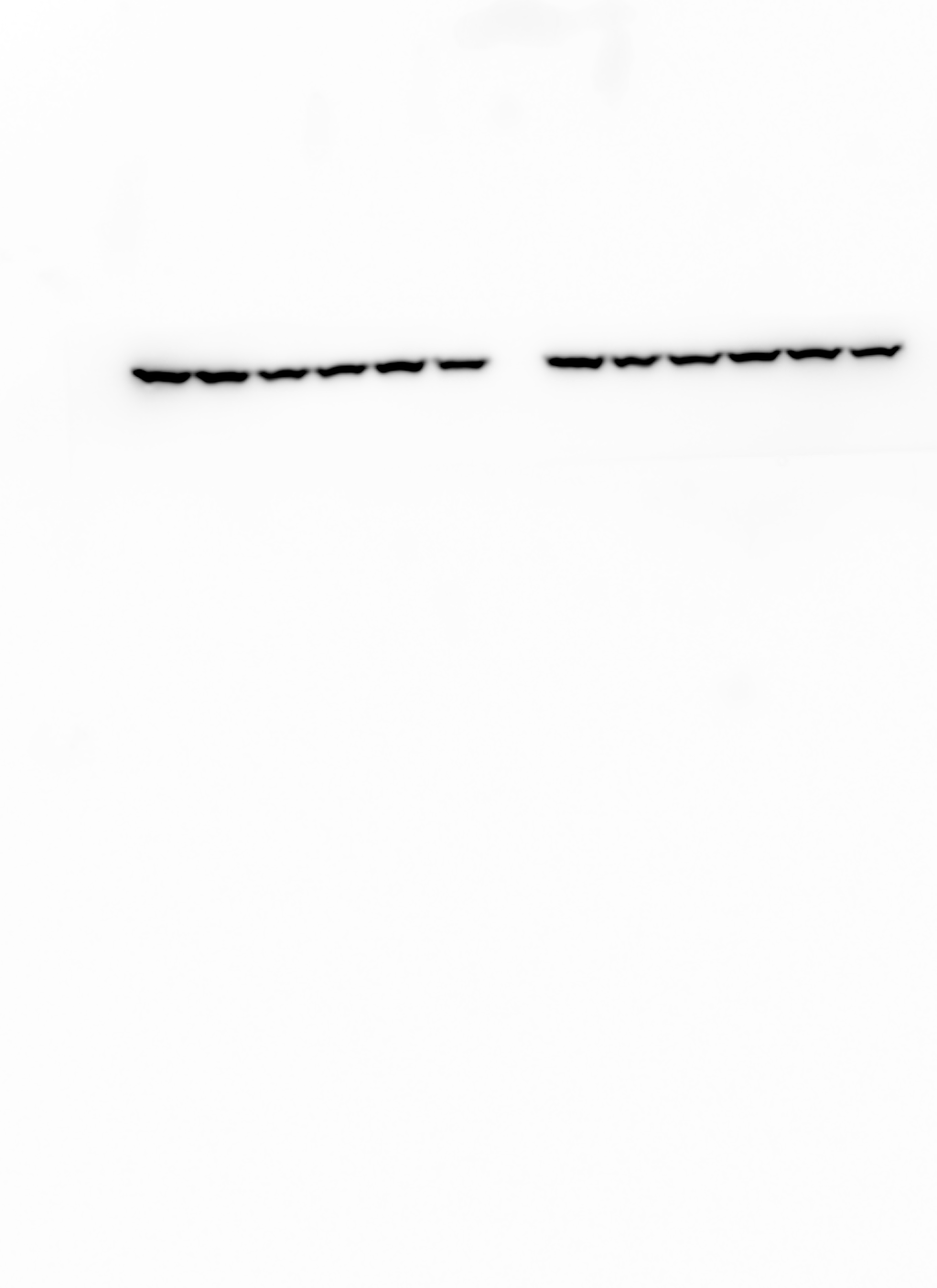

Supplement: Figure 4—source data 2. [file elife-77696-fig4-data2.zip › Figure 4-source data 2/Figure4C-sourcedata/HAWB_sourcedata.tif]

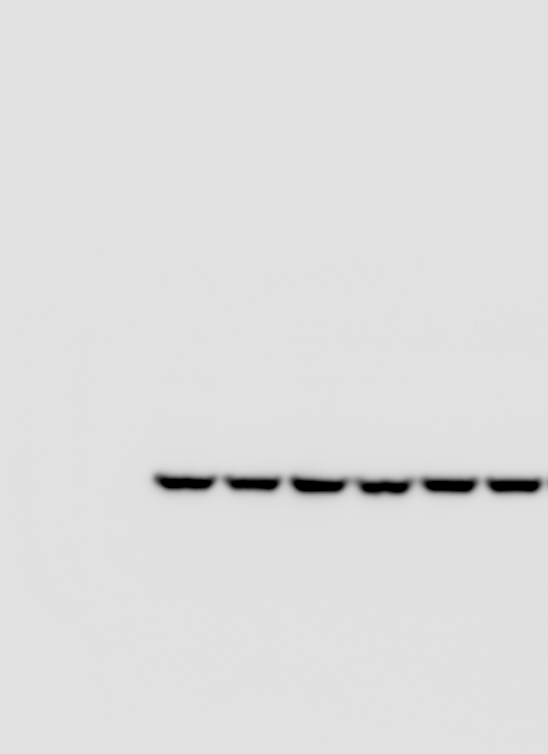

Supplement: Figure 4—source data 2. [file elife-77696-fig4-data2.zip › Figure 4-source data 2/Figure4C-sourcedata/4EBP1-FLAGWB_sourcedata.tif]

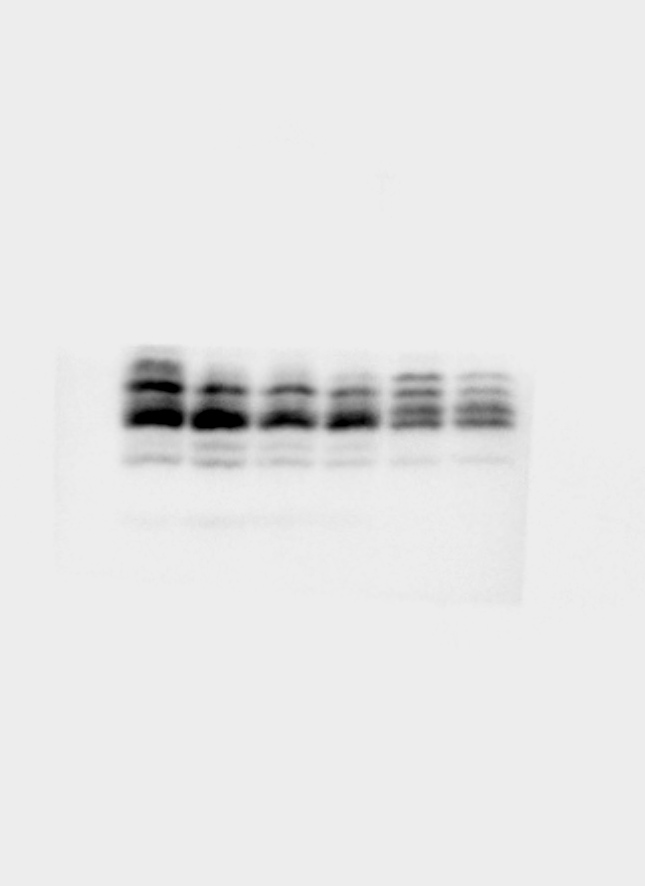

Supplement: Figure 4—source data 2. [file elife-77696-fig4-data2.zip › Figure 4-source data 2/Figure4C-sourcedata/4EBP1-pT37:46WB_sourcedata.tif]

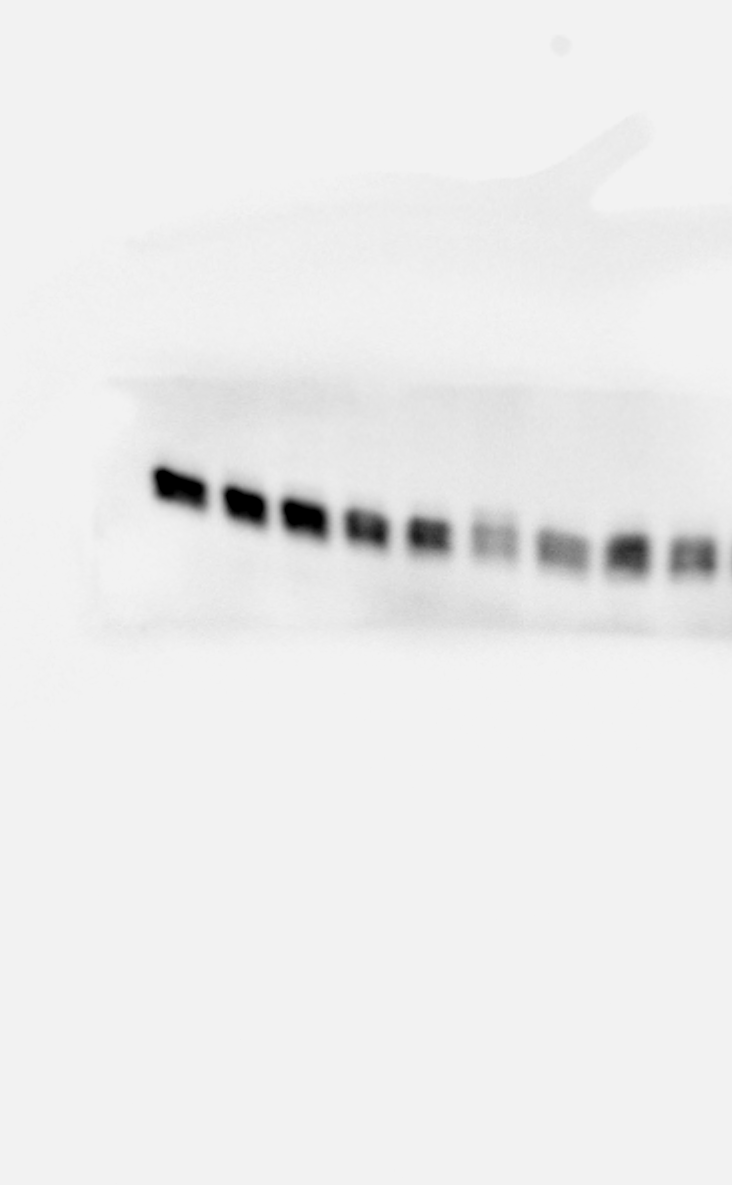

Supplement: Figure 4—source data 2. [file elife-77696-fig4-data2.zip › Figure 4-source data 2/Figure4C-sourcedata/4EBP1-pT70_sourcedata.tif]

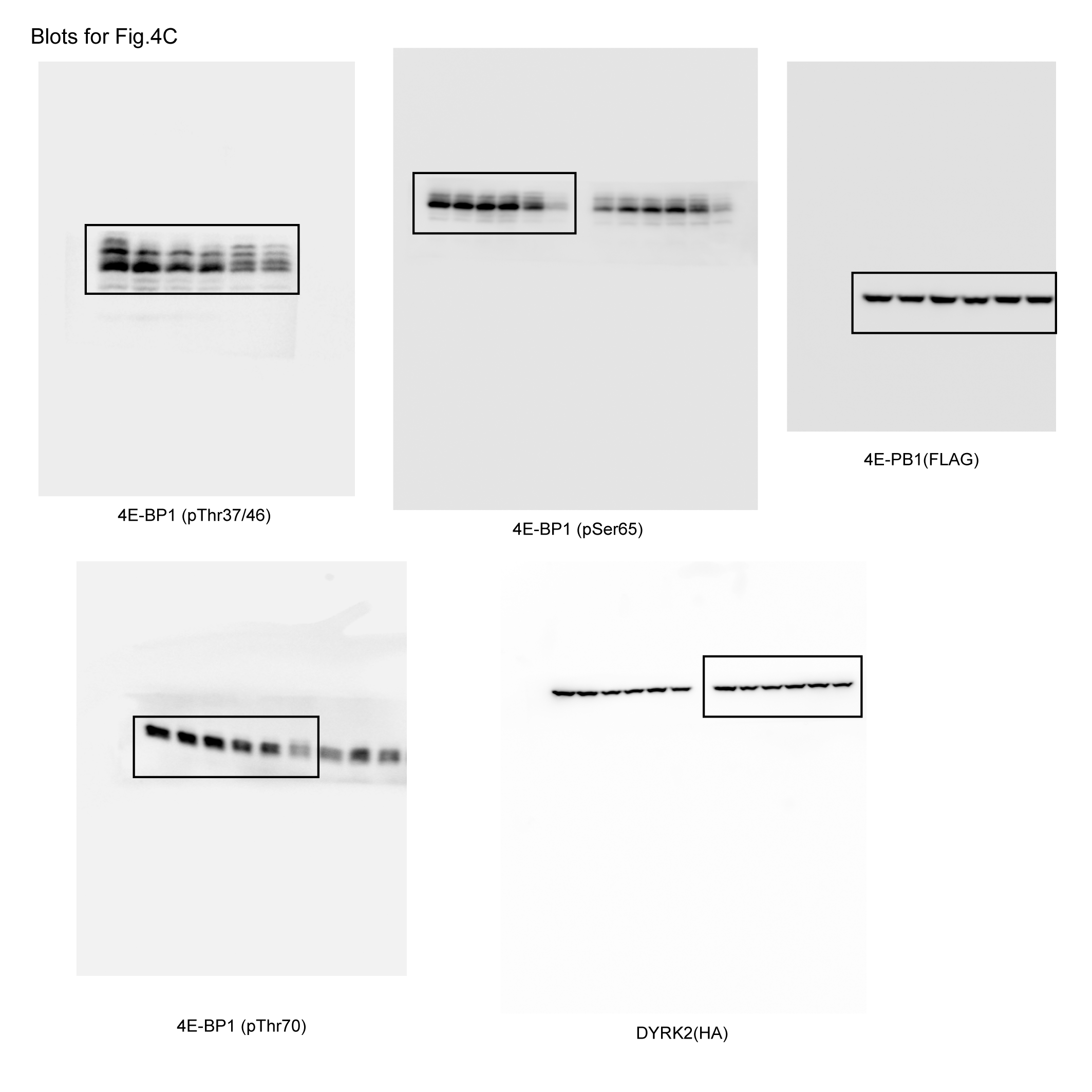

Supplement: Figure 4—source data 2. [file elife-77696-fig4-data2.zip › Figure 4-source data 2/Figure4C-sourcedata/Uncropped_Labeled_Gels_Fig4C.tif]

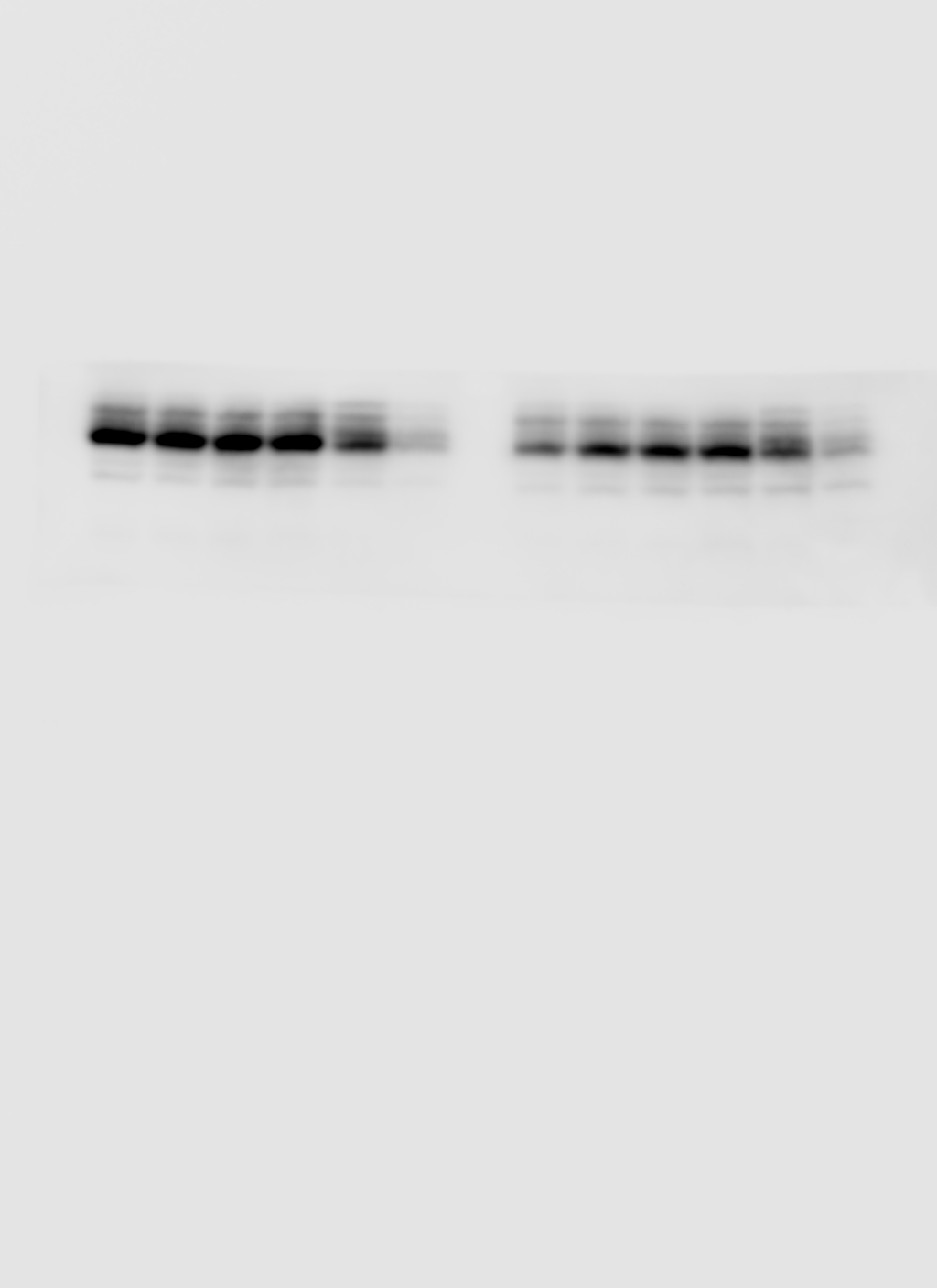

Supplement: Figure 4—source data 2. [file elife-77696-fig4-data2.zip › Figure 4-source data 2/Figure4C-sourcedata/4EBP1-pS65WB_sourcedata.tif]

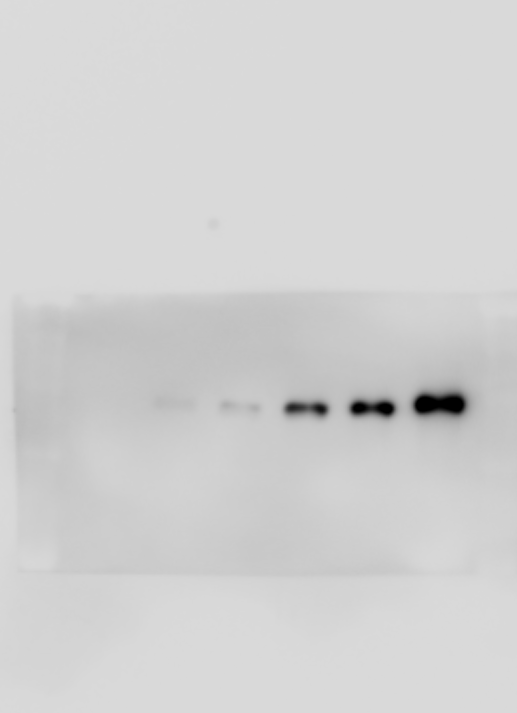

Supplement: Figure 4—source data 2. [file elife-77696-fig4-data2.zip › Figure 4-source data 2/Figure4D-sourcedata/4EBP1-pT70WB_sourcedata.tif]

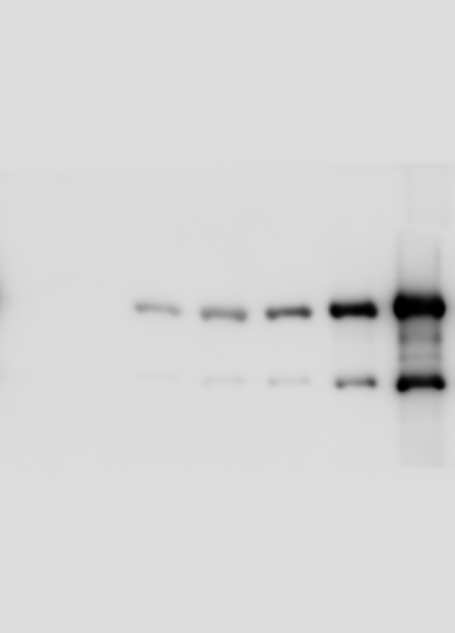

Supplement: Figure 4—source data 2. [file elife-77696-fig4-data2.zip › Figure 4-source data 2/Figure4D-sourcedata/4EBP1-pT37:46WB_sourcedata.tif]

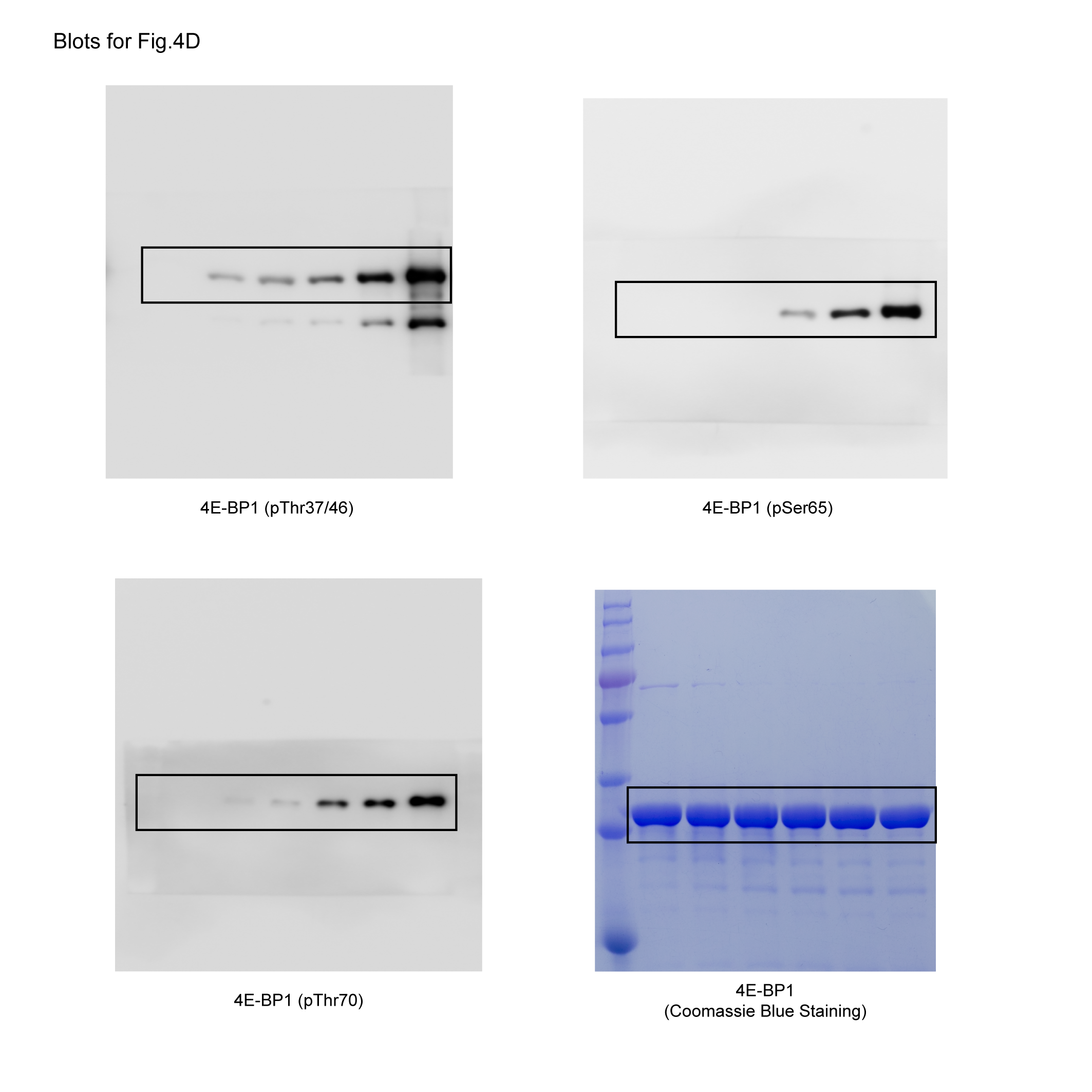

Supplement: Figure 4—source data 2. [file elife-77696-fig4-data2.zip › Figure 4-source data 2/Figure4D-sourcedata/Uncropped_Labeled_Gels_Fig4D.tif]

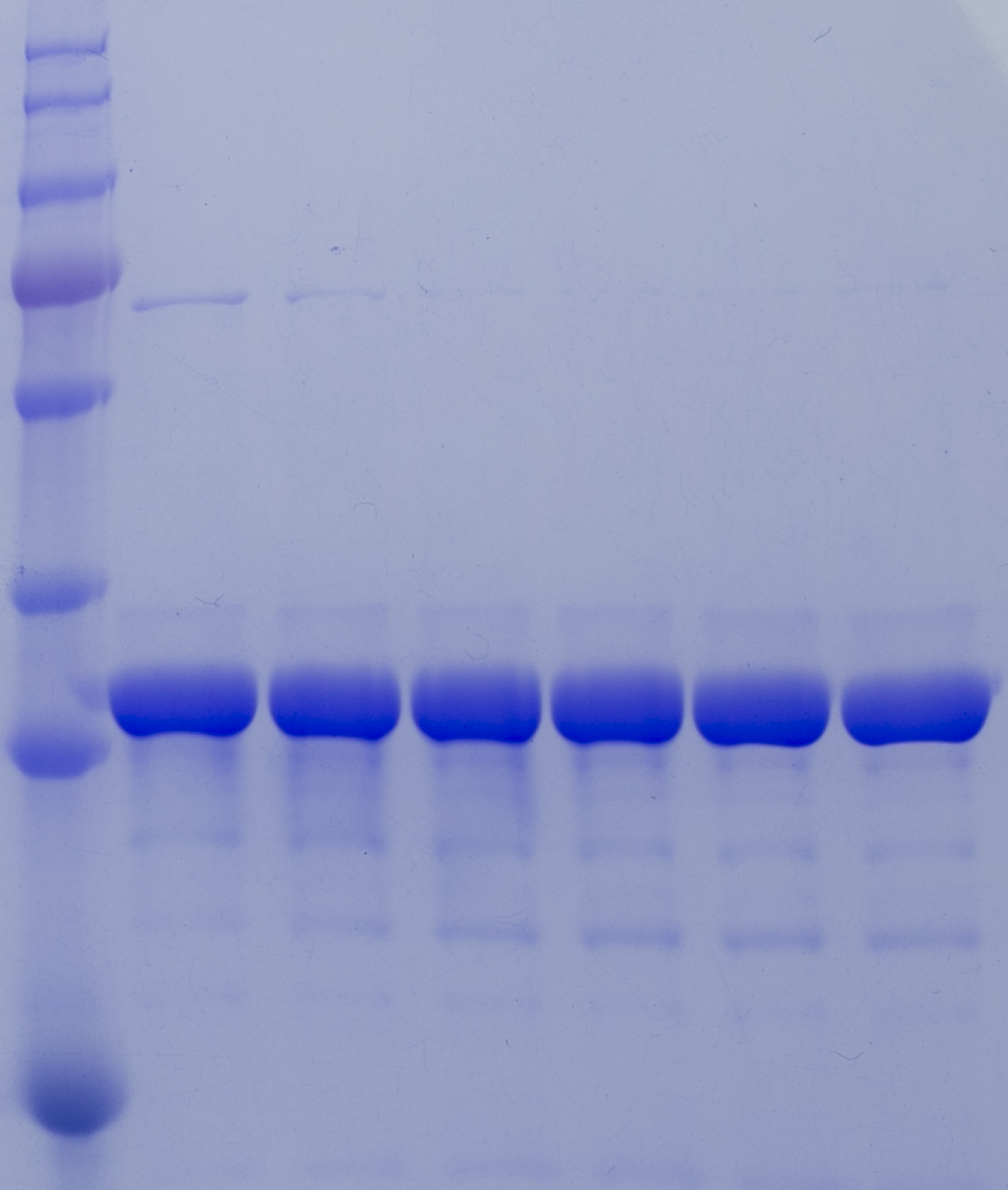

Supplement: Figure 4—source data 2. [file elife-77696-fig4-data2.zip › Figure 4-source data 2/Figure4D-sourcedata/4EBP1-GST-Coomassie Blue Staining.tif]

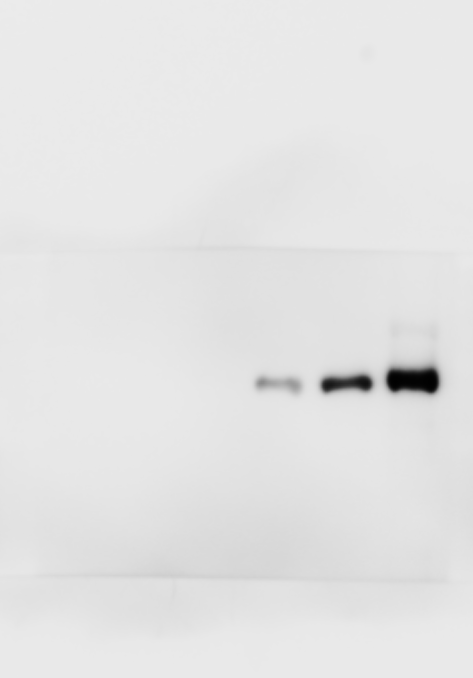

Supplement: Figure 4—source data 2. [file elife-77696-fig4-data2.zip › Figure 4-source data 2/Figure4D-sourcedata/4EBP1-pS65WB_sourcedata.tif]

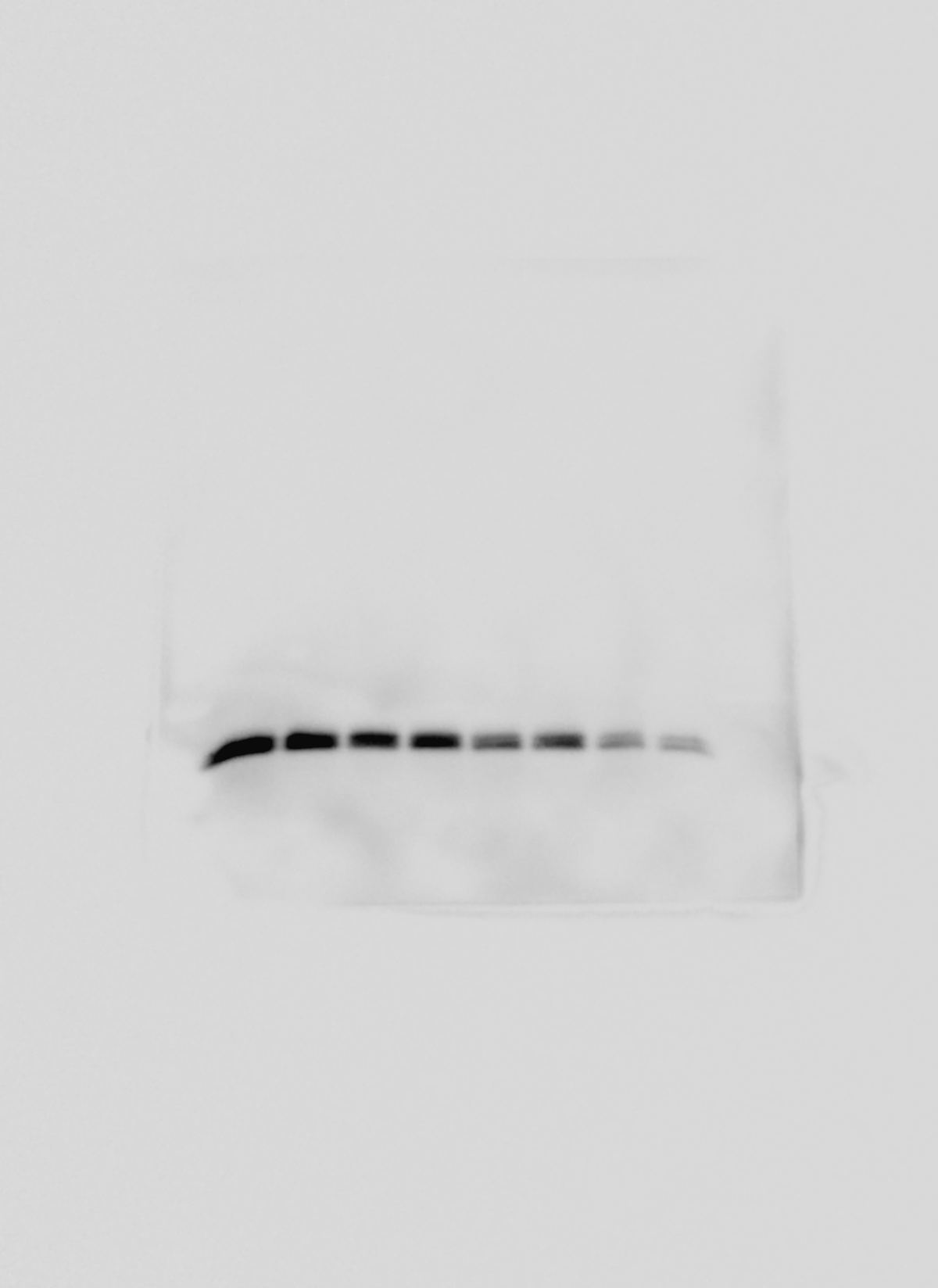

Supplement: Figure 4—source data 3. [file elife-77696-fig4-data3.zip › Figure 4-source data 3/Figure4F-sourcedata/4EBP1-pT70WB_sourcedata.tif]

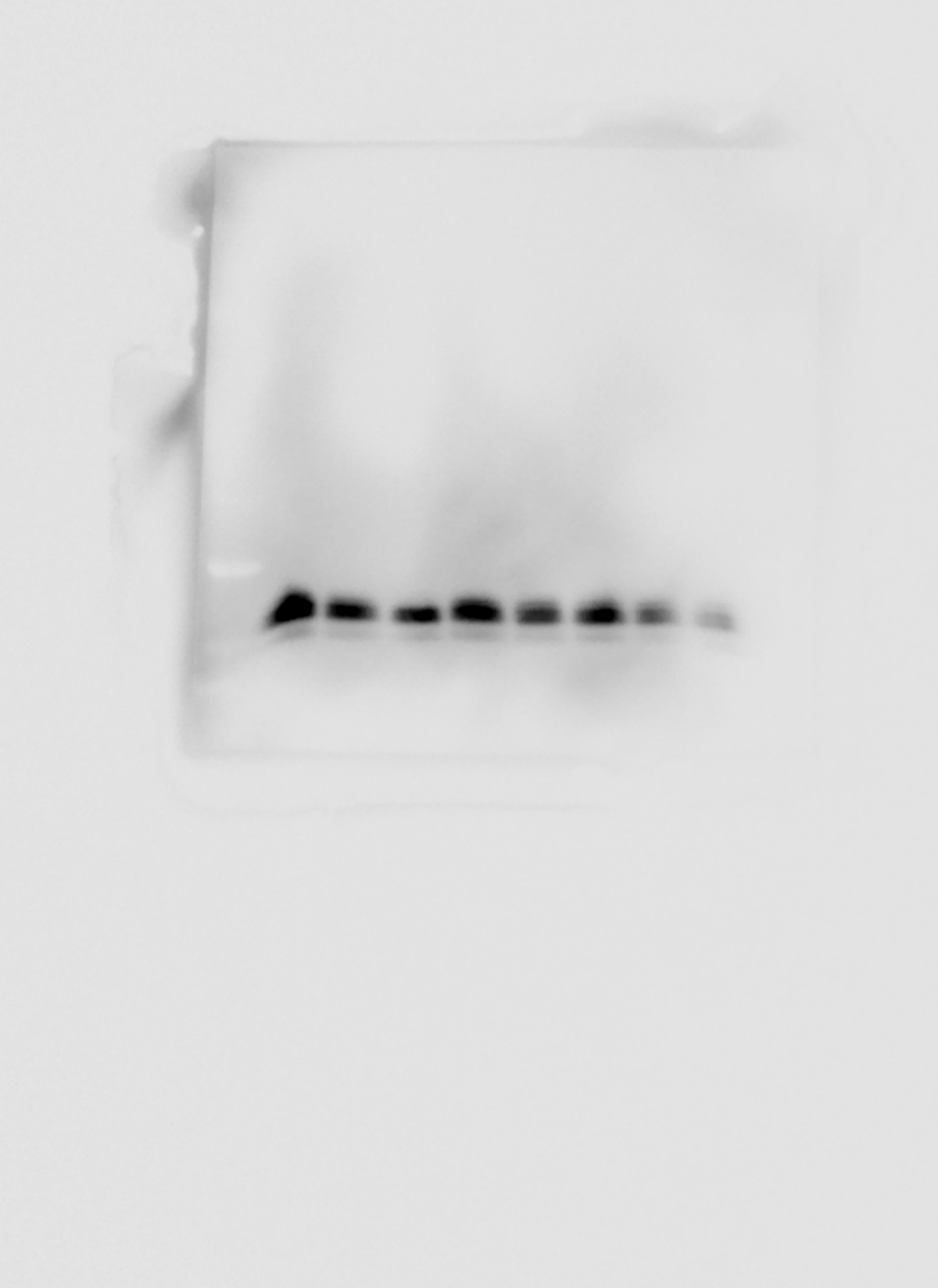

Supplement: Figure 4—source data 3. [file elife-77696-fig4-data3.zip › Figure 4-source data 3/Figure4F-sourcedata/4EBP1-pT37:46WB_sourcedata.tif]

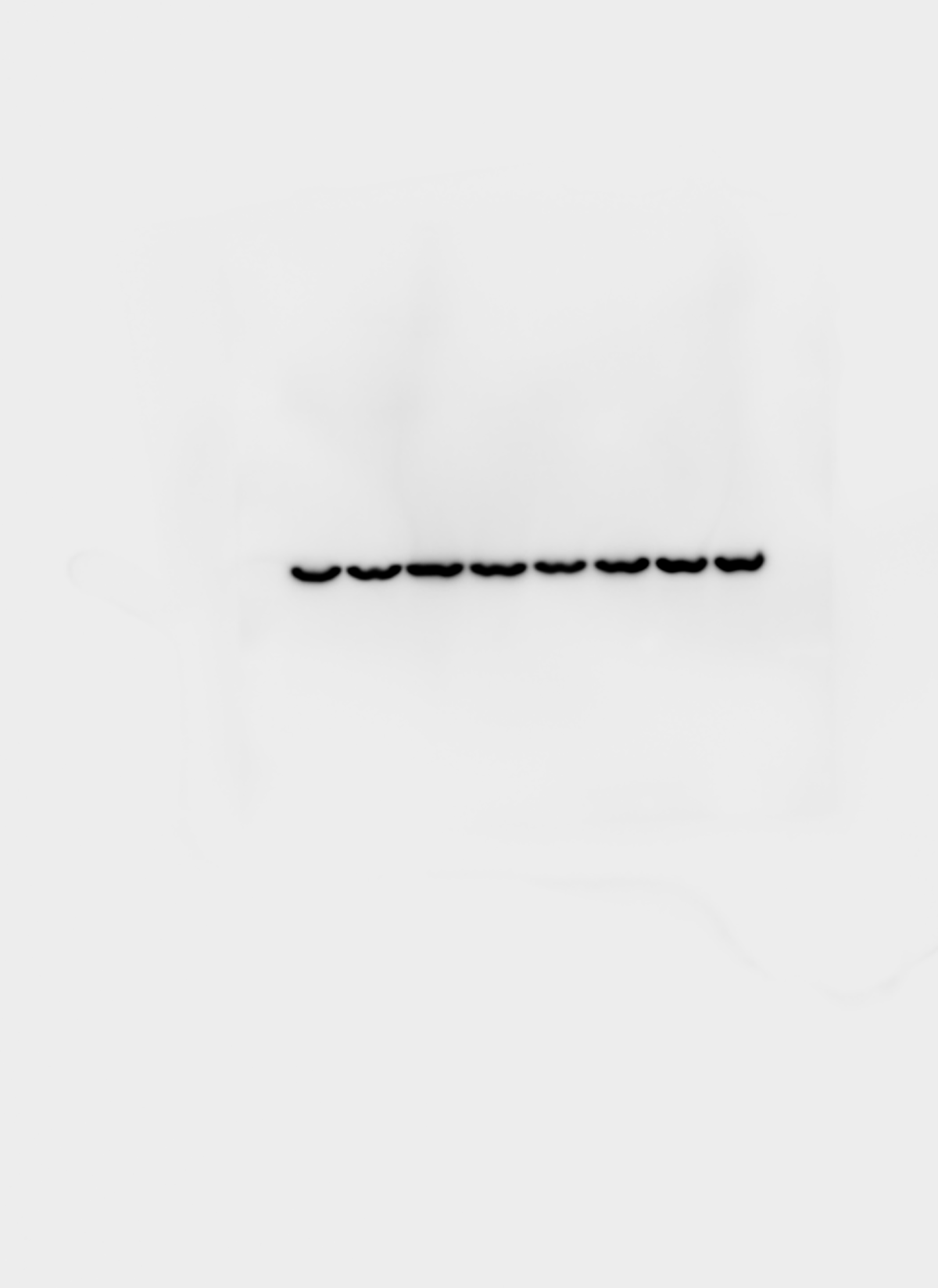

Supplement: Figure 4—source data 3. [file elife-77696-fig4-data3.zip › Figure 4-source data 3/Figure4F-sourcedata/GAPDHWB_sourcedata.tif]

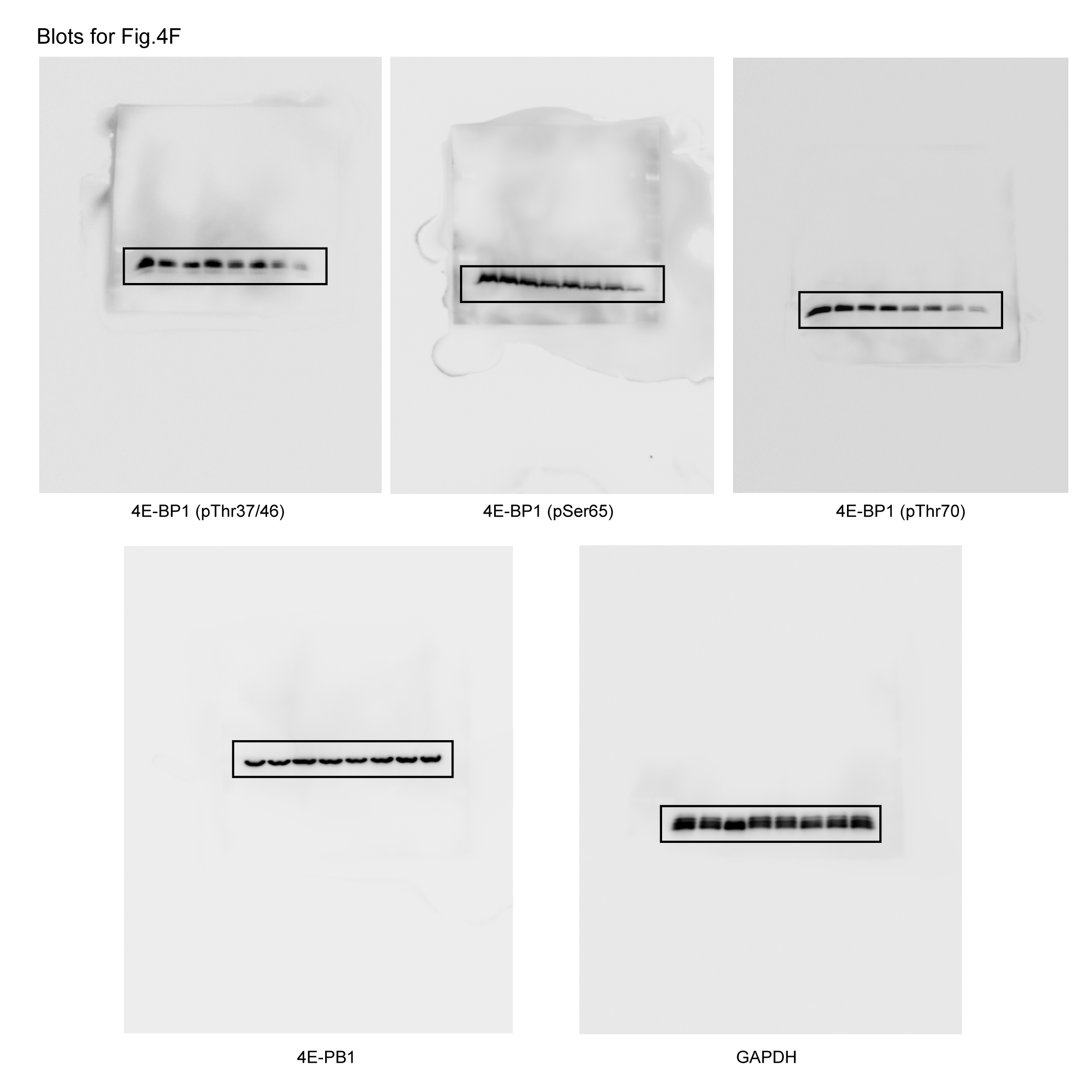

Supplement: Figure 4—source data 3. [file elife-77696-fig4-data3.zip › Figure 4-source data 3/Figure4F-sourcedata/Uncropped_Labeled_Gels_Fig4F.tif]

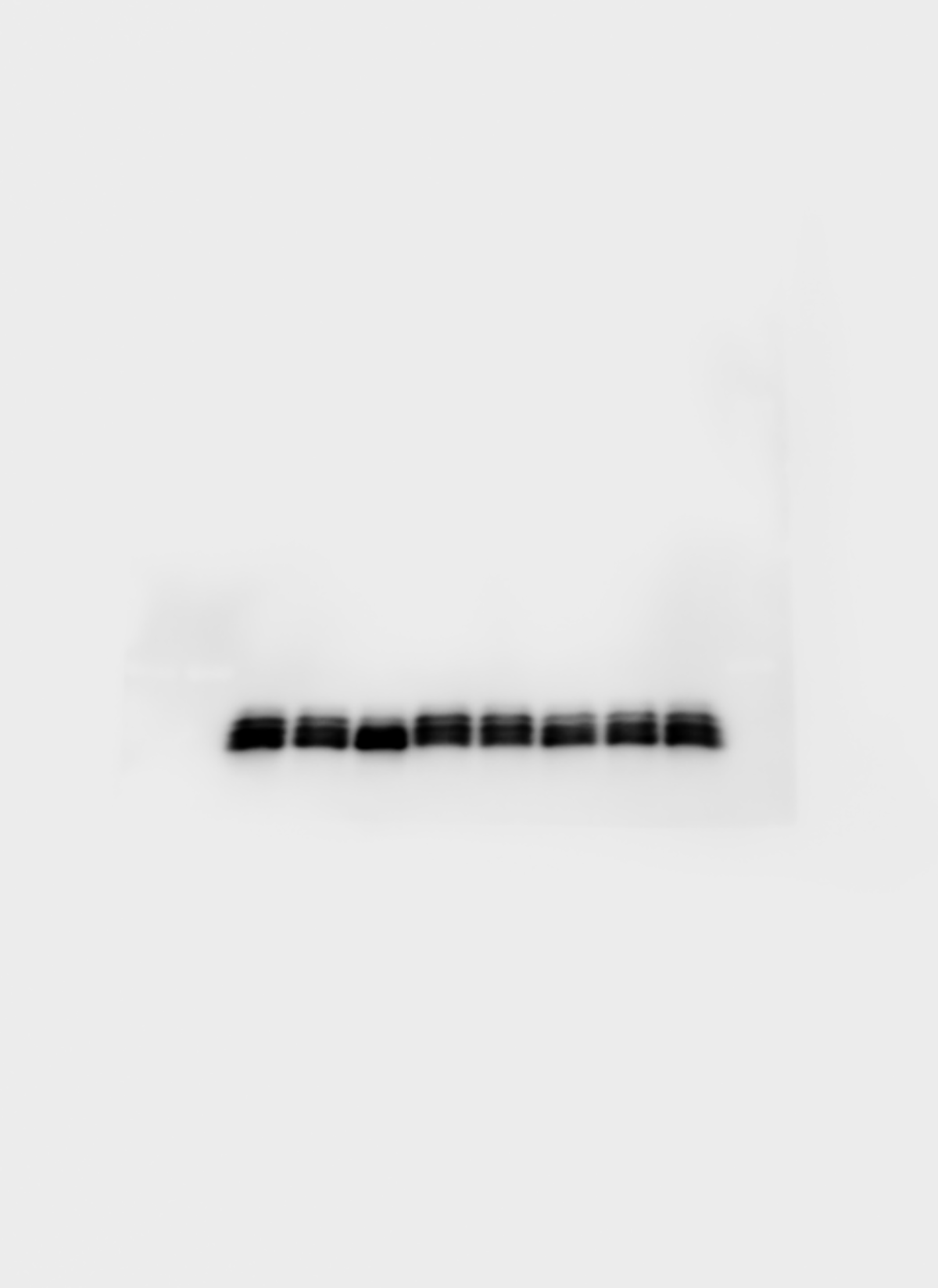

Supplement: Figure 4—source data 3. [file elife-77696-fig4-data3.zip › Figure 4-source data 3/Figure4F-sourcedata/4EBP1WB_sourcedata.tif]

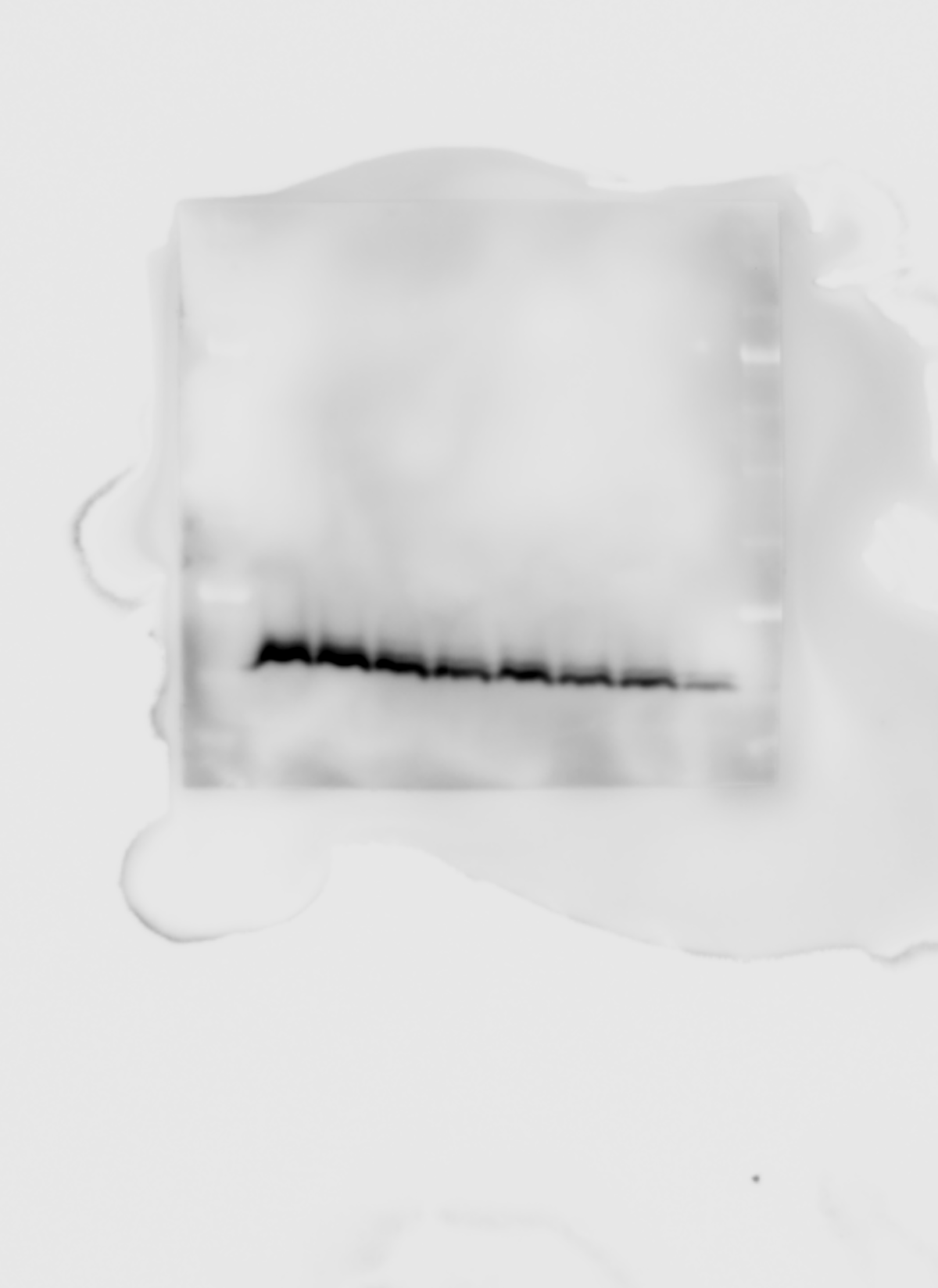

Supplement: Figure 4—source data 3. [file elife-77696-fig4-data3.zip › Figure 4-source data 3/Figure4F-sourcedata/4EBP1-pS65WB_sourcedata.tif]

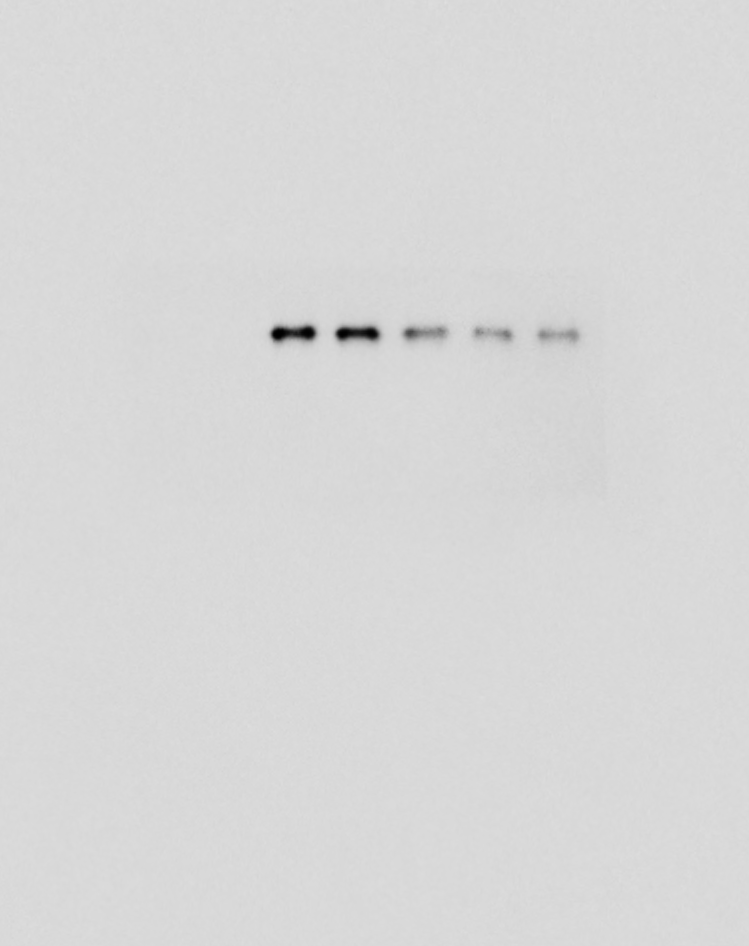

Supplement: Figure 4—source data 3. [file elife-77696-fig4-data3.zip › Figure 4-source data 3/Figure4E-sourcedata/4EBP1-pT70WB_sourcedata.tif]

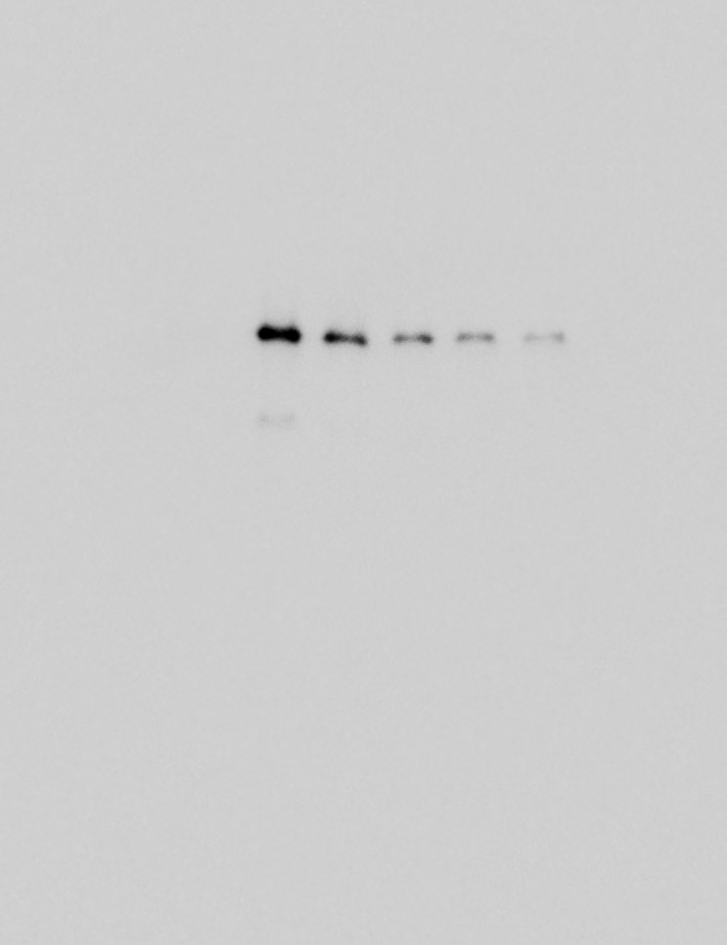

Supplement: Figure 4—source data 3. [file elife-77696-fig4-data3.zip › Figure 4-source data 3/Figure4E-sourcedata/4EBP1-pT37:46WB_sourcedata.tif]

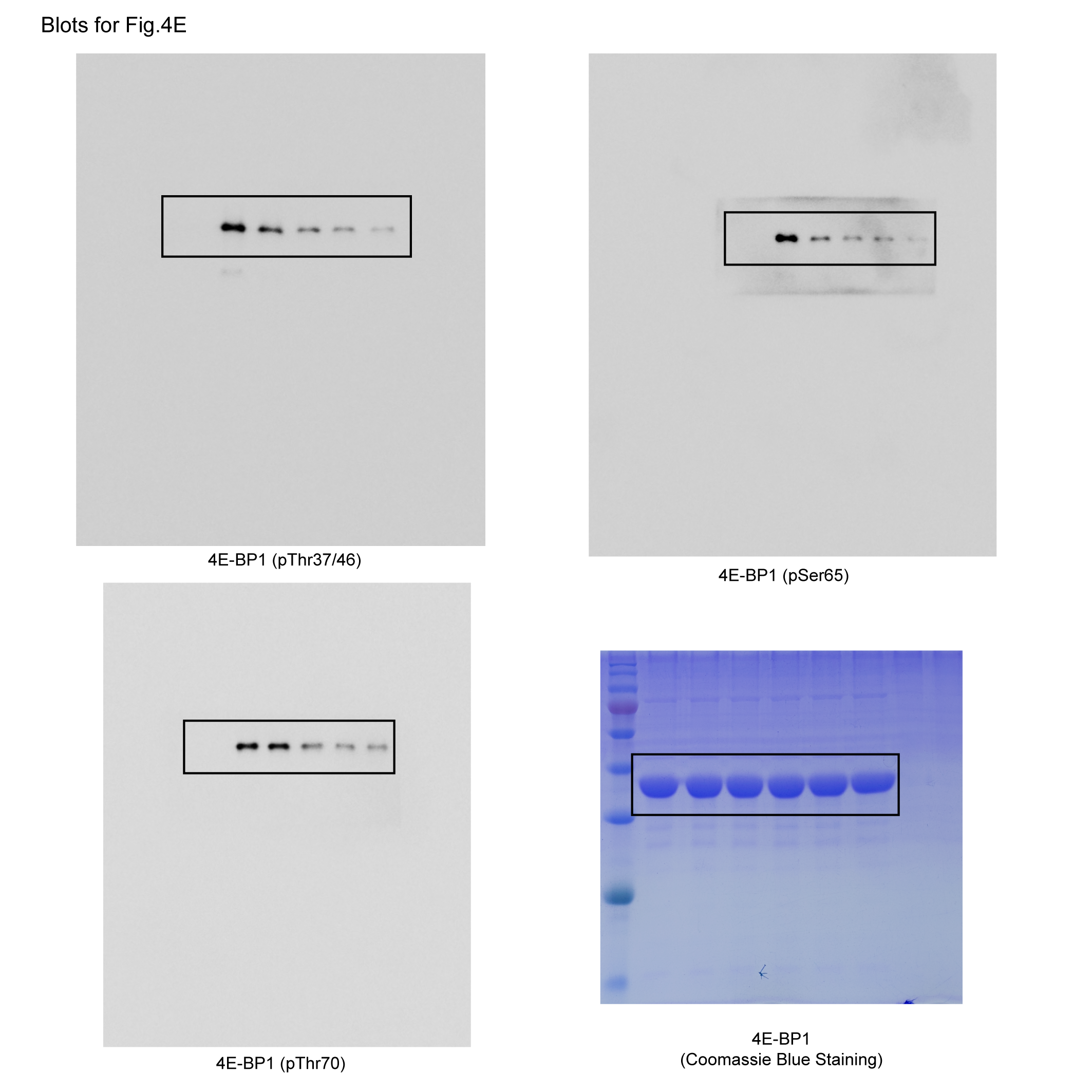

Supplement: Figure 4—source data 3. [file elife-77696-fig4-data3.zip › Figure 4-source data 3/Figure4E-sourcedata/Uncropped_Labeled_Gels_Fig4E.tif]

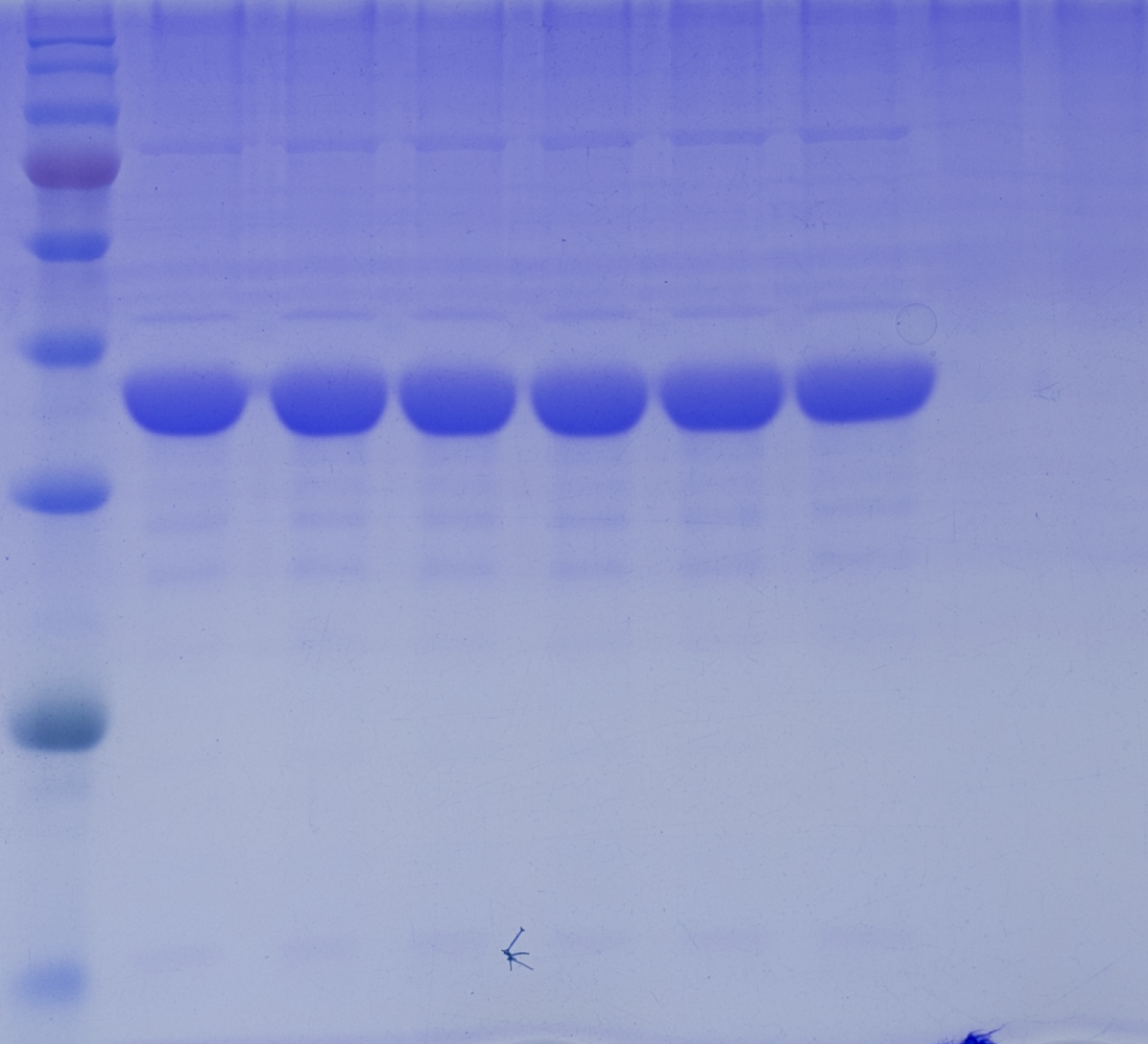

Supplement: Figure 4—source data 3. [file elife-77696-fig4-data3.zip › Figure 4-source data 3/Figure4E-sourcedata/4EBP1-GST-Coomassie Blue Staining.tif]

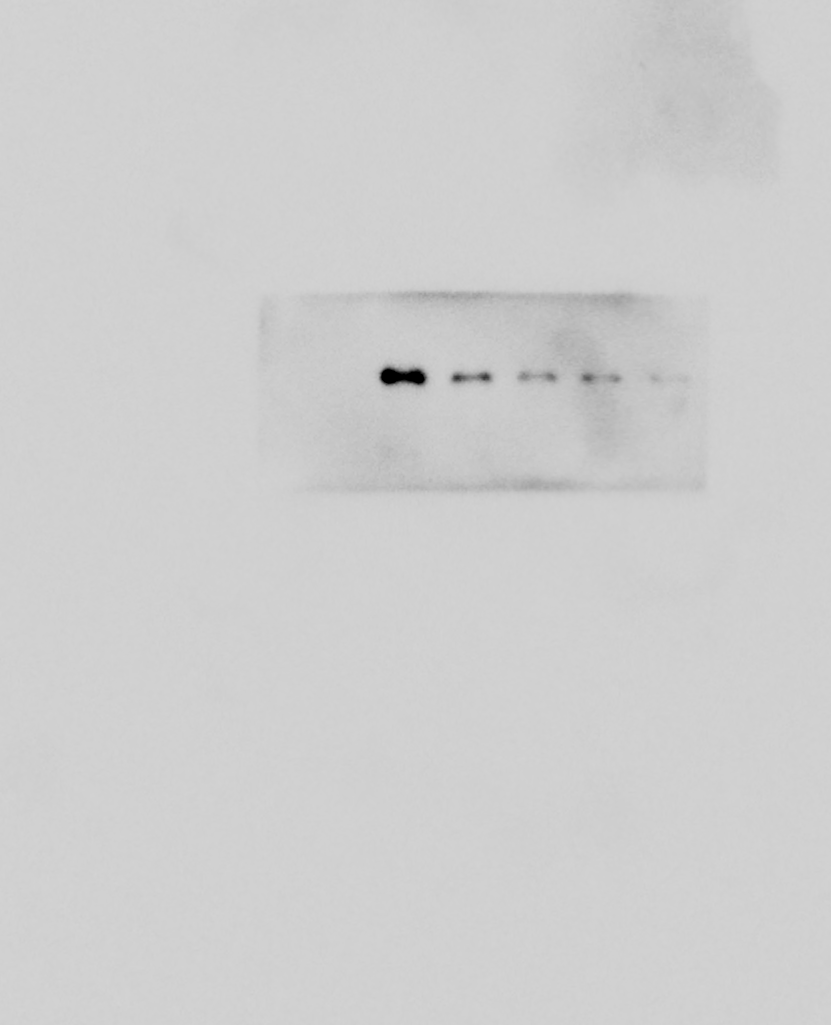

Supplement: Figure 4—source data 3. [file elife-77696-fig4-data3.zip › Figure 4-source data 3/Figure4E-sourcedata/4EBP1-pS65WB_sourcedata.tif]

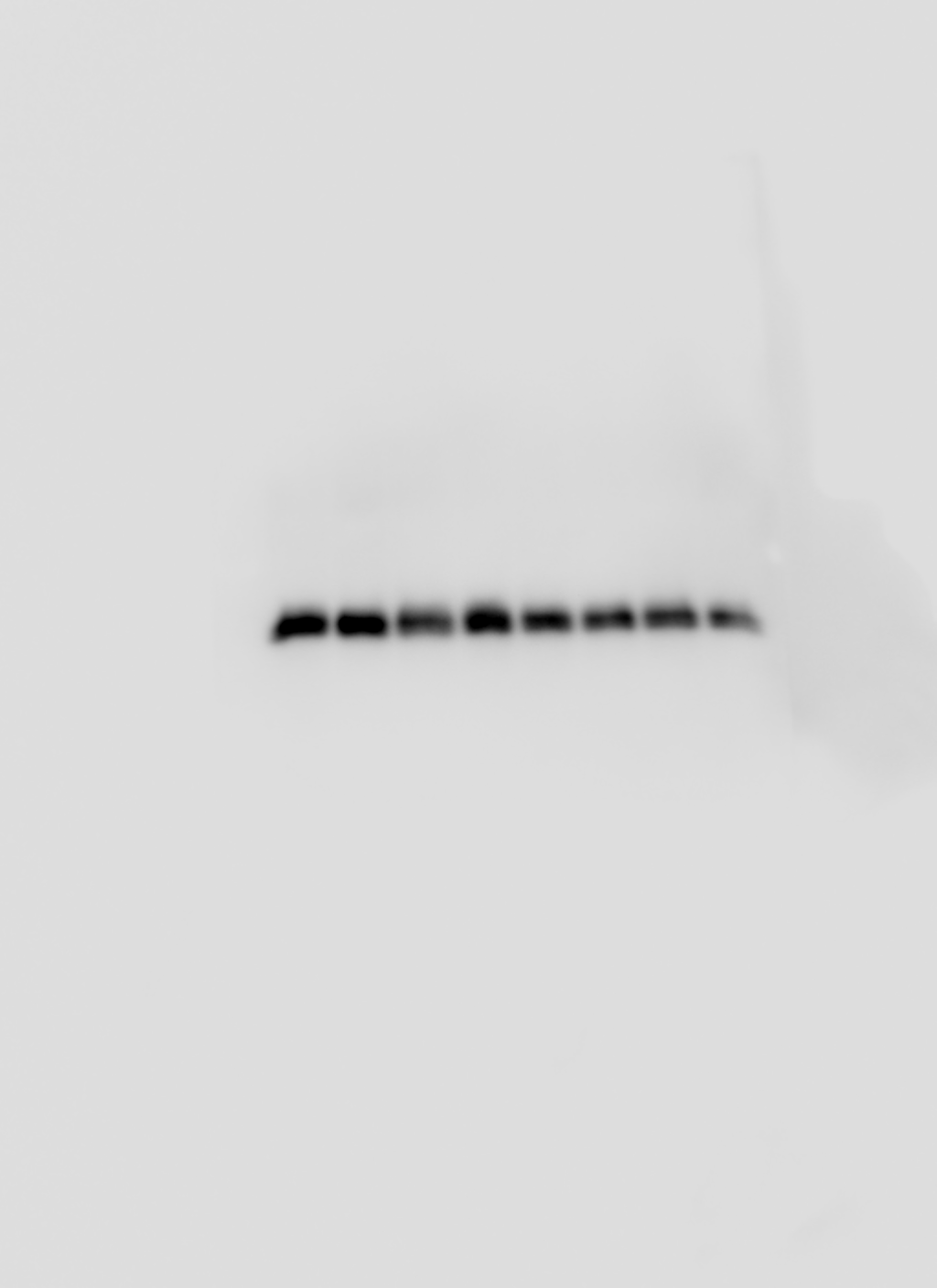

Supplement: Figure 4—source data 4. [file elife-77696-fig4-data4.zip › Figure 4-source data 4/Figure4G-sourcedata/4EBP1-pT70WB_sourcedata.tif]

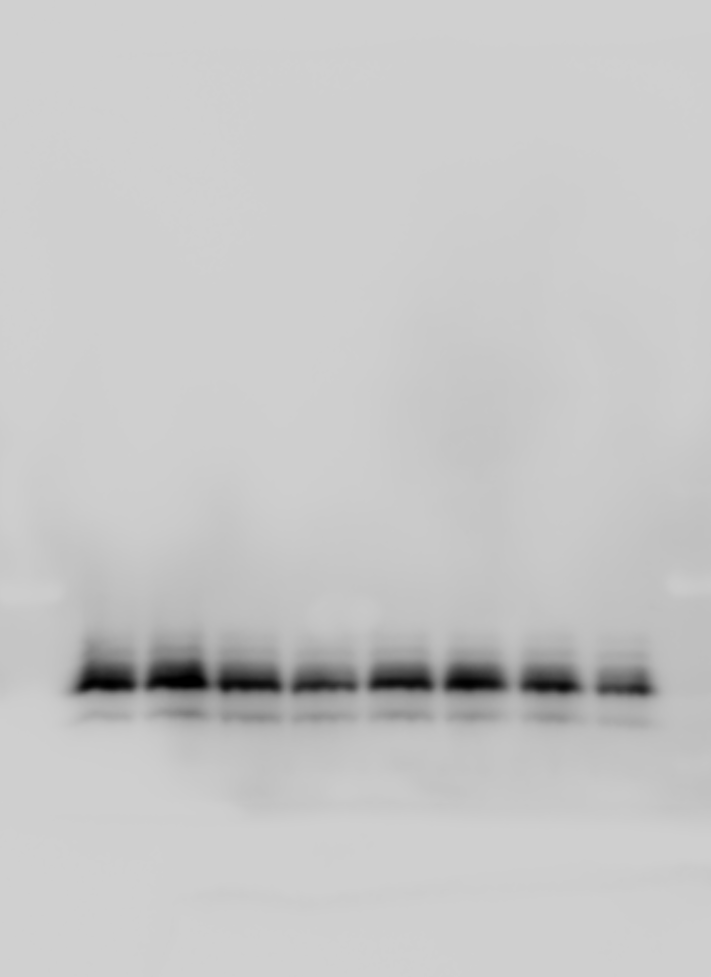

Supplement: Figure 4—source data 4. [file elife-77696-fig4-data4.zip › Figure 4-source data 4/Figure4G-sourcedata/4EBP1-pT37:46WB_sourcedata.tif]

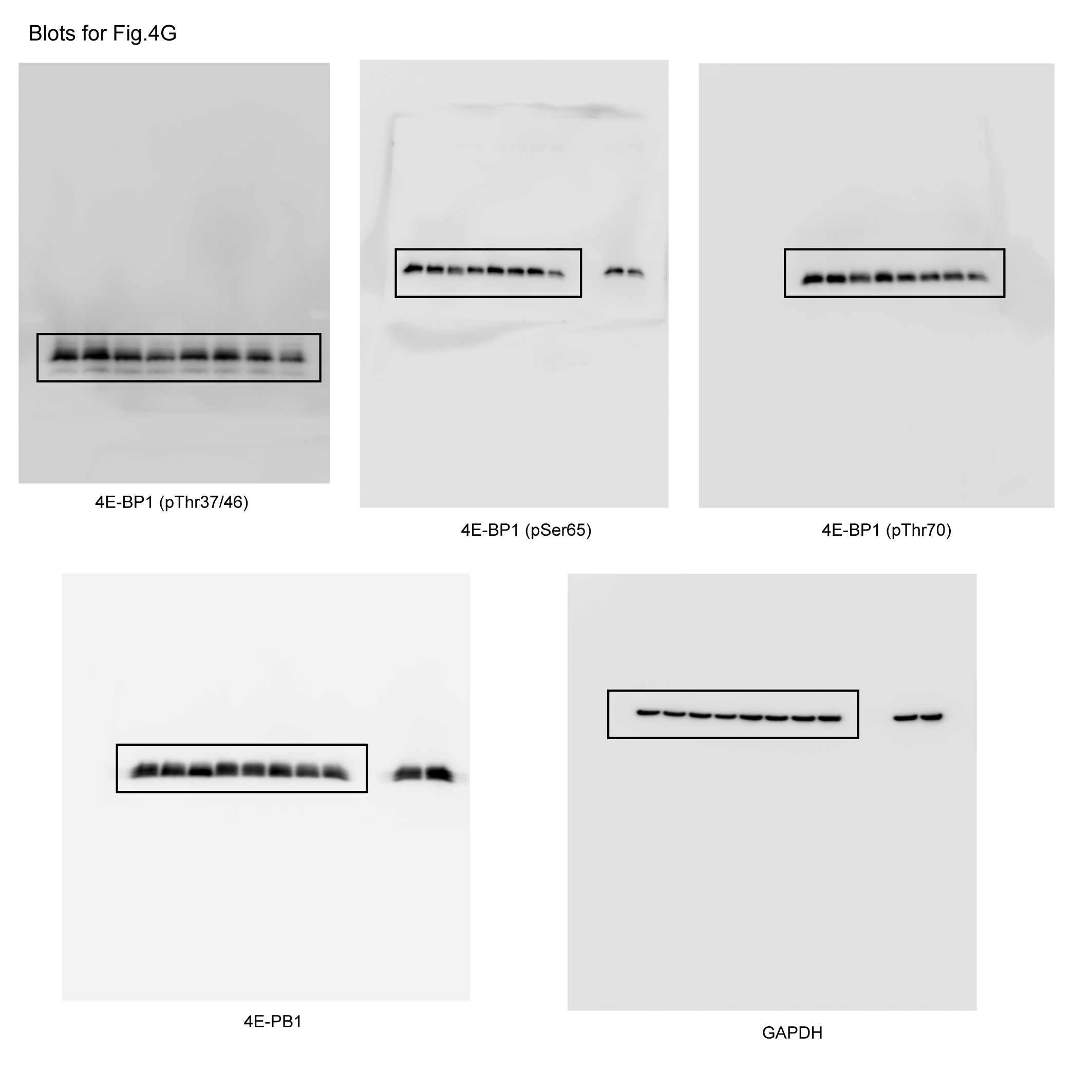

Supplement: Figure 4—source data 4. [file elife-77696-fig4-data4.zip › Figure 4-source data 4/Figure4G-sourcedata/Uncropped_Labeled_Gels_Fig4G.tif]

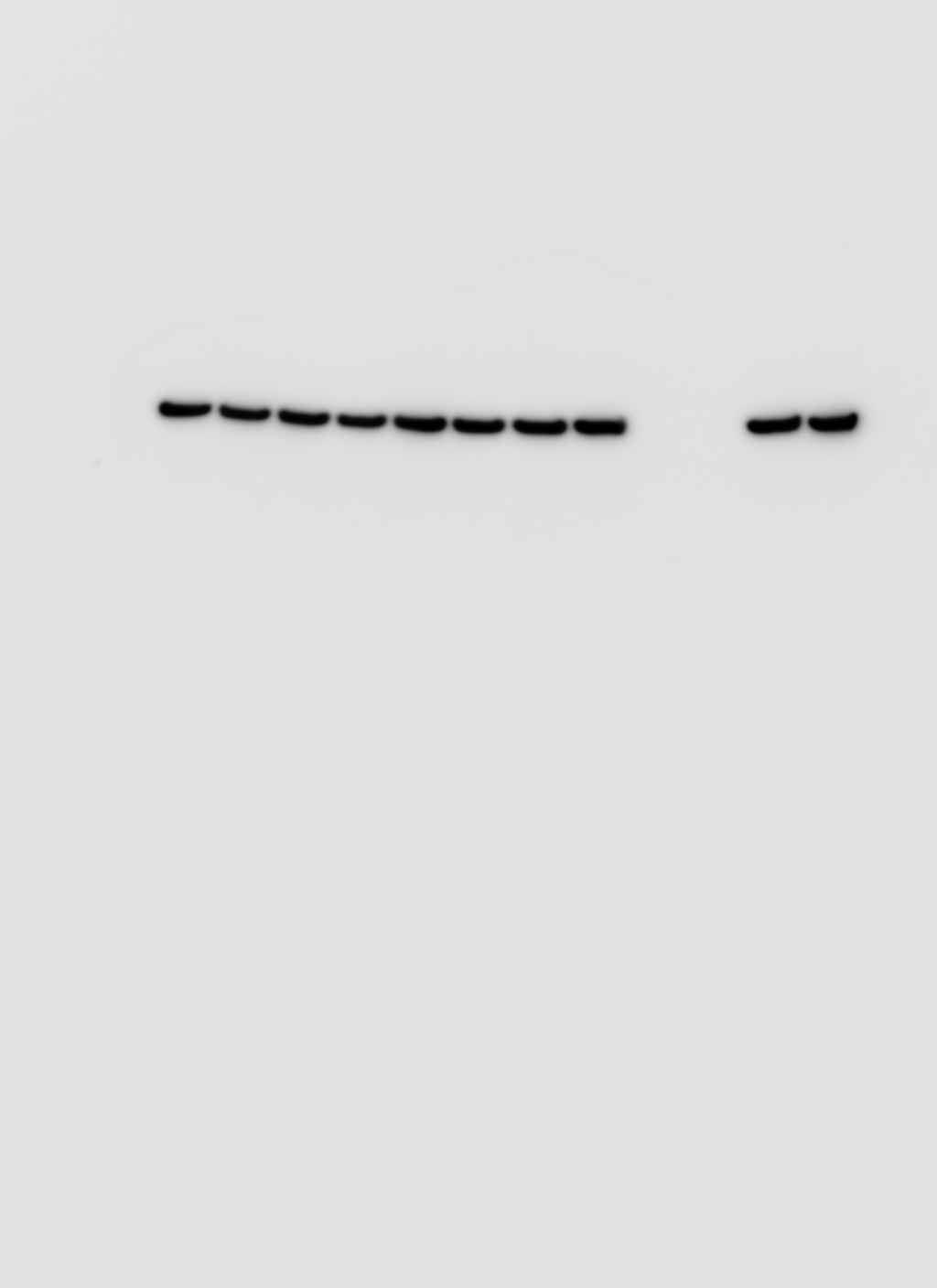

Supplement: Figure 4—source data 4. [file elife-77696-fig4-data4.zip › Figure 4-source data 4/Figure4G-sourcedata/GAPDHWB_sourcedata.tif]

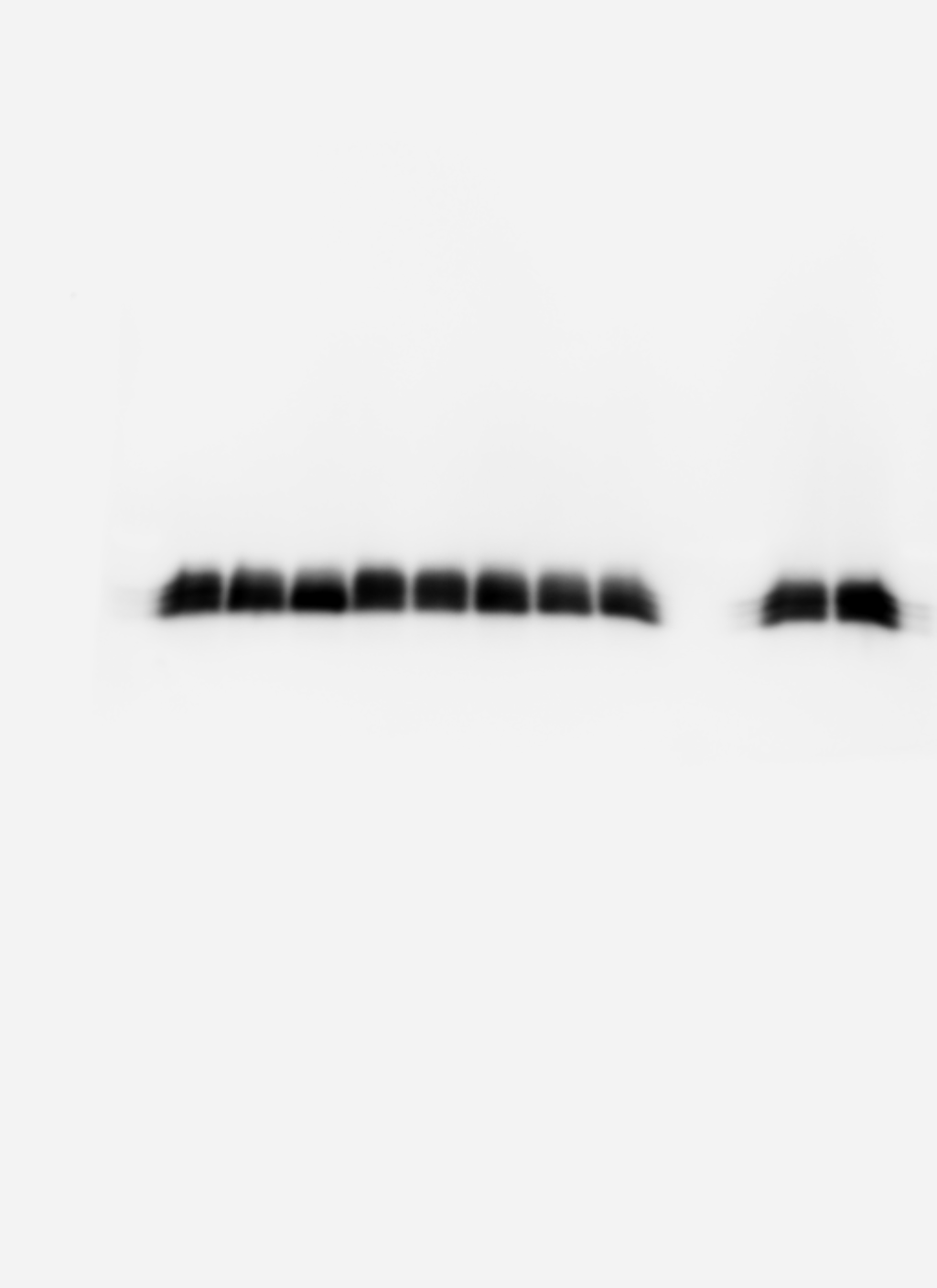

Supplement: Figure 4—source data 4. [file elife-77696-fig4-data4.zip › Figure 4-source data 4/Figure4G-sourcedata/4EBP1WB_sourcedata.tif]

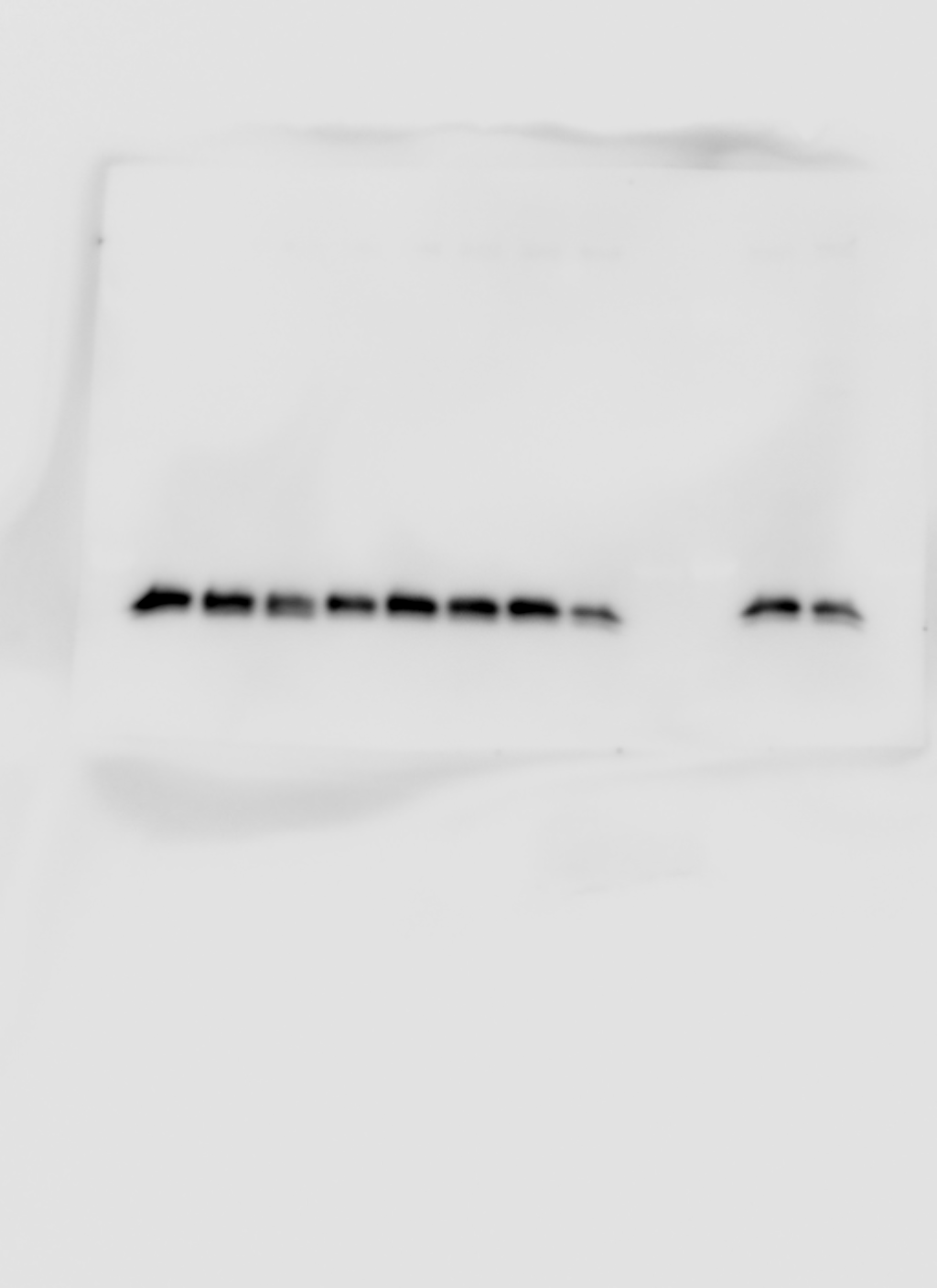

Supplement: Figure 4—source data 4. [file elife-77696-fig4-data4.zip › Figure 4-source data 4/Figure4G-sourcedata/4EBP1-pS65WB_sourcedata.tif]

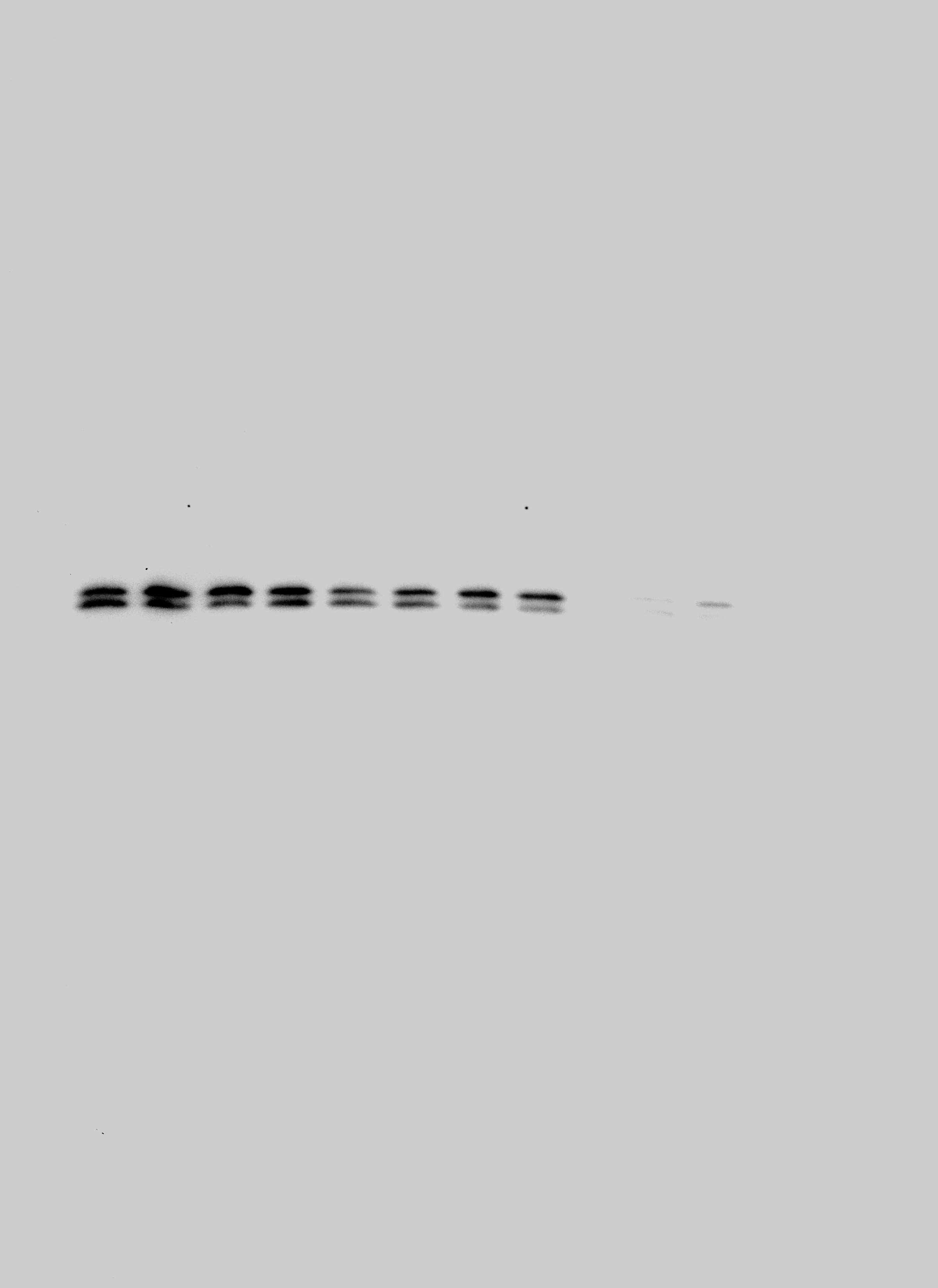

Supplement: Figure 4—source data 4. [file elife-77696-fig4-data4.zip › Figure 4-source data 4/Figure4H-sourcedata/4EBP1-pT70WB_sourcedata.tif]

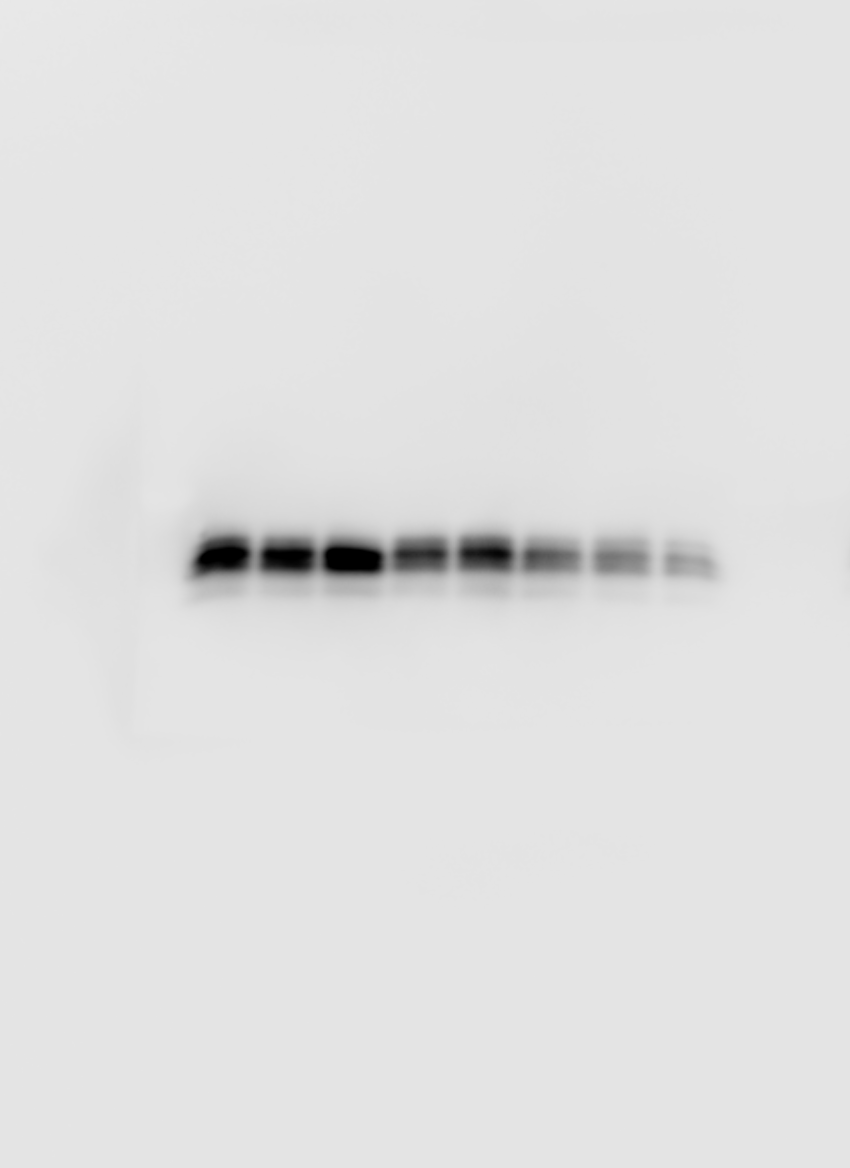

Supplement: Figure 4—source data 4. [file elife-77696-fig4-data4.zip › Figure 4-source data 4/Figure4H-sourcedata/4EBP1-pT37:46WB_sourcedata.tif]

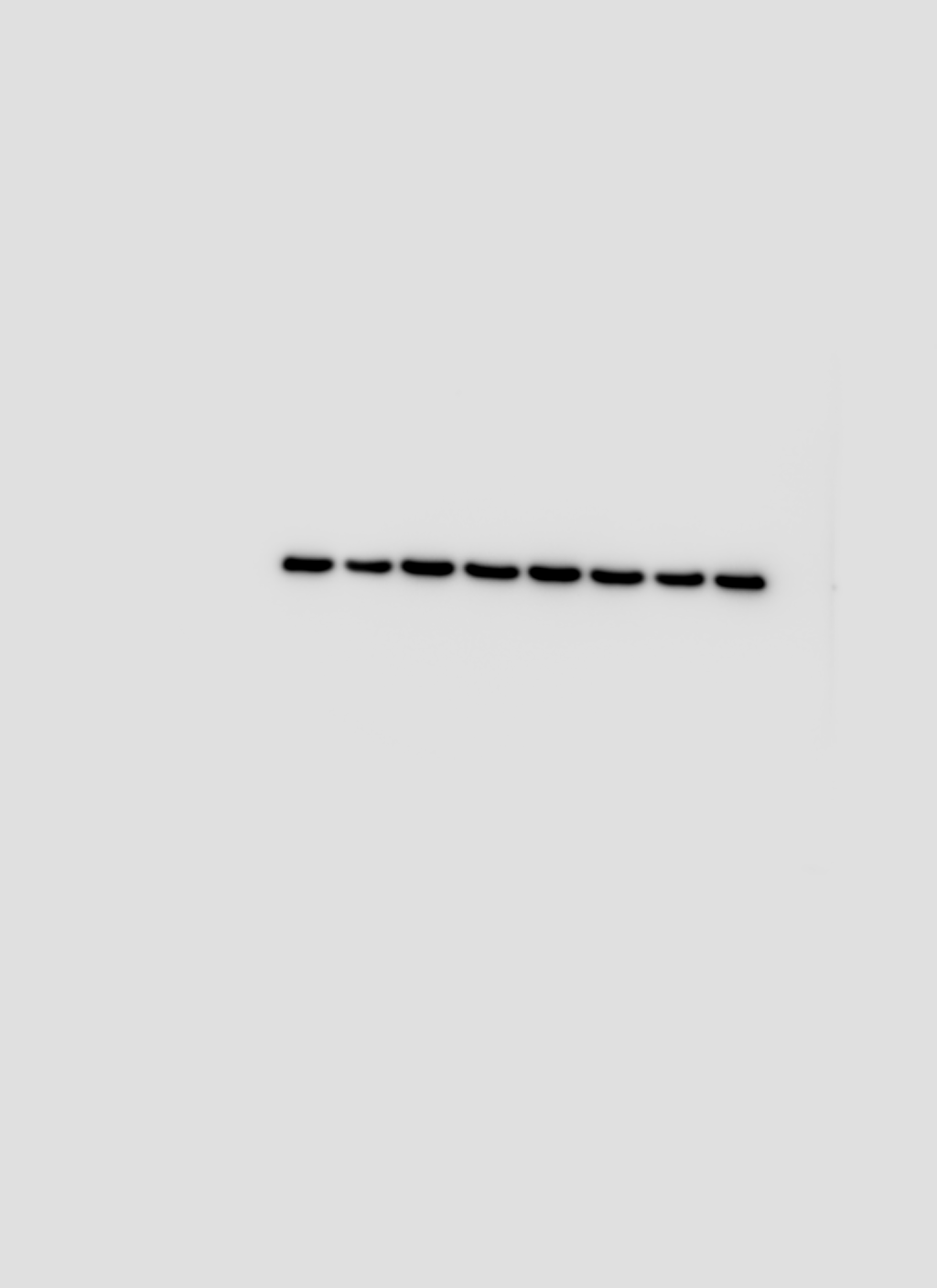

Supplement: Figure 4—source data 4. [file elife-77696-fig4-data4.zip › Figure 4-source data 4/Figure4H-sourcedata/GAPDHWB_sourcedata.tif]

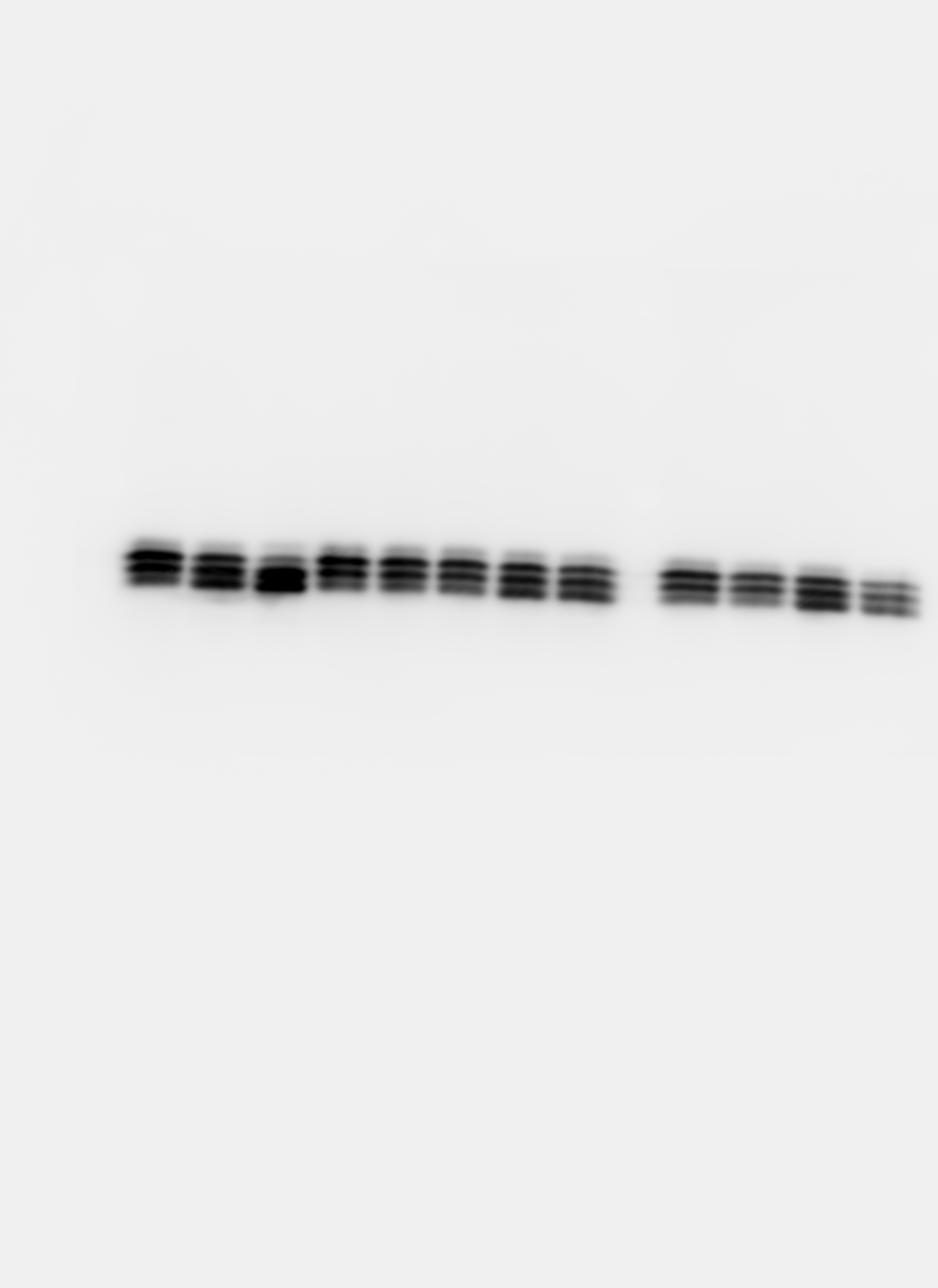

Supplement: Figure 4—source data 4. [file elife-77696-fig4-data4.zip › Figure 4-source data 4/Figure4H-sourcedata/4EBP1WB_sourcedata.tif]

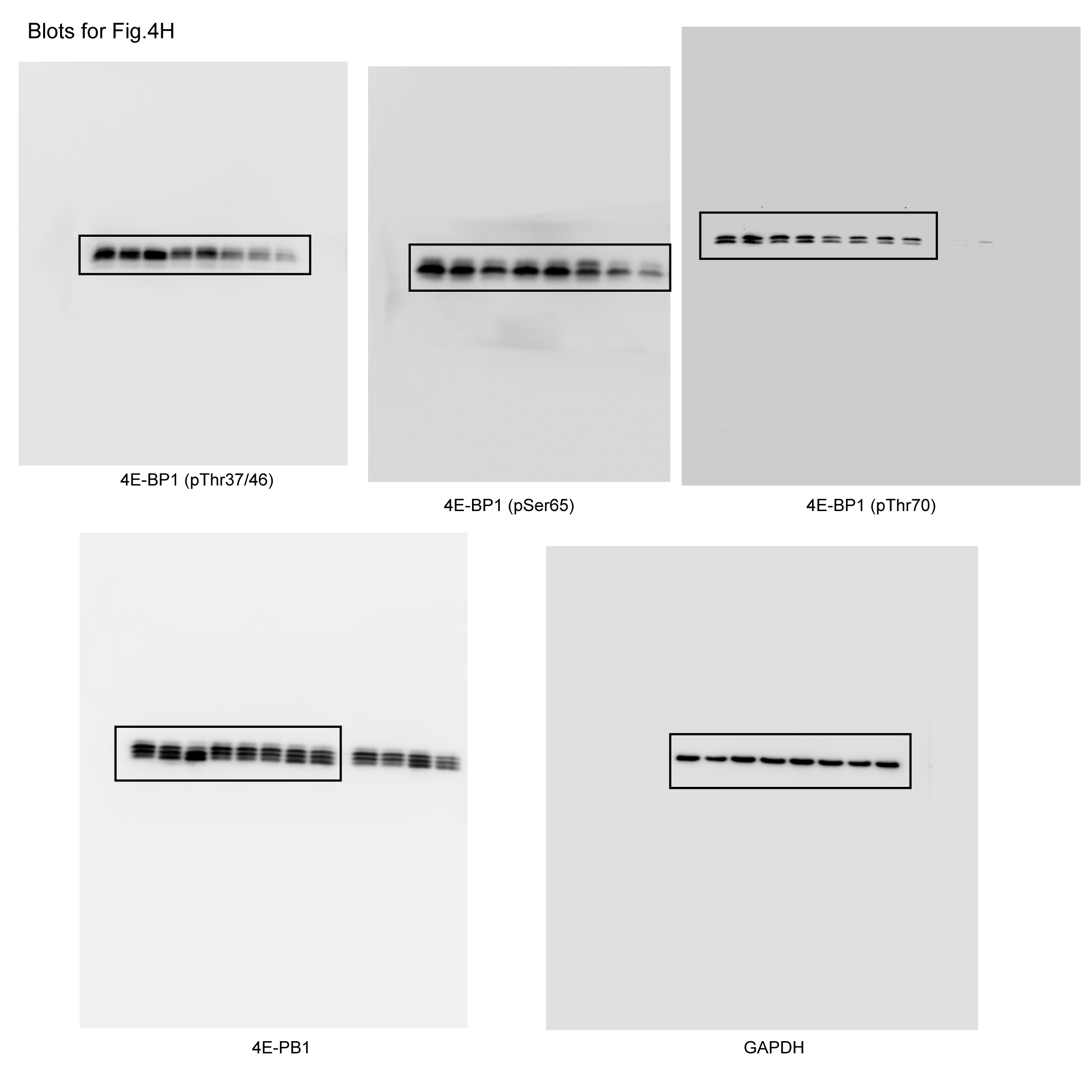

Supplement: Figure 4—source data 4. [file elife-77696-fig4-data4.zip › Figure 4-source data 4/Figure4H-sourcedata/Uncropped_Labeled_Gels_Fig4H.tif]

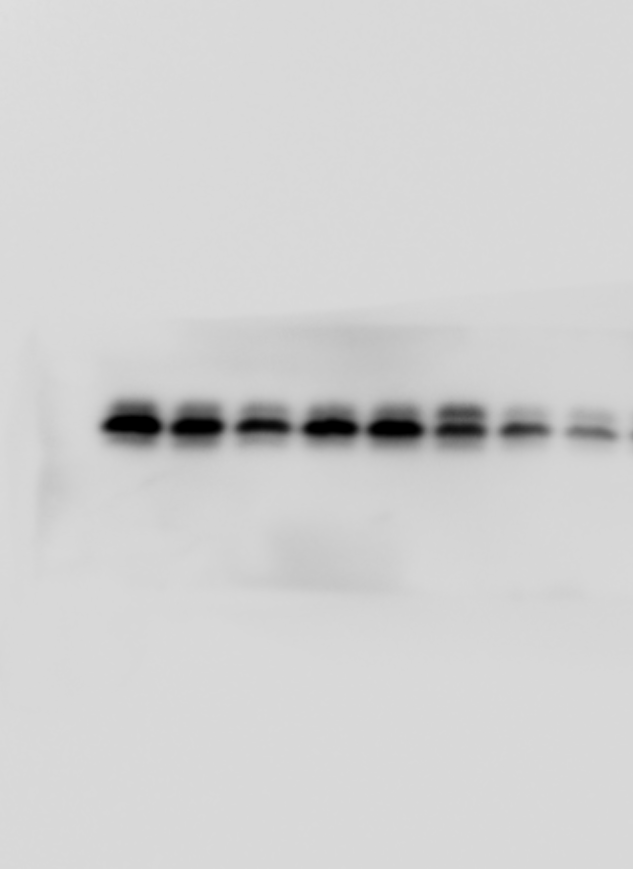

Supplement: Figure 4—source data 4. [file elife-77696-fig4-data4.zip › Figure 4-source data 4/Figure4H-sourcedata/4EBP1-pS65WB_sourcedata.tif]

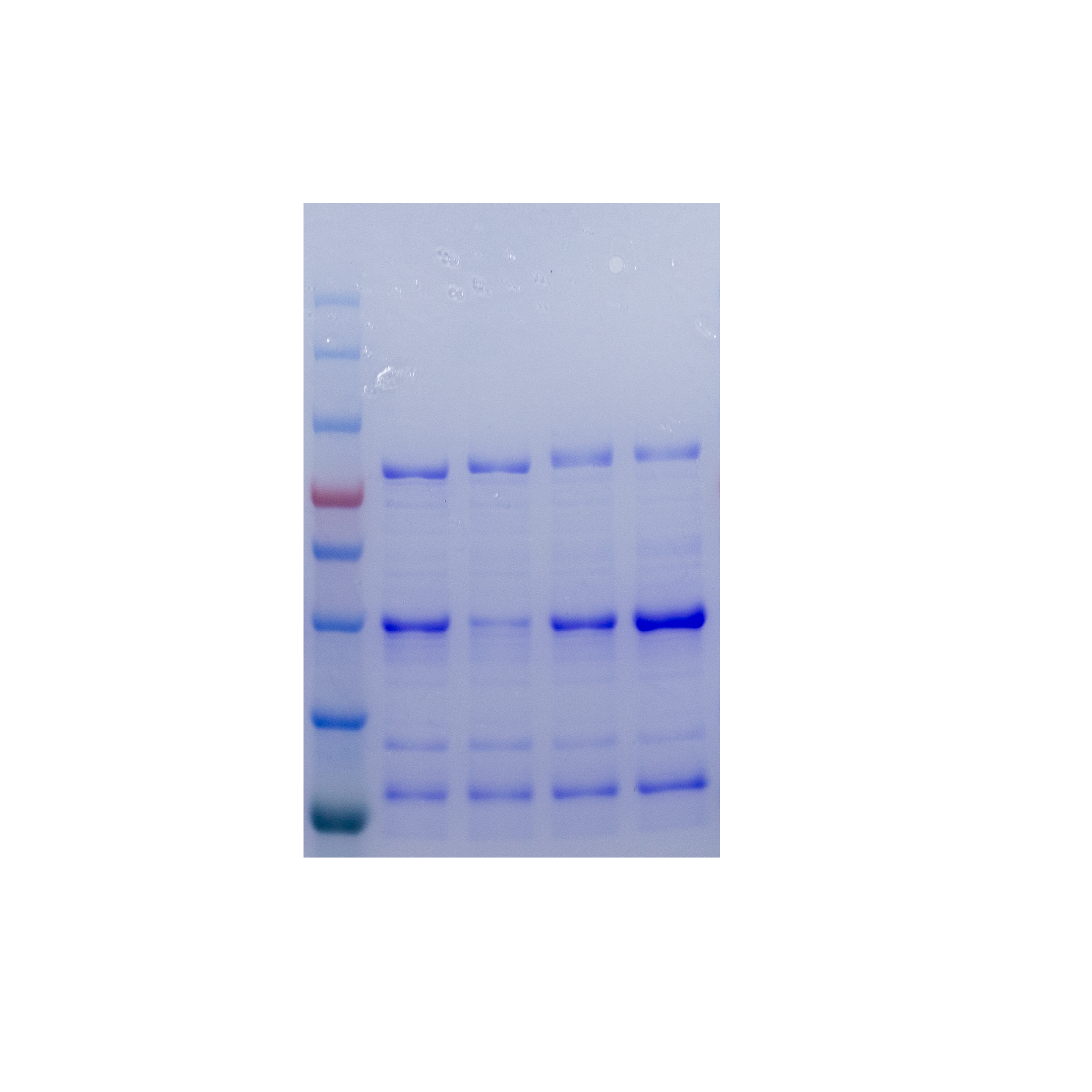

Supplement: Figure 5—source data 1. [file elife-77696-fig5-data1.zip › Figure 5-source data 1/Figure 5-source data 1-in vitro kinase assay_Coomassie Blue Staining.tif]

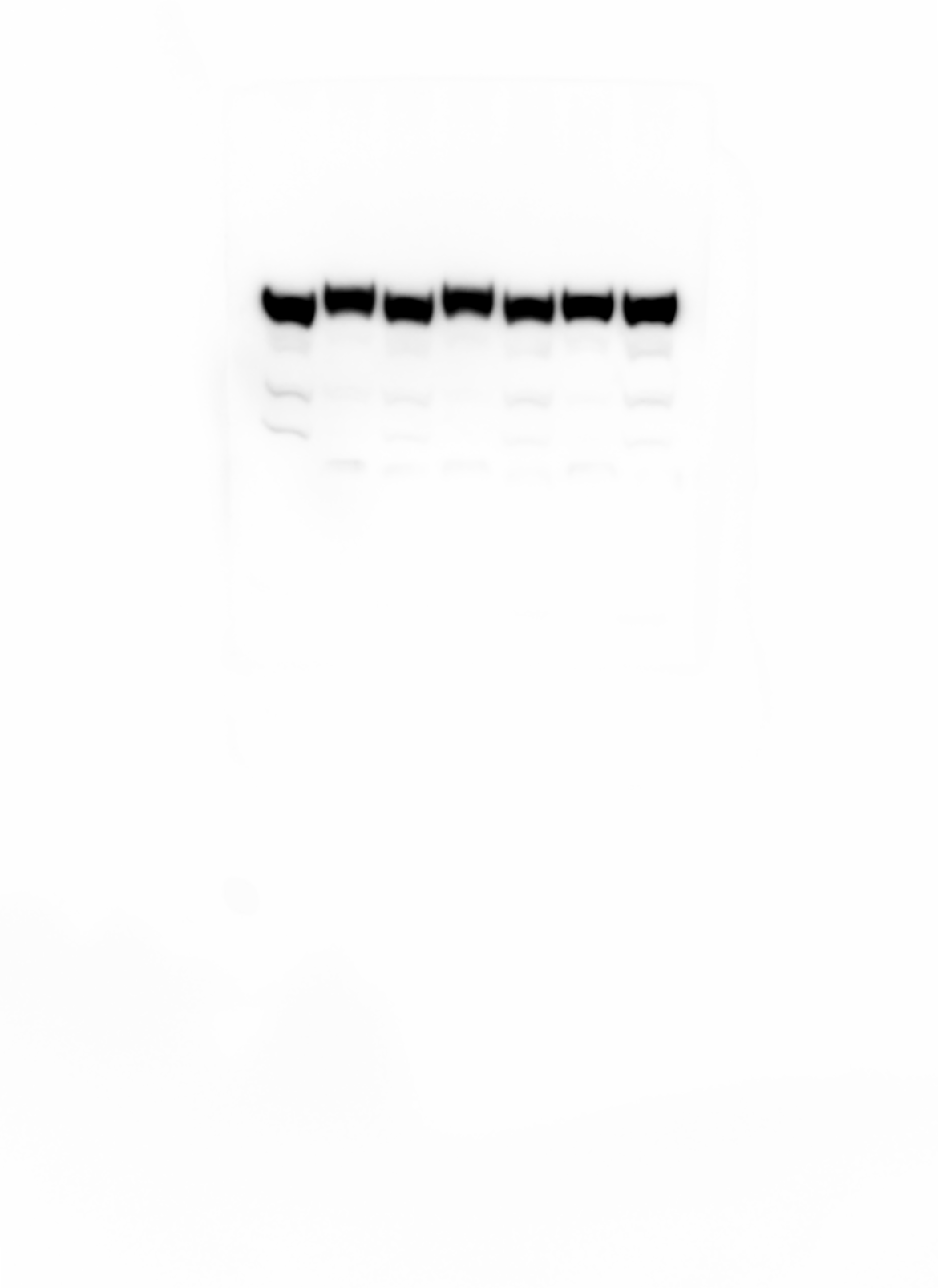

Supplement: Figure 5—source data 2. [file elife-77696-fig5-data2.zip › Figure 5-source data 2/STIM1-FLAG_WB_sourcedata.tif]

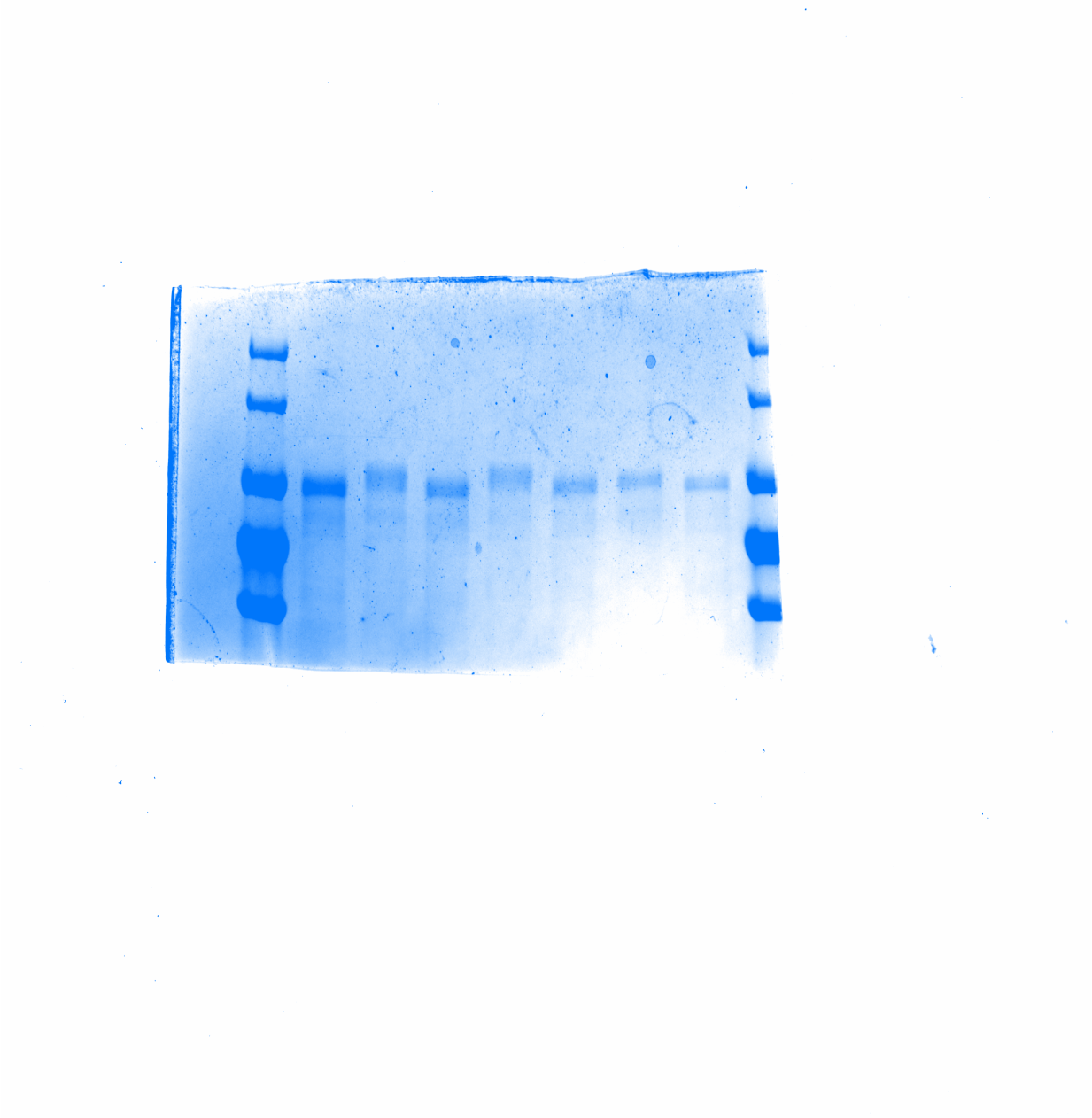

Supplement: Figure 5—source data 2. [file elife-77696-fig5-data2.zip › Figure 5-source data 2/STIM1-FLAG_Coomassie Blue Staining.tif]

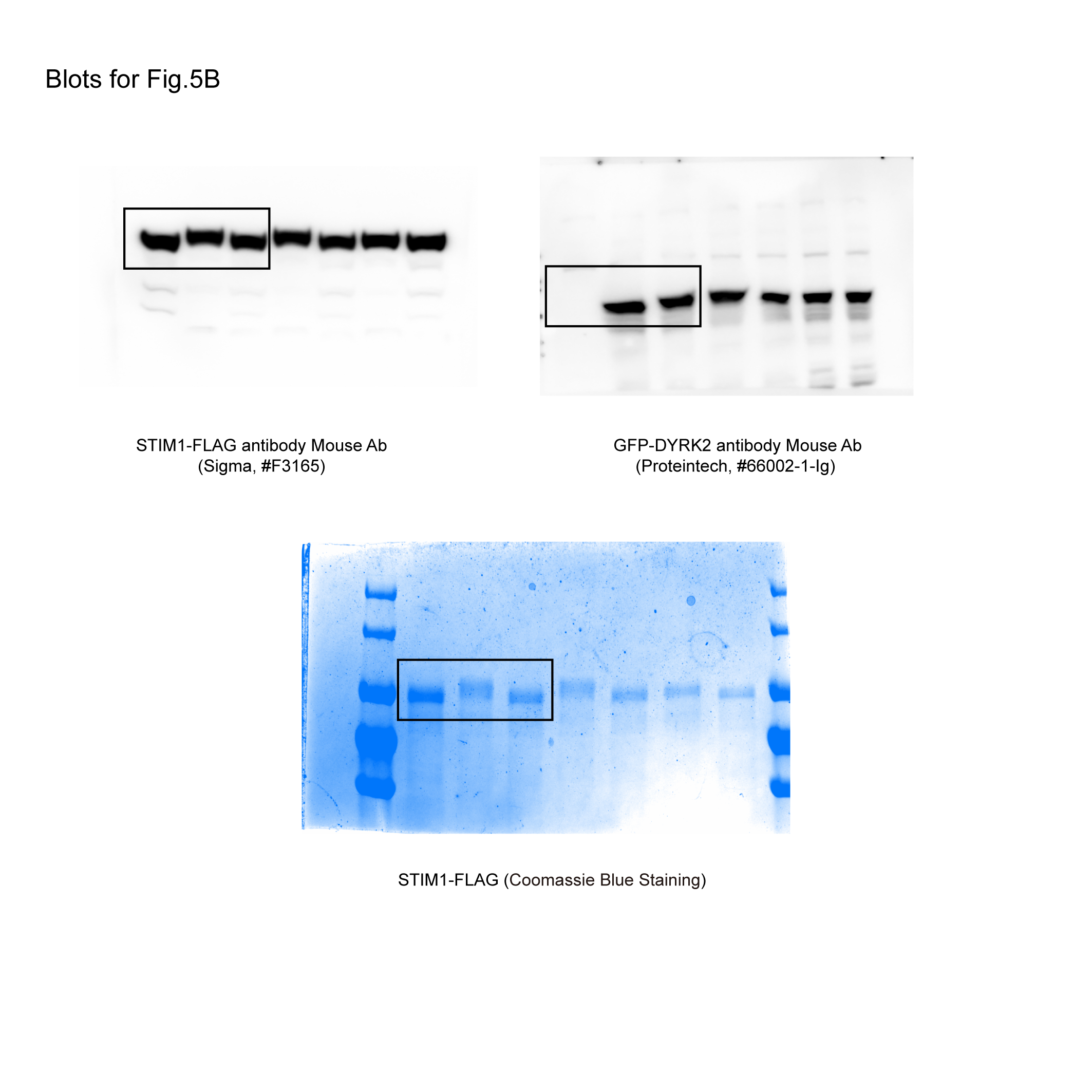

Supplement: Figure 5—source data 2. [file elife-77696-fig5-data2.zip › Figure 5-source data 2/Uncropped_Labeled_Gels_Fig5B.tif]

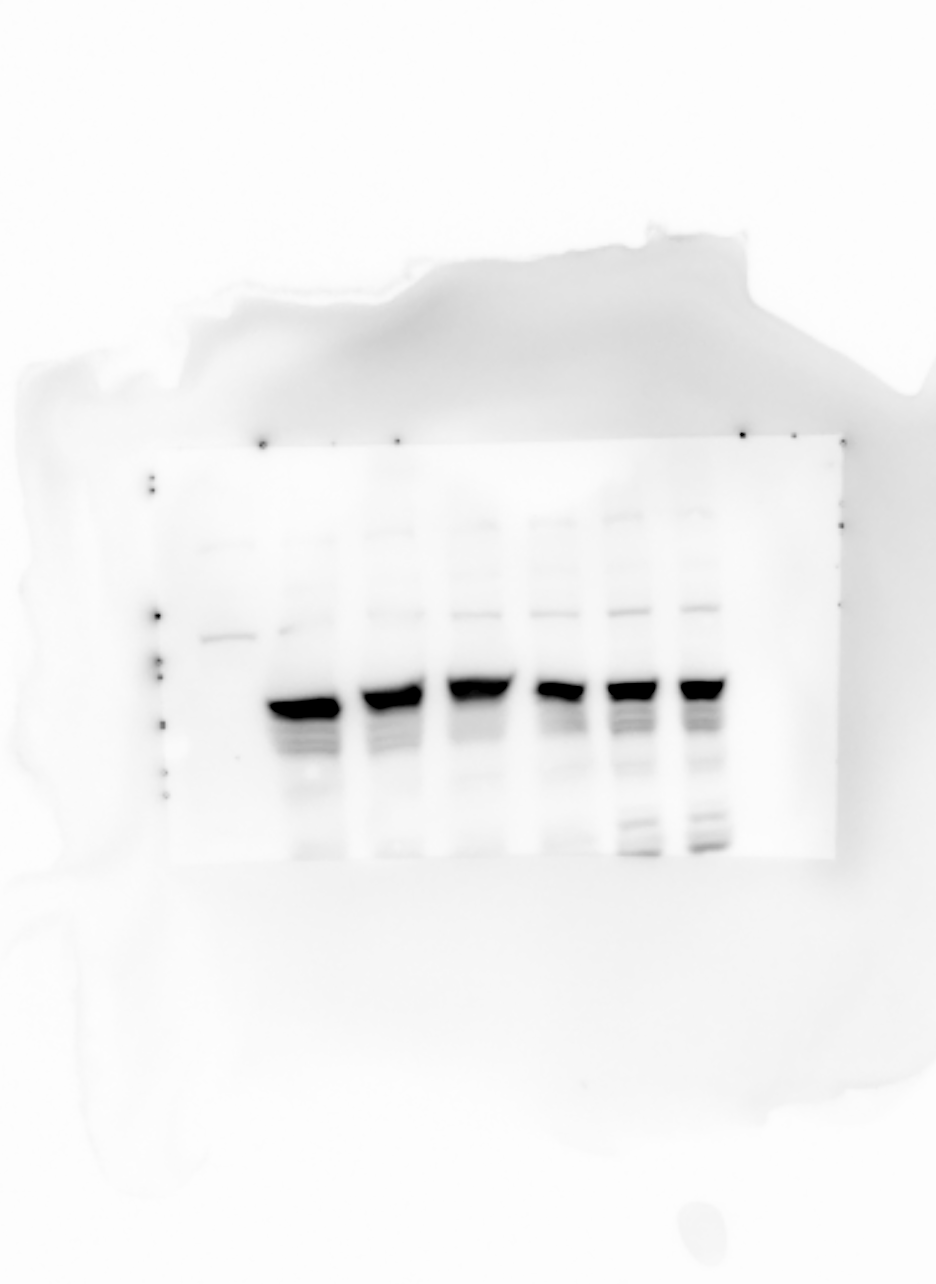

Supplement: Figure 5—source data 2. [file elife-77696-fig5-data2.zip › Figure 5-source data 2/GFP-DYRK2_WB_sourcedata.tif]

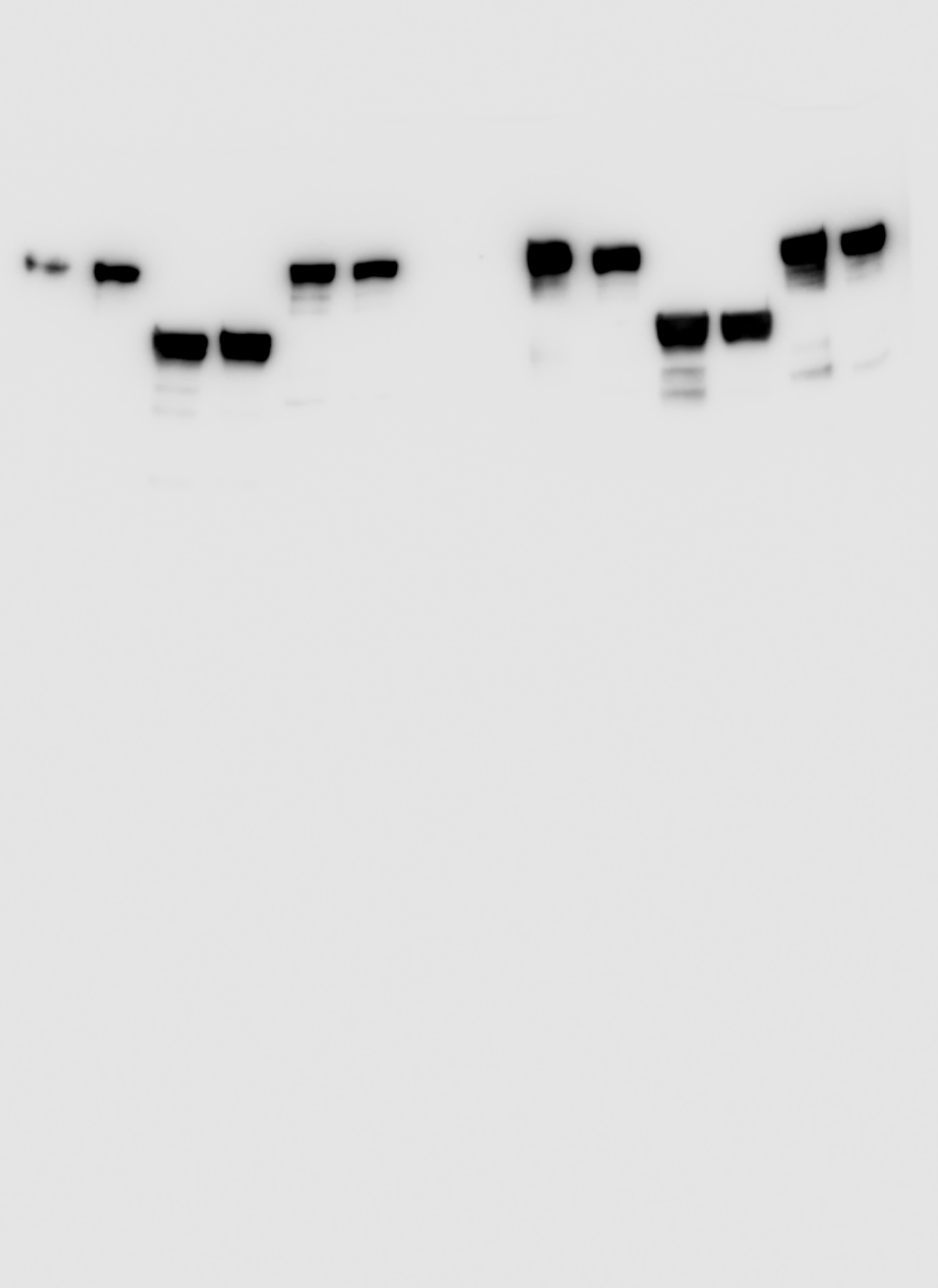

Supplement: Figure 5—source data 3. [file elife-77696-fig5-data3.zip › μ£¬σæ╜σÉìμûçΣ╗╢σñ╣/Figure5E-sourcedata/IP-STIM1-FLAG_WB_sourcedata.tif]

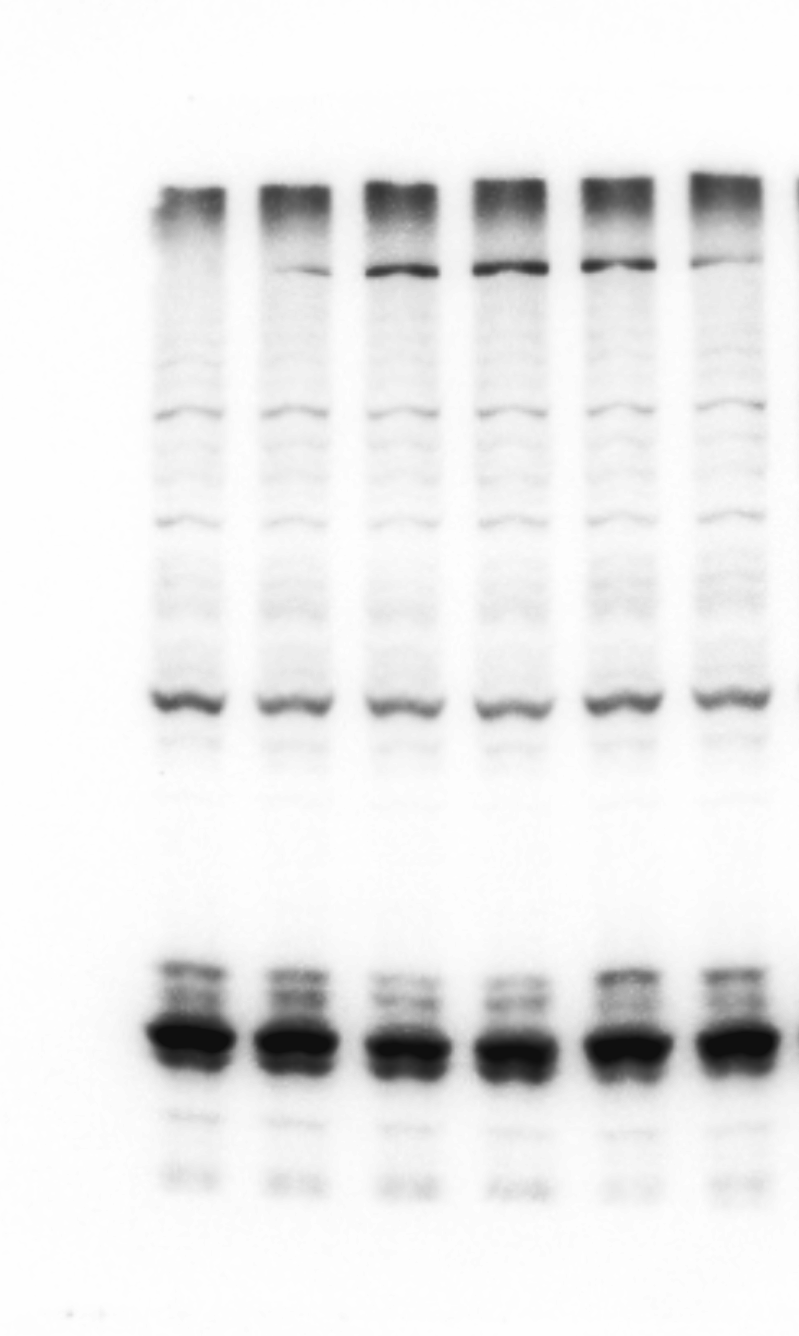

Supplement: Figure 5—source data 3. [file elife-77696-fig5-data3.zip › μ£¬σæ╜σÉìμûçΣ╗╢σñ╣/Figure5E-sourcedata/Input-GFP-Orai1_WB_sourcedata.tif]

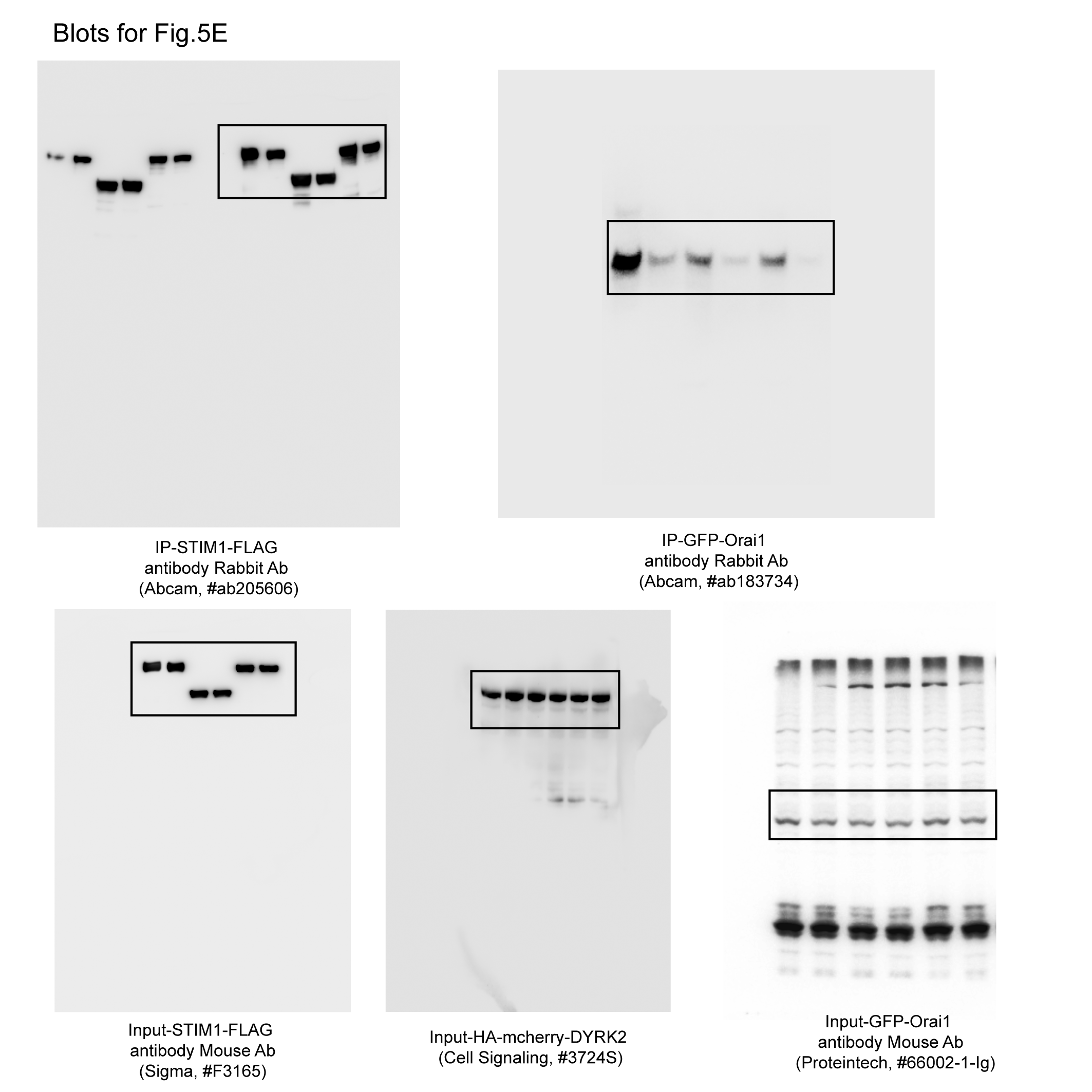

Supplement: Figure 5—source data 3. [file elife-77696-fig5-data3.zip › μ£¬σæ╜σÉìμûçΣ╗╢σñ╣/Figure5E-sourcedata/Uncropped_Labeled_Gels_Fig5E.tif]

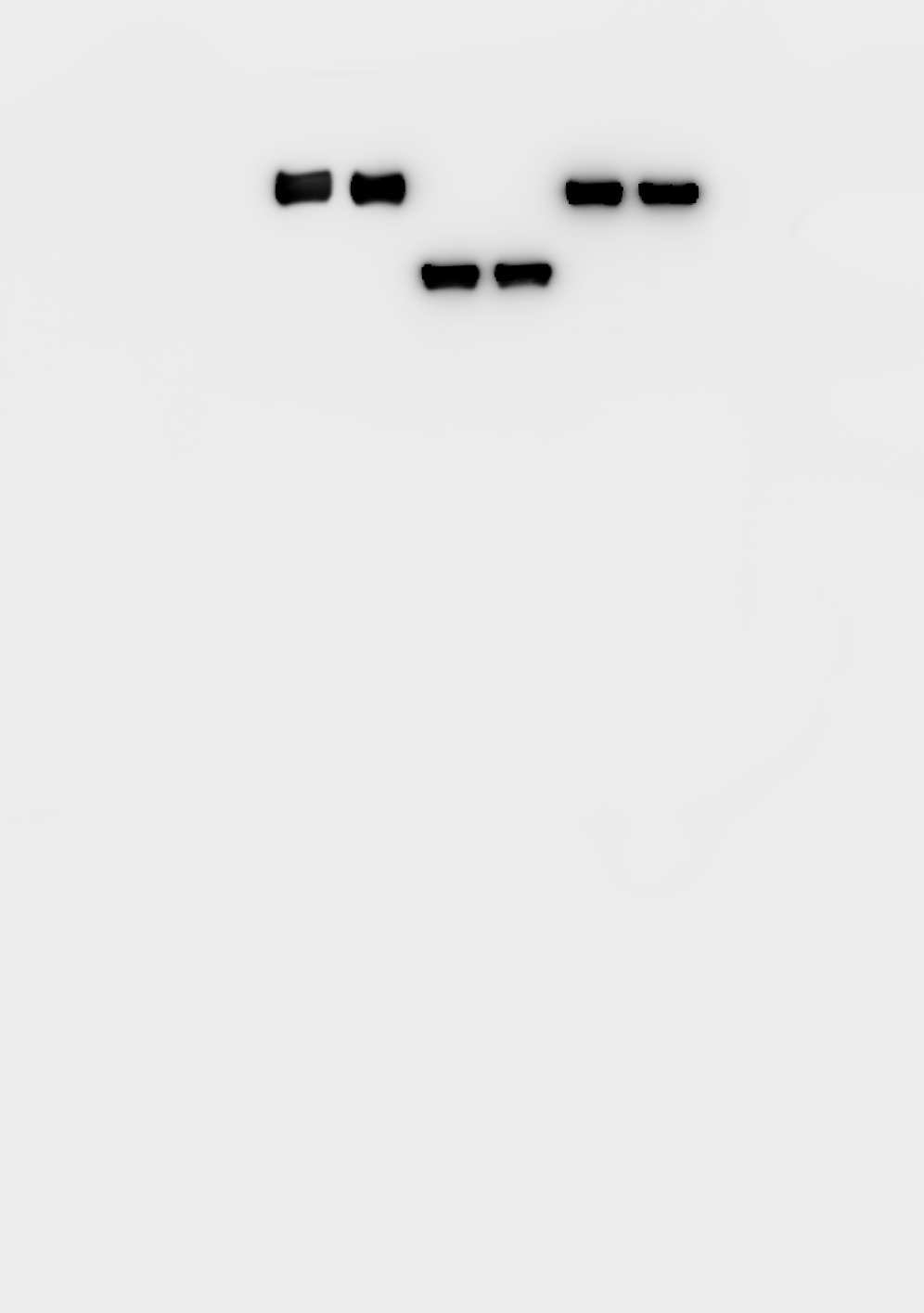

Supplement: Figure 5—source data 3. [file elife-77696-fig5-data3.zip › μ£¬σæ╜σÉìμûçΣ╗╢σñ╣/Figure5E-sourcedata/Input-STIM1-FLAG_WB_sourcedata.tif]

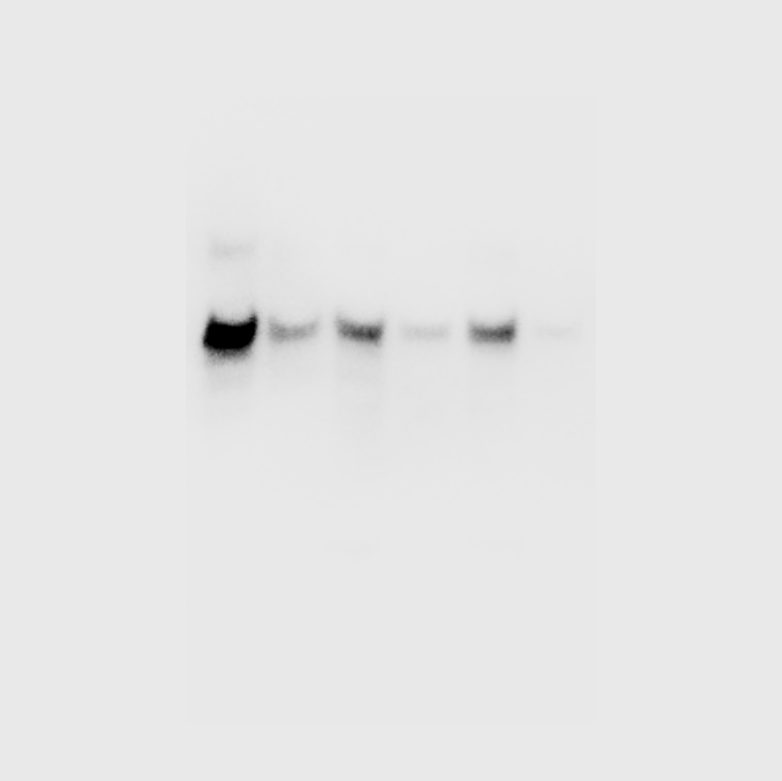

Supplement: Figure 5—source data 3. [file elife-77696-fig5-data3.zip › μ£¬σæ╜σÉìμûçΣ╗╢σñ╣/Figure5E-sourcedata/IP-GFP-Orai1_WB_sourcedata.tif]

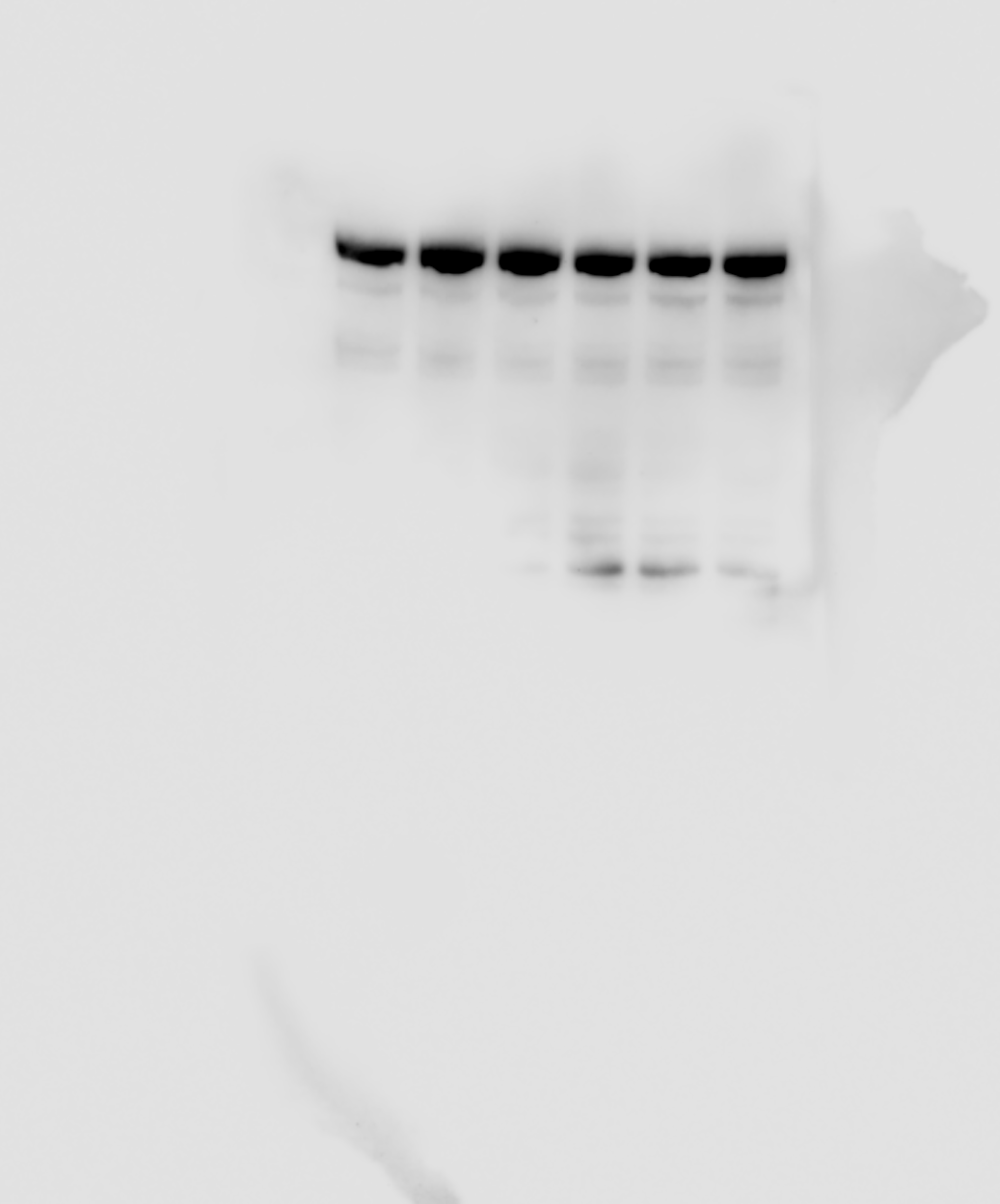

Supplement: Figure 5—source data 3. [file elife-77696-fig5-data3.zip › μ£¬σæ╜σÉìμûçΣ╗╢σñ╣/Figure5E-sourcedata/Input-HA-mcherry-DYRK2_WB_sourcedata.tif]

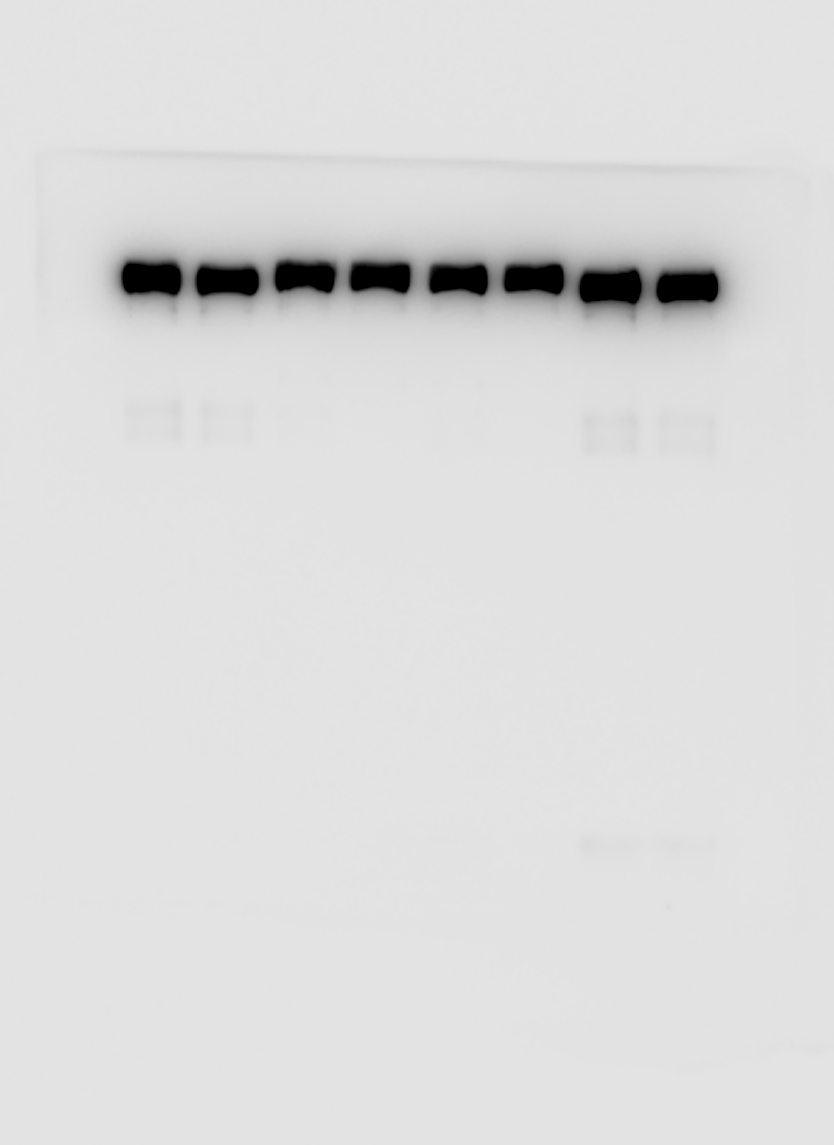

Supplement: Figure 5—source data 3. [file elife-77696-fig5-data3.zip › μ£¬σæ╜σÉìμûçΣ╗╢σñ╣/Figure5D-sourcedata/IP-STIM1-FLAG_WB_sourcedata.tif]

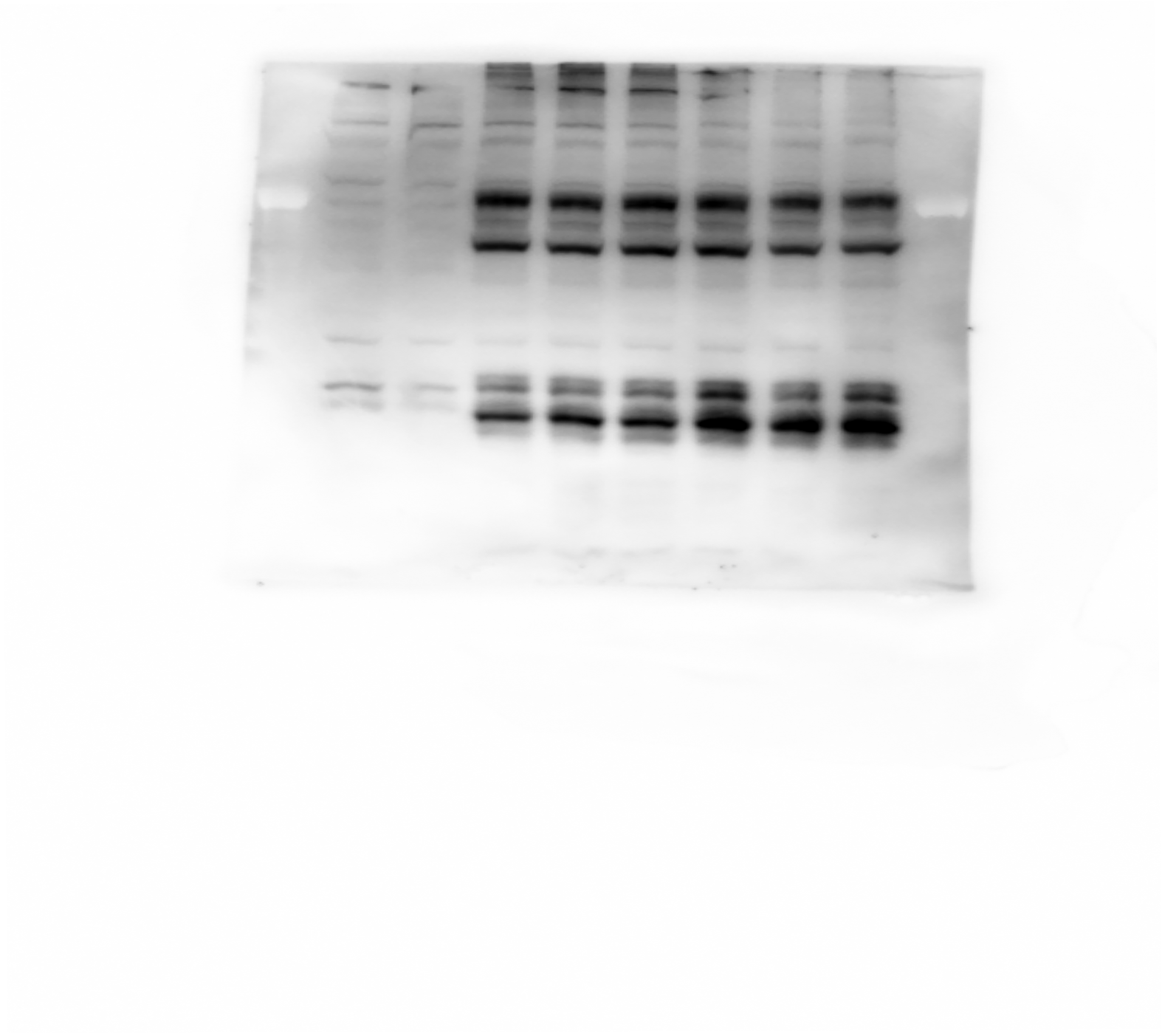

Supplement: Figure 5—source data 3. [file elife-77696-fig5-data3.zip › μ£¬σæ╜σÉìμûçΣ╗╢σñ╣/Figure5D-sourcedata/Input-GFP-Orai1_WB_sourcedata.tif]

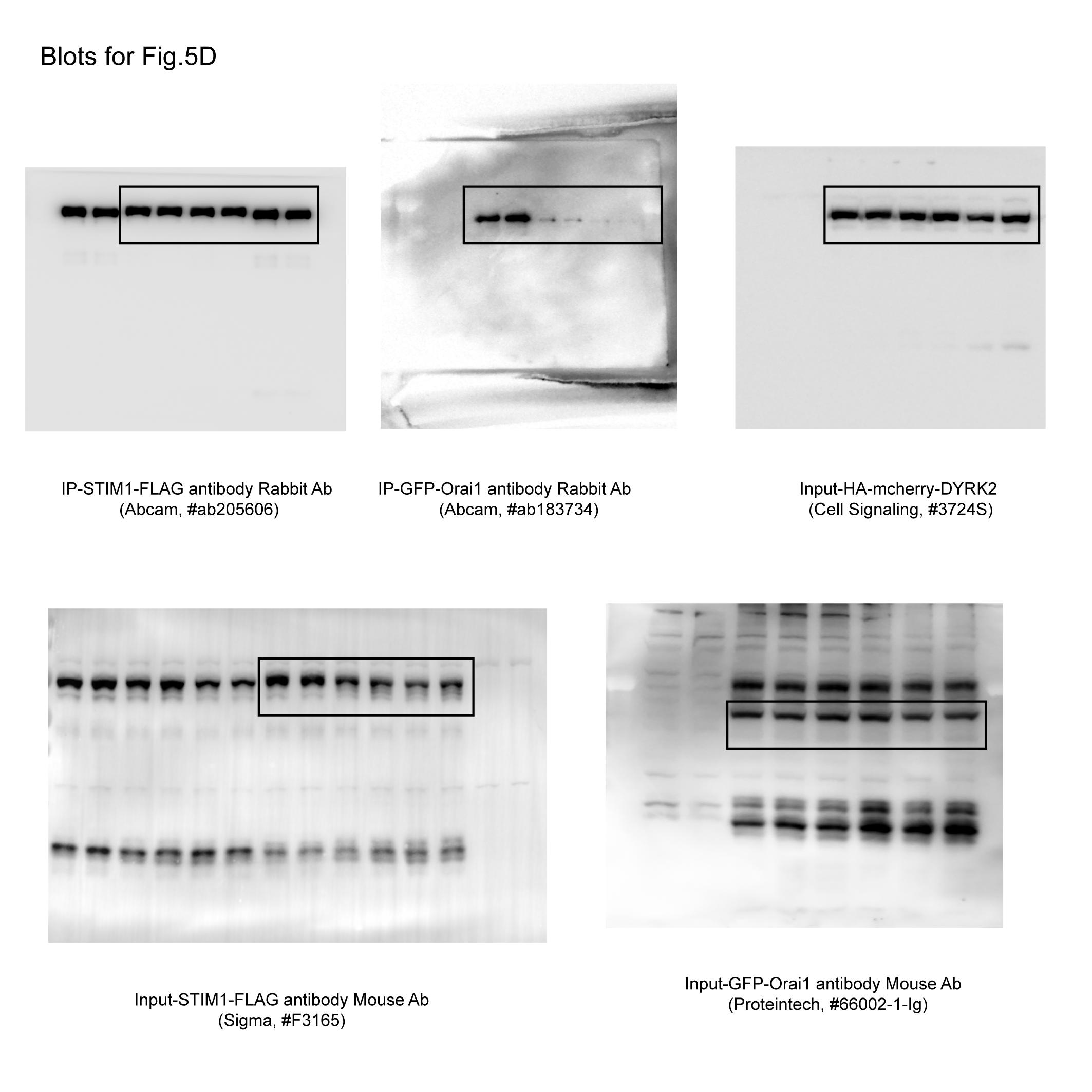

Supplement: Figure 5—source data 3. [file elife-77696-fig5-data3.zip › μ£¬σæ╜σÉìμûçΣ╗╢σñ╣/Figure5D-sourcedata/Uncropped_Labeled_Gels_Fig5D.tif]

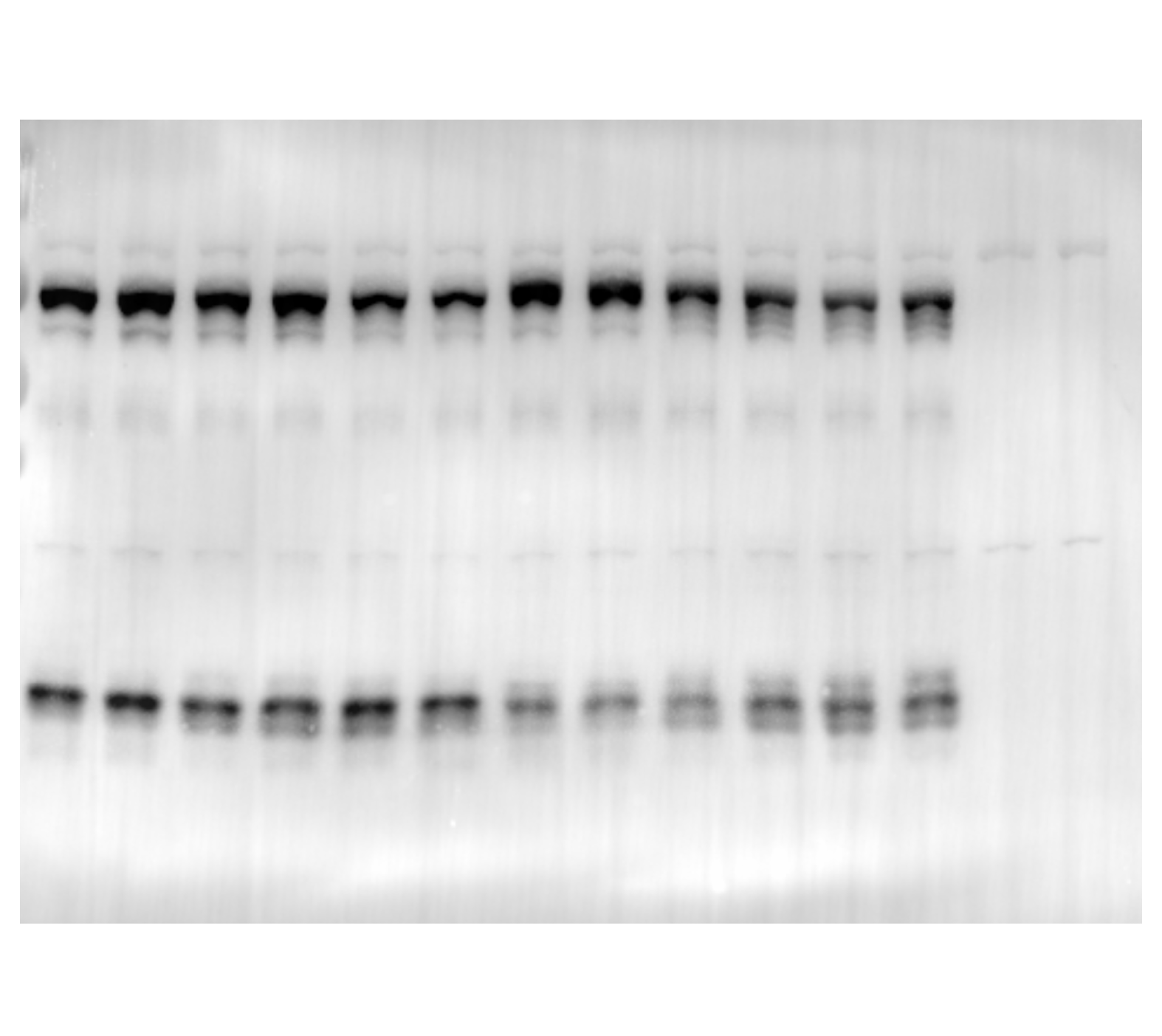

Supplement: Figure 5—source data 3. [file elife-77696-fig5-data3.zip › μ£¬σæ╜σÉìμûçΣ╗╢σñ╣/Figure5D-sourcedata/Input-STIM1-FLAG_WB_sourcedata.tif]

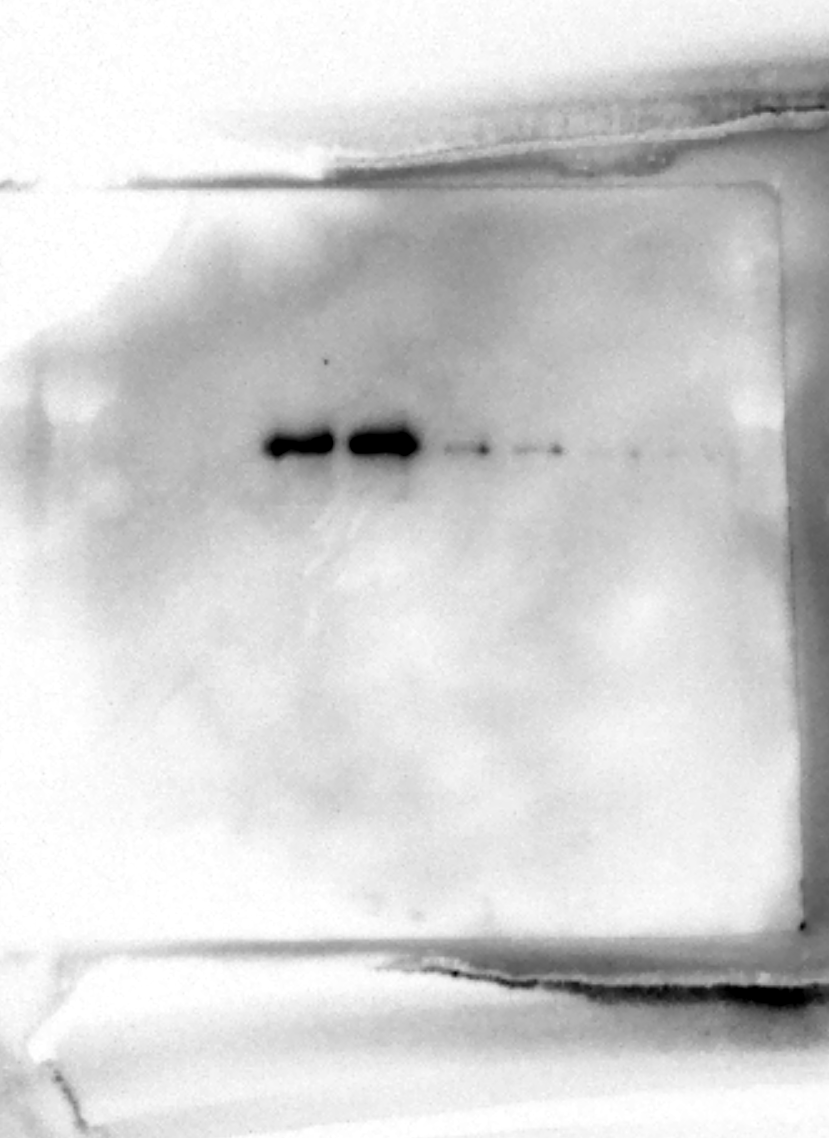

Supplement: Figure 5—source data 3. [file elife-77696-fig5-data3.zip › μ£¬σæ╜σÉìμûçΣ╗╢σñ╣/Figure5D-sourcedata/IP-Orai1_WB_sourcedata.tif]

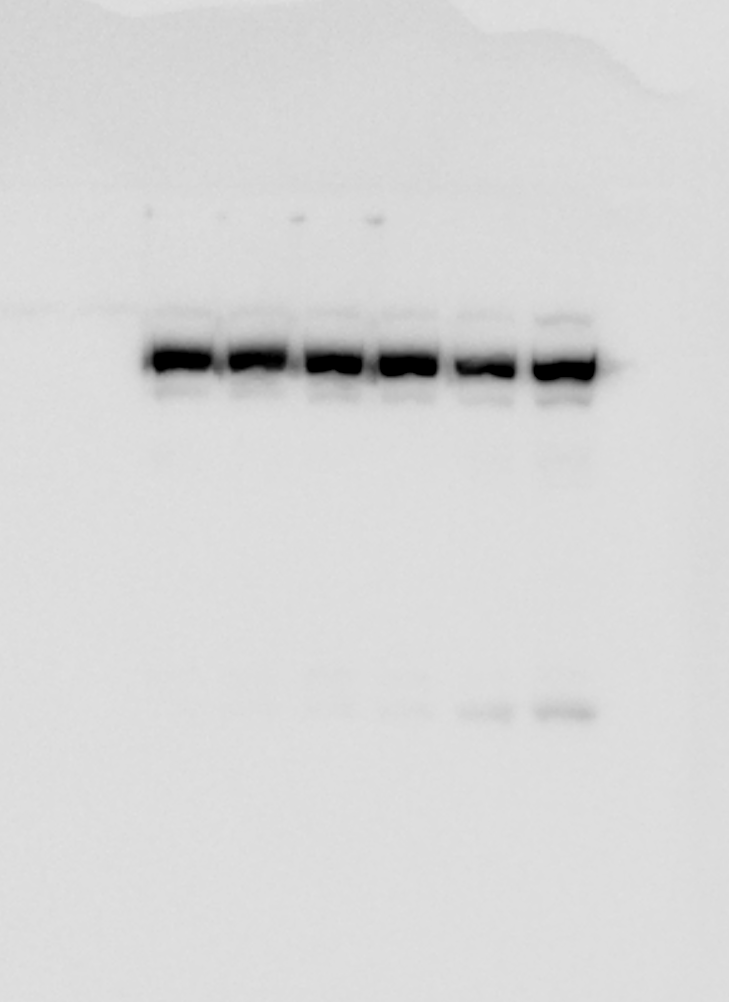

Supplement: Figure 5—source data 3. [file elife-77696-fig5-data3.zip › μ£¬σæ╜σÉìμûçΣ╗╢σñ╣/Figure5D-sourcedata/Input-HA-mcherry-DYRK2_WB_sourcedata.tif]

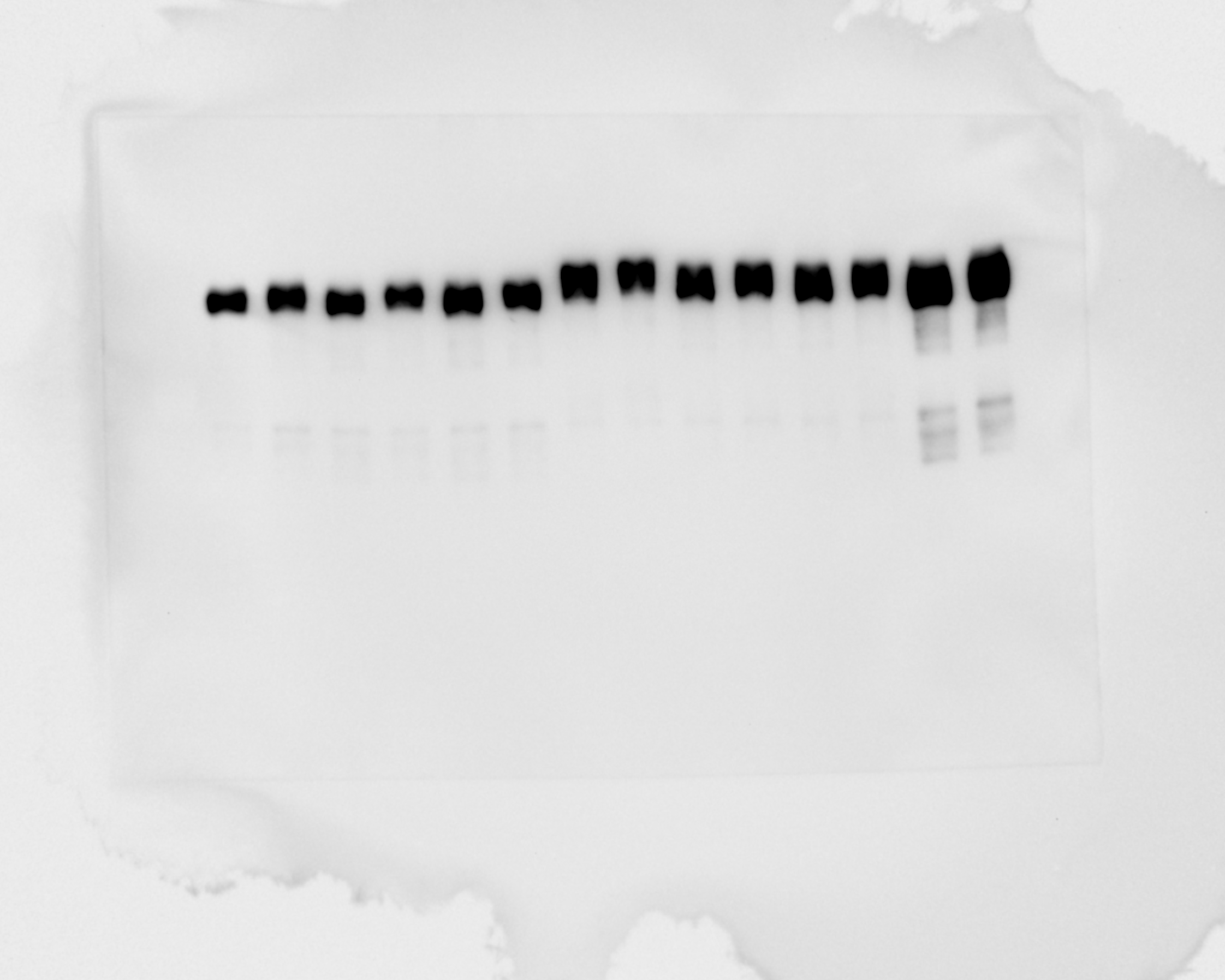

Supplement: Figure 5—source data 4. [file elife-77696-fig5-data4.zip › Figure 5-source data 4/IP-STIM1-FLAG_WB_sourcedata.tif]

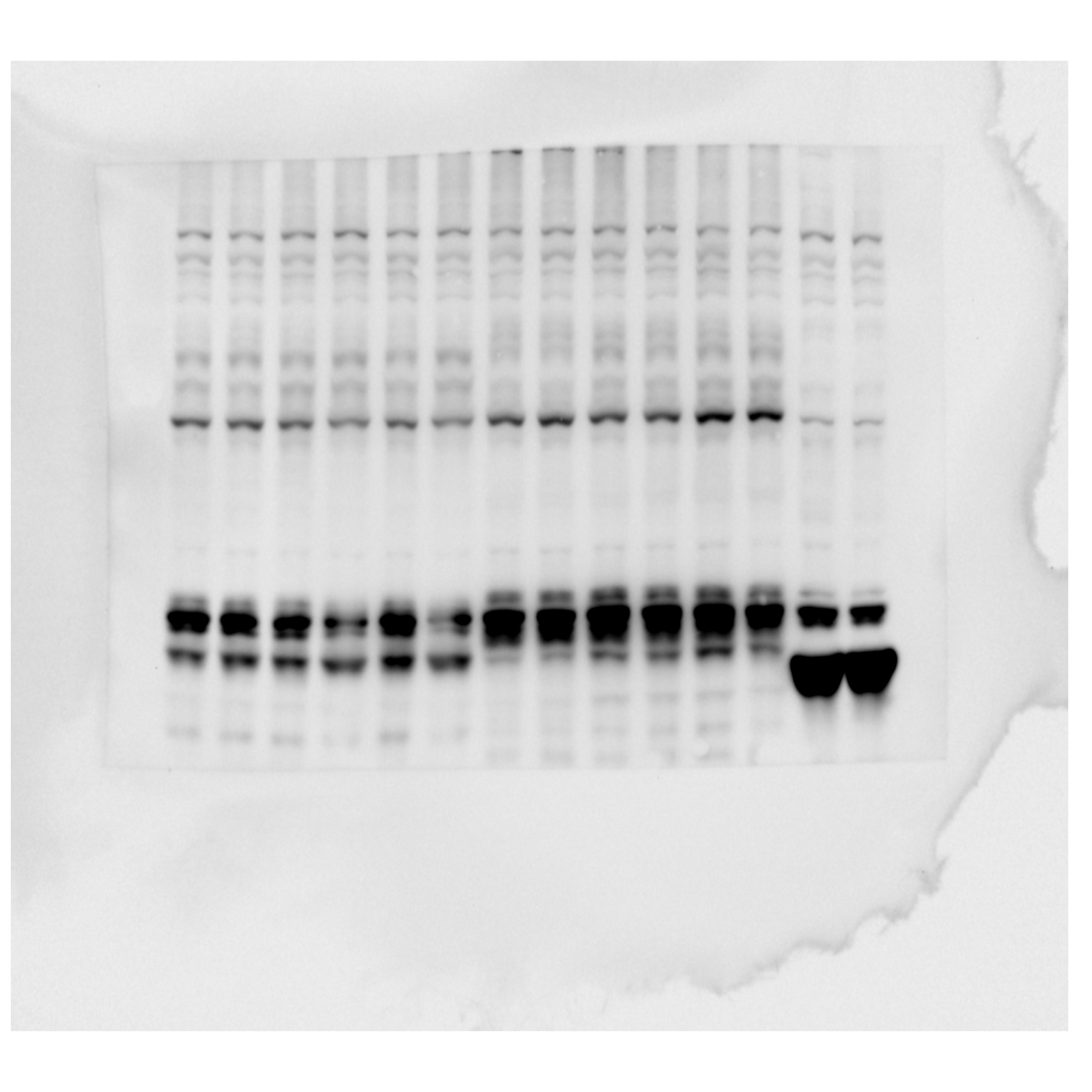

Supplement: Figure 5—source data 4. [file elife-77696-fig5-data4.zip › Figure 5-source data 4/Input-GFP-Orai1_WB_sourcedata.tif]

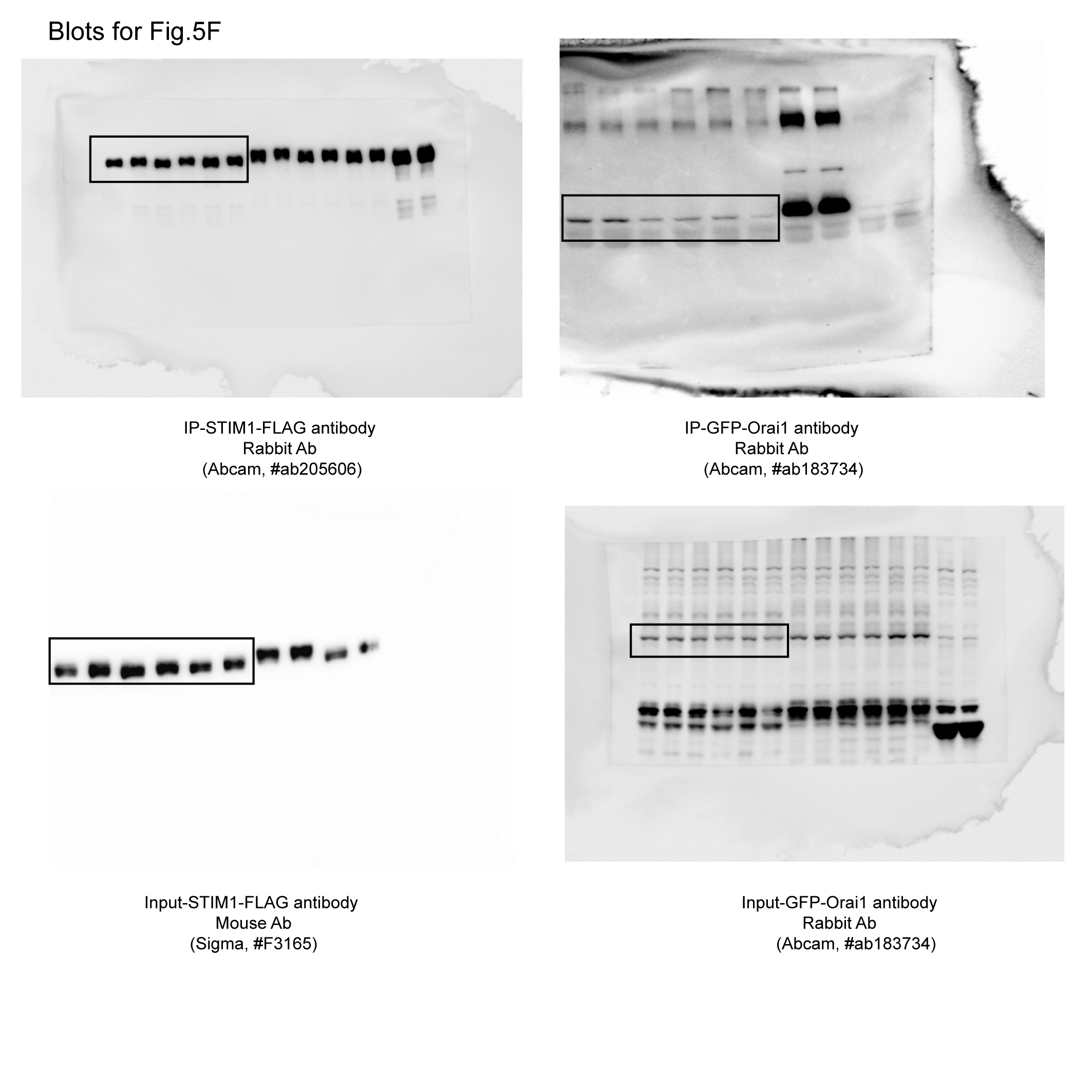

Supplement: Figure 5—source data 4. [file elife-77696-fig5-data4.zip › Figure 5-source data 4/Uncropped_Labeled_Gels_Fig5F.tif]

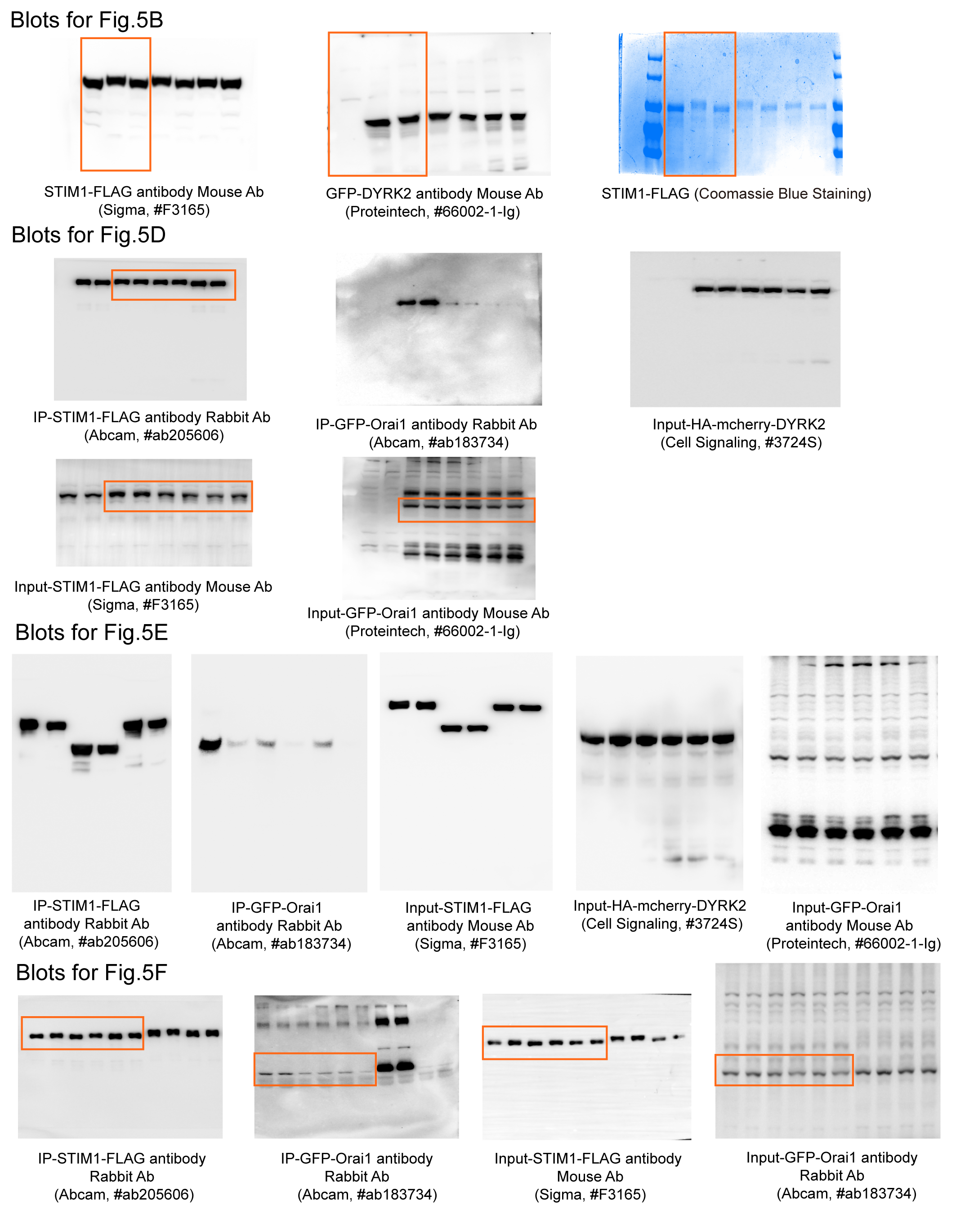

Supplement: Figure 5—source data 4. [file elife-77696-fig5-data4.zip › Figure 5-source data 4/Uncropped_Labeled_Gels_Fig5_summery.tif]

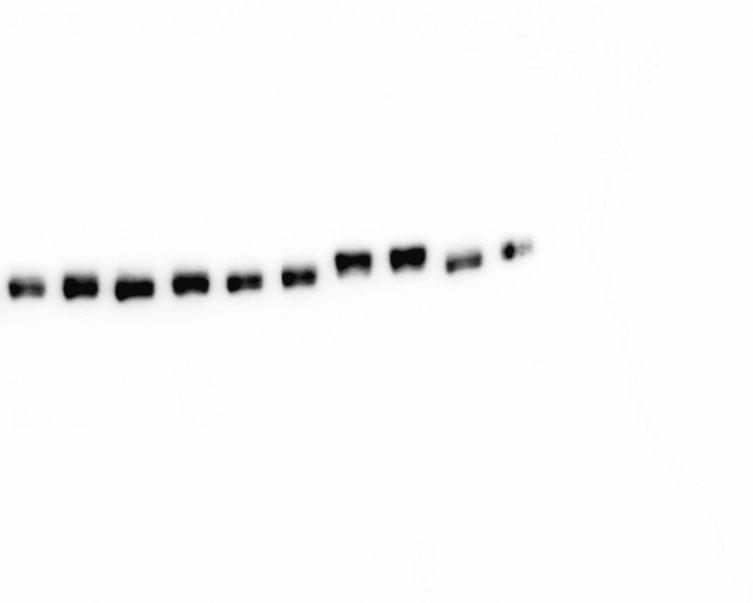

Supplement: Figure 5—source data 4. [file elife-77696-fig5-data4.zip › Figure 5-source data 4/Input-STIM1-FLAG_WB_sourcedata.tif]

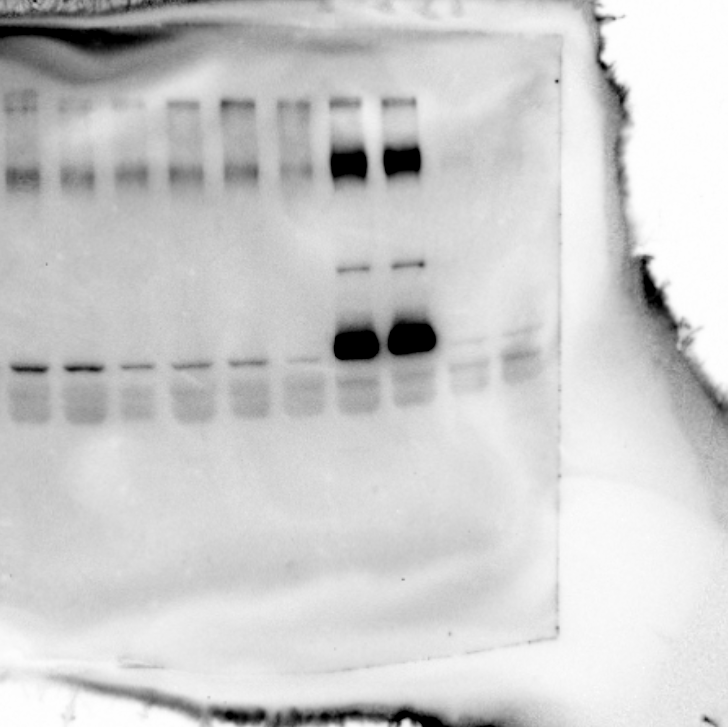

Supplement: Figure 5—source data 4. [file elife-77696-fig5-data4.zip › Figure 5-source data 4/IP-GFP-Orai1_WB_sourcedata.tif]
