# Supplementary material for: Selective inhibition reveals the regulatory function of DYRK2 in protein synthesis and calcium entry
Source: eLife. 2022 Apr 19;11:e77696. doi: 10.7554/eLife.77696 (PMC9113749; doi:10.7554/eLife.77696)
Supplement: Supplementary file 1. [file elife-77696-supp1.docx]

**1c**


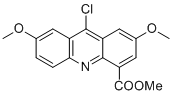
**
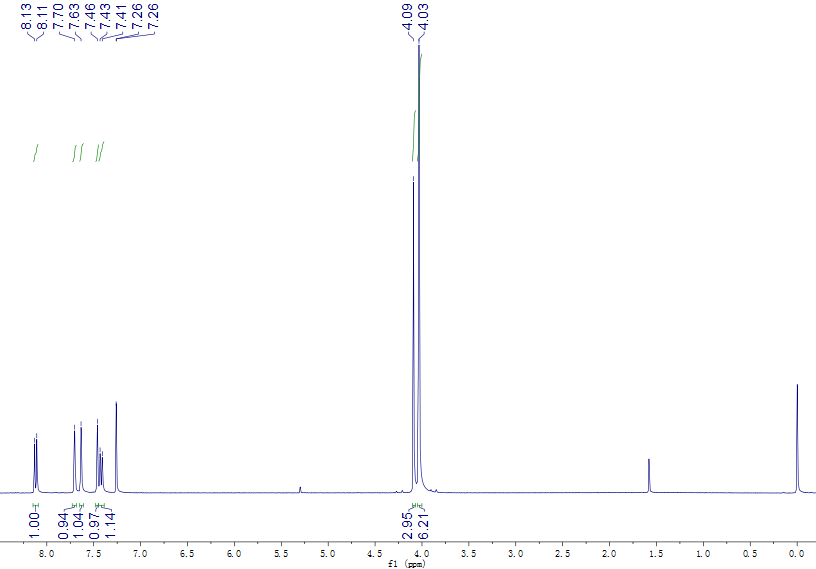
**

^1^H NMR, CDCl_3_

400M, 298K

^1^H-NMR of **1c.**

**
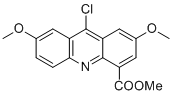

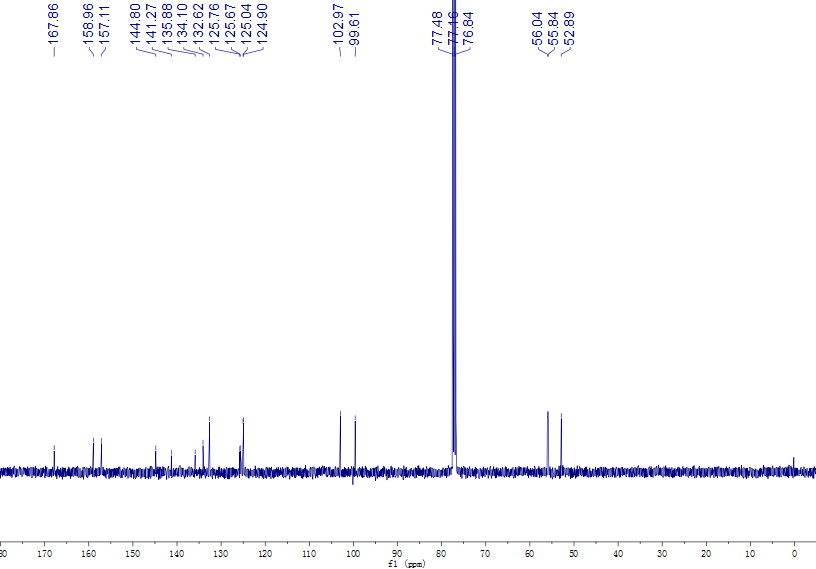
**

^13^C NMR, CDCl_3_

101M, 298K

^13^C-NMR of **1c.**

**1**


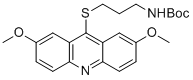
**
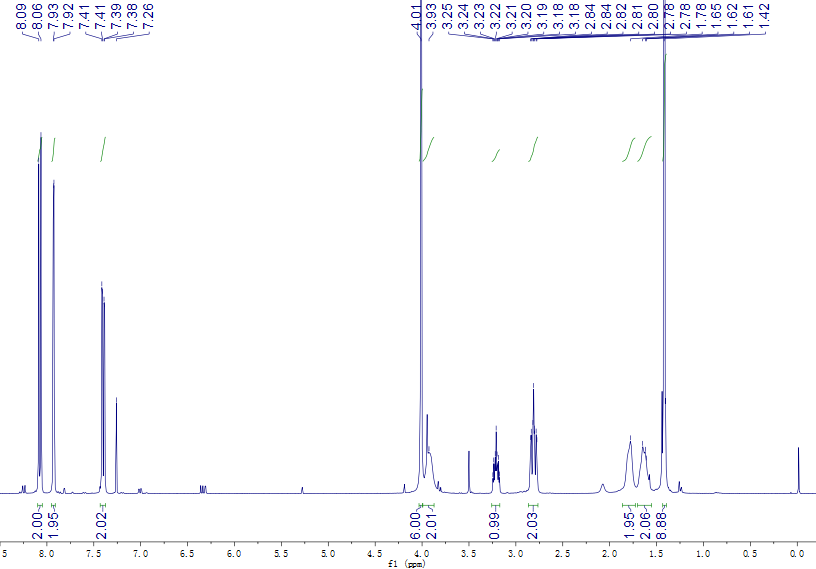
**

^1^H NMR, CDCl_3_

400M, 298K

^1^H-NMR of **1.**

^13^C NMR, CDCl_3_

101M, 298K


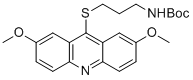
**
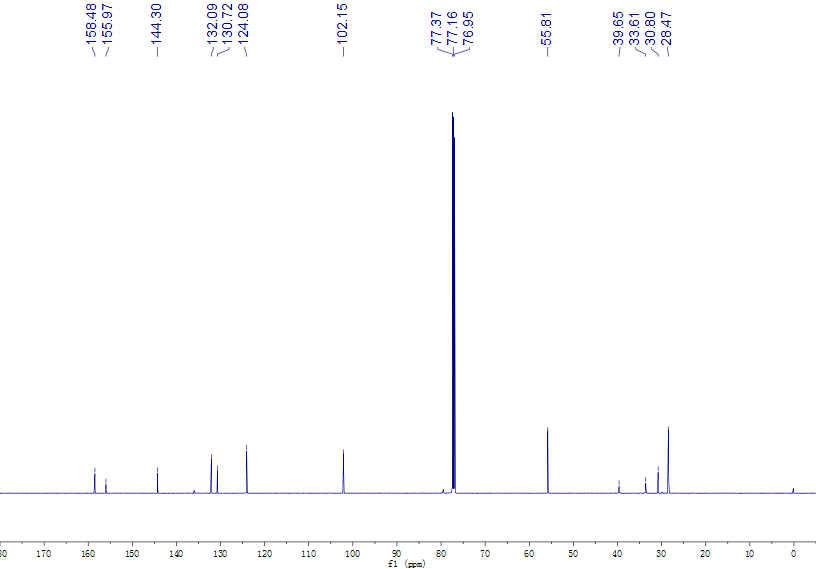
**

^13^C-NMR of **1.**

**1c'**

**
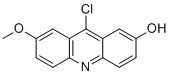

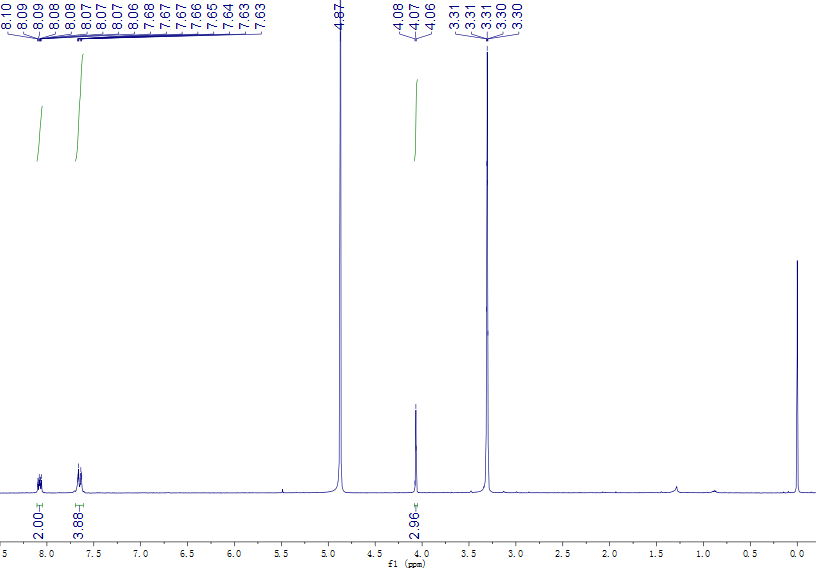
**

^1^H NMR, MeOD

400M, 298K

H_2_O

^1^H-NMR of **1c'.**

**
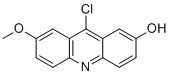

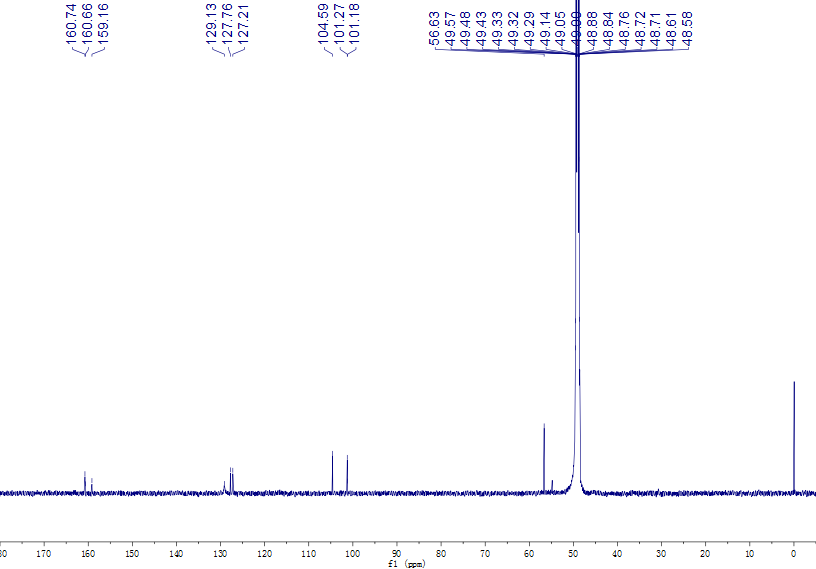
**

^13^C NMR, MeOD

151M, 298K

^13^C-NMR of **1c'.**

**2**


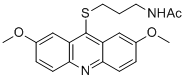
**
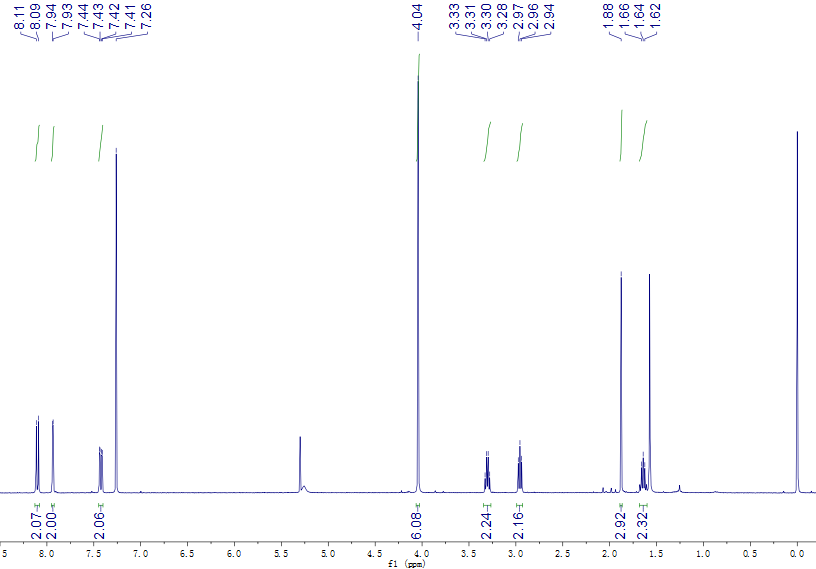
**

^1^H NMR, CDCl_3_

400M, 298K

^1^H-NMR of **2.**

**
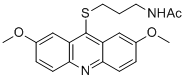

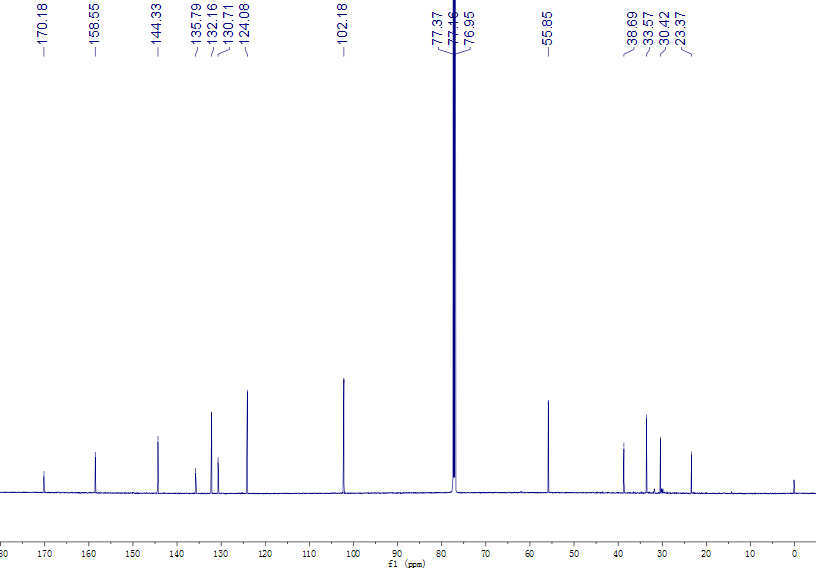
**

^13^C NMR, CDCl_3_

101M, 298K

^13^C-NMR of **2.**

**3**


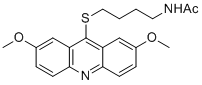
**
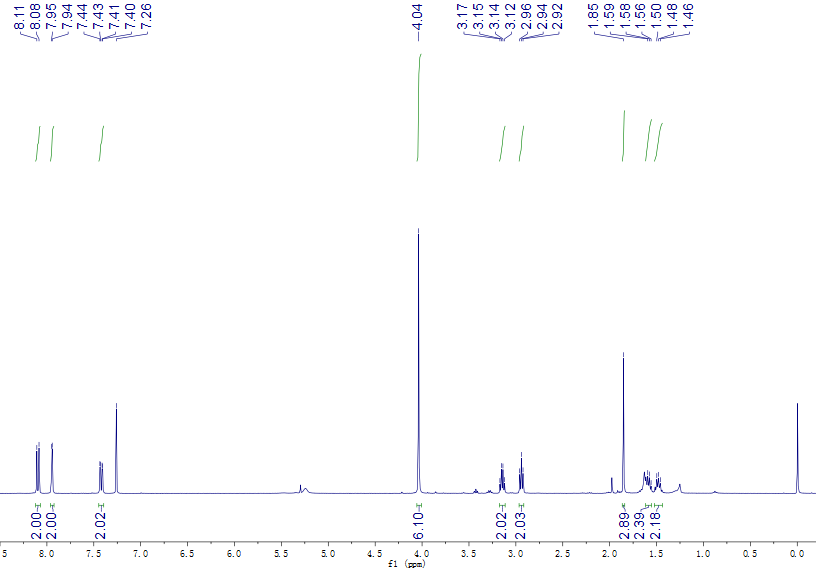
**

^1^H NMR, CDCl_3_

400M, 298K

^1^H-NMR of **3.**

**
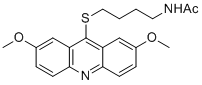

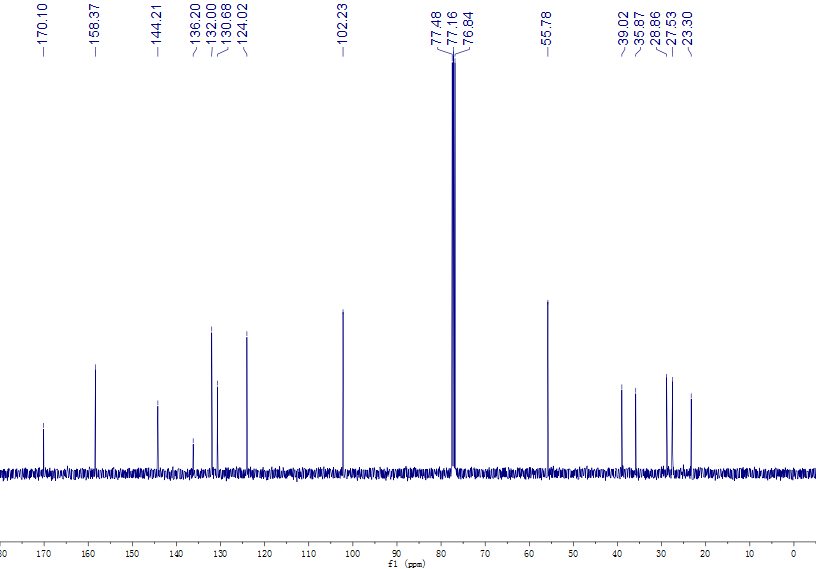
**

^13^C NMR, CDCl_3_

101M, 298K

^13^C-NMR of **3.**

**4**


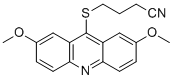
**
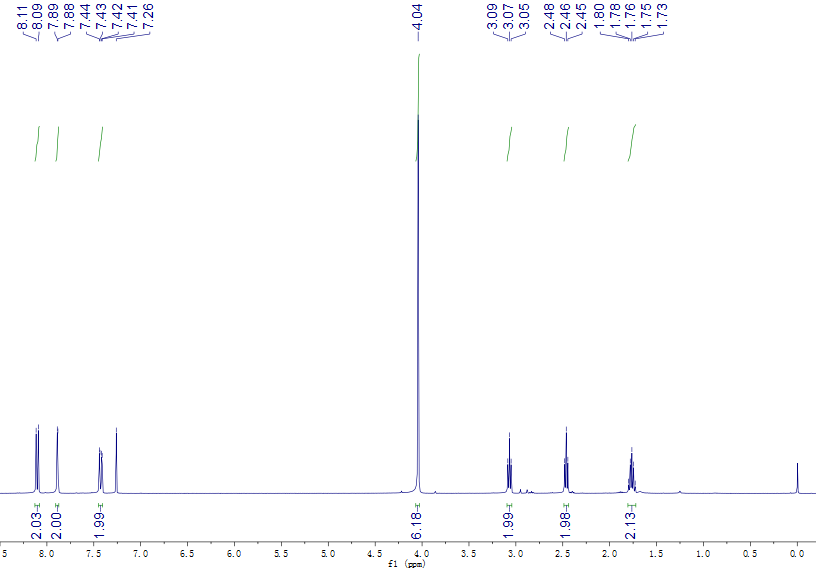
**

^1^H NMR, CDCl_3_

400M, 298K

^1^H-NMR of **4.**

**
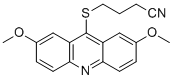

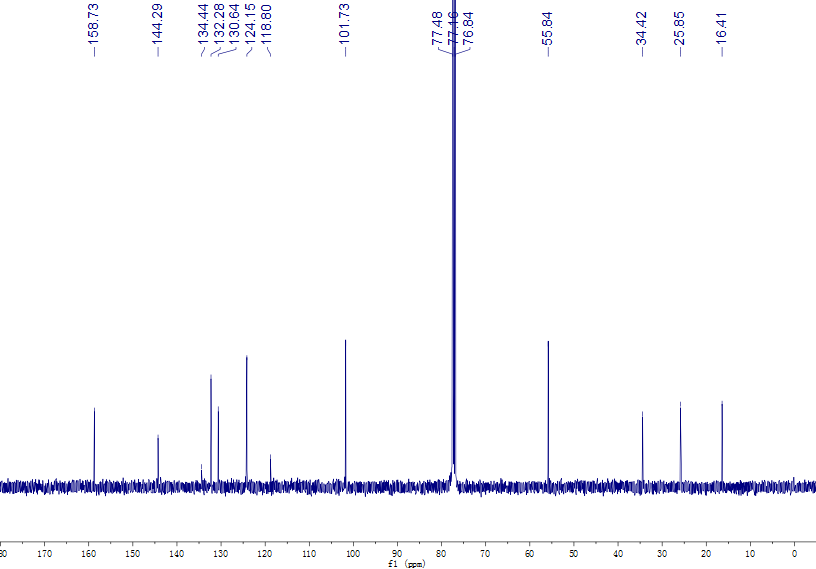
**

^13^C NMR, CDCl_3_

101M, 298K

^13^C-NMR of **4.**

**5a**


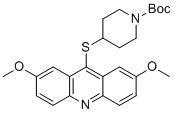
**
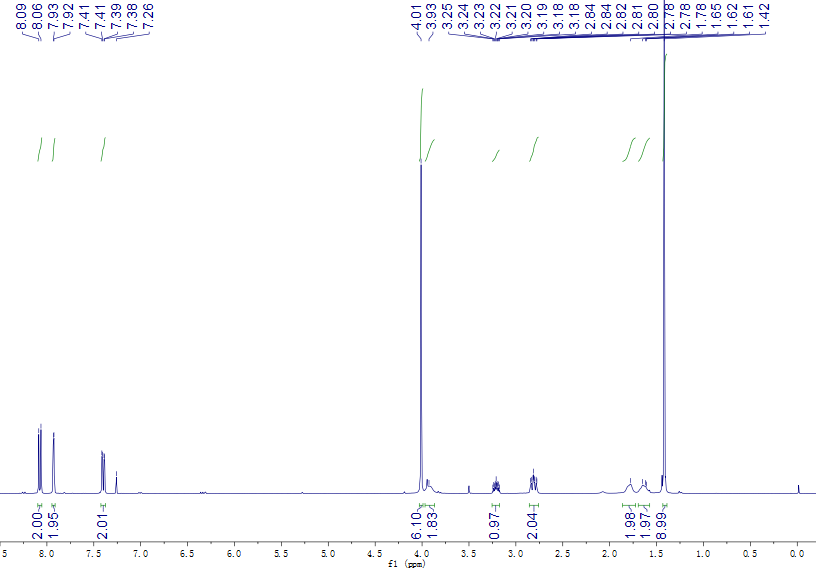
**

^1^H NMR, CDCl_3_

400M, 298K

^1^H-NMR of **5a.**

**
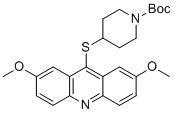

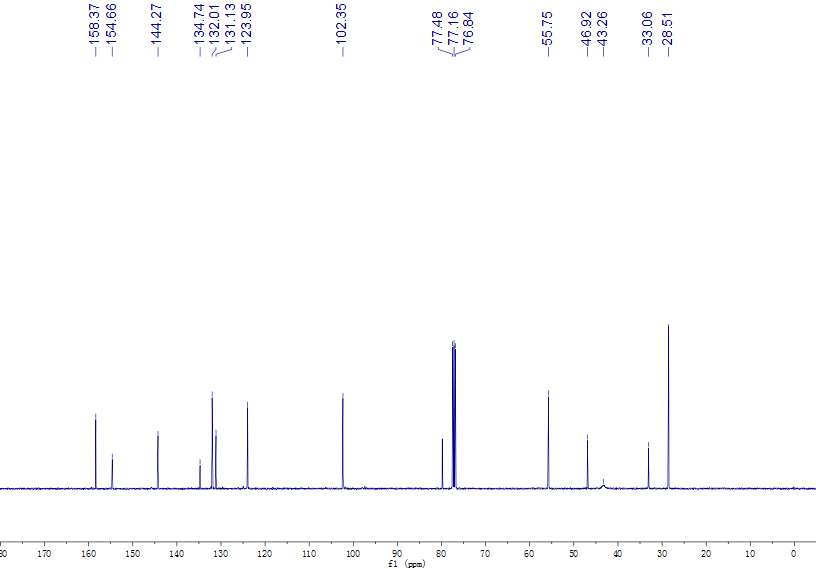
**

^13^C NMR, CDCl_3_

101M, 298K

^13^C-NMR of **5a.**

**5**


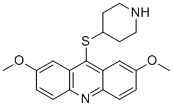
**
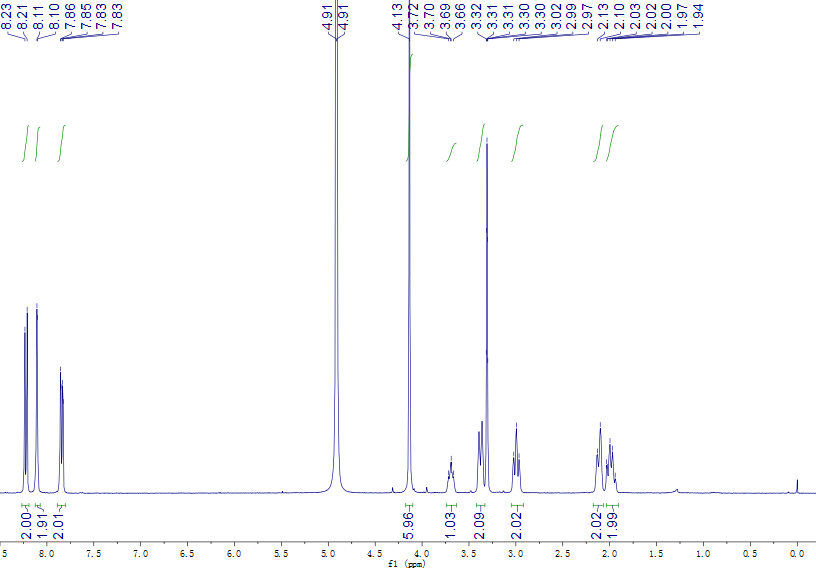
**

^1^H NMR, MeOD

400M, 298K

H_2_O

^1^H-NMR of **5.**

**
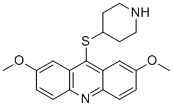

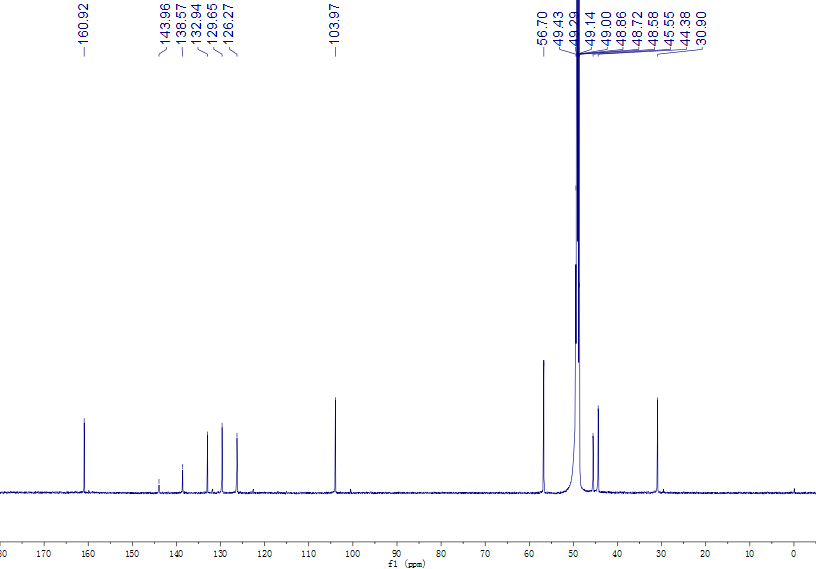
**

^13^C NMR, MeOD

101M, 298K

^13^C-NMR of **5.**

**6a**

**
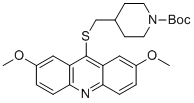

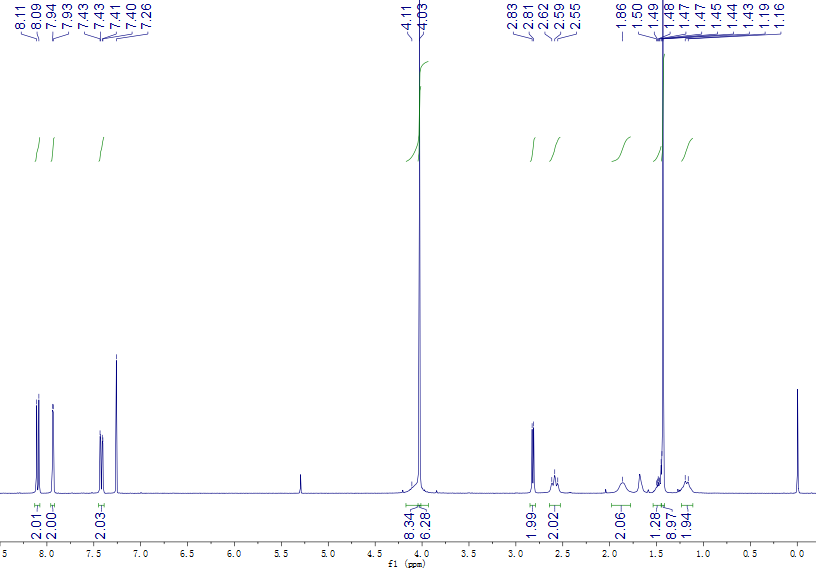
**

^1^H NMR, CDCl_3_

400M, 298K

^1^H-NMR of **6a.**

**
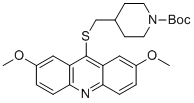

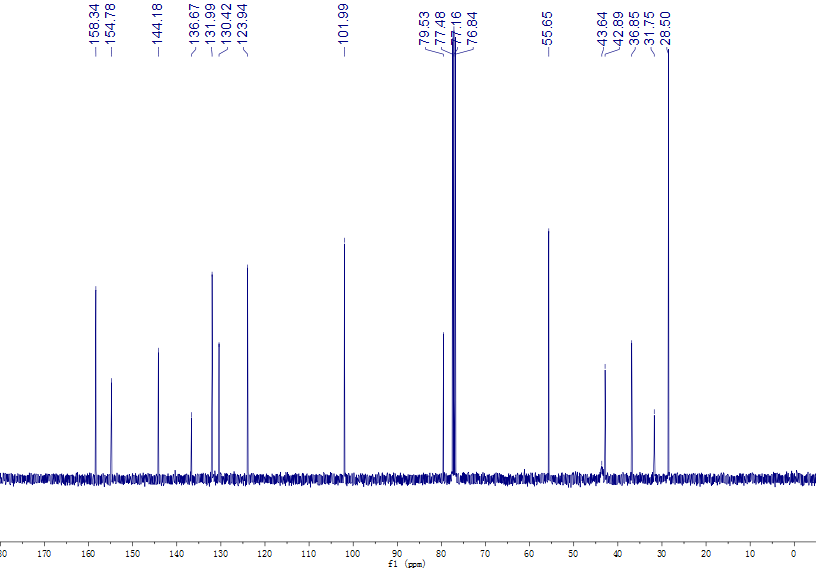
**

^13^C NMR, CDCl_3_

101M, 298K

^13^C-NMR of **6a.**

**6**


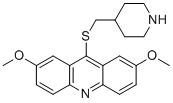
**
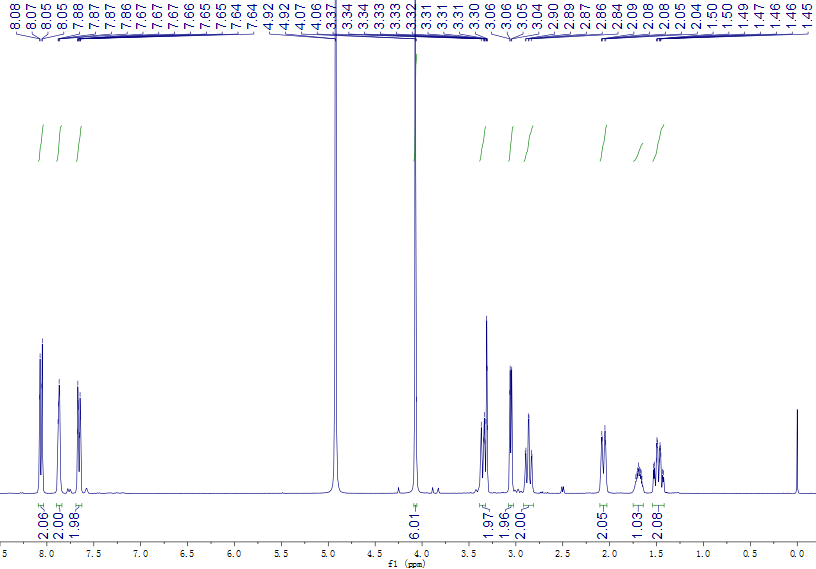
**

^1^H NMR, MeOD

400M, 298K

H_2_O

^1^H-NMR of **6.**

**
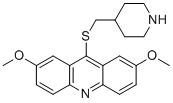

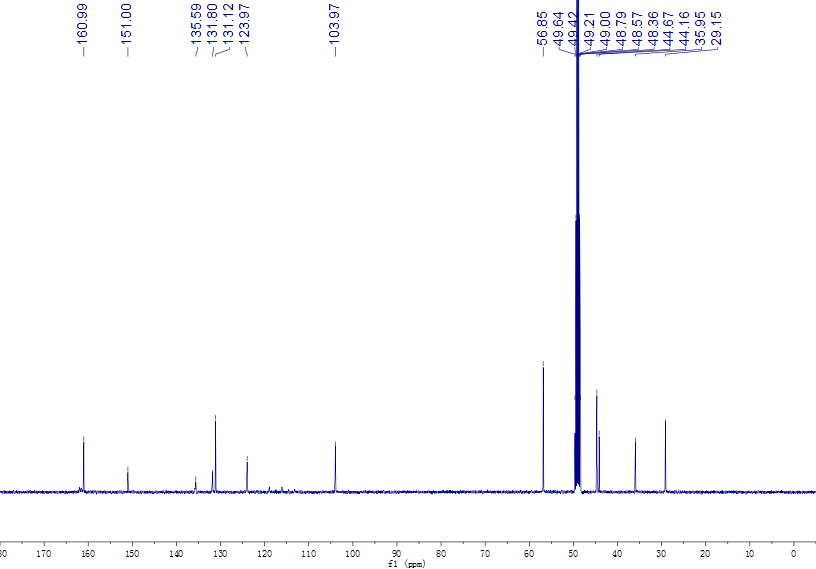
**

^13^C NMR, MeOD

101M, 298K

^13^C-NMR of **6.**

**7a**


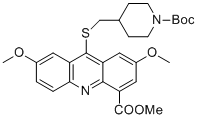
**
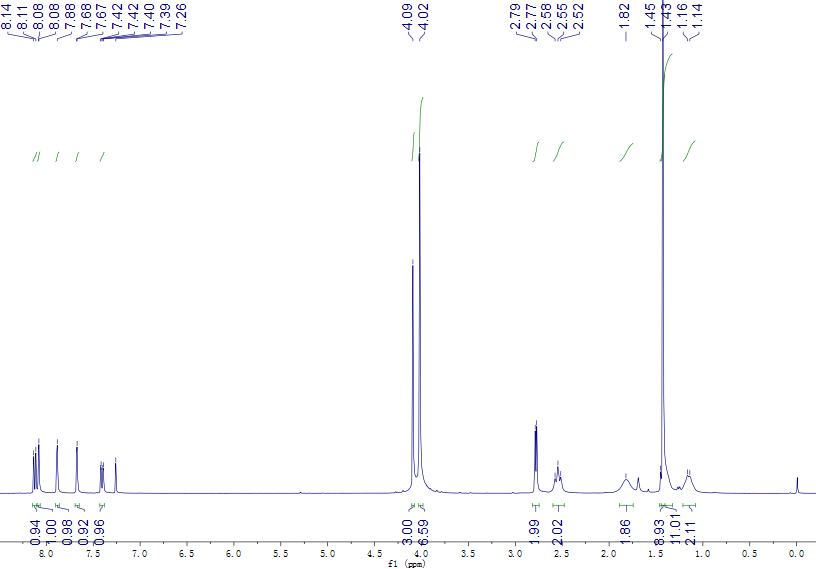
**

^1^H NMR, CDCl_3_

400M, 298K

^1^H-NMR of **7a.**

**
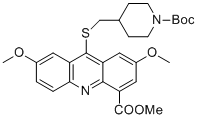

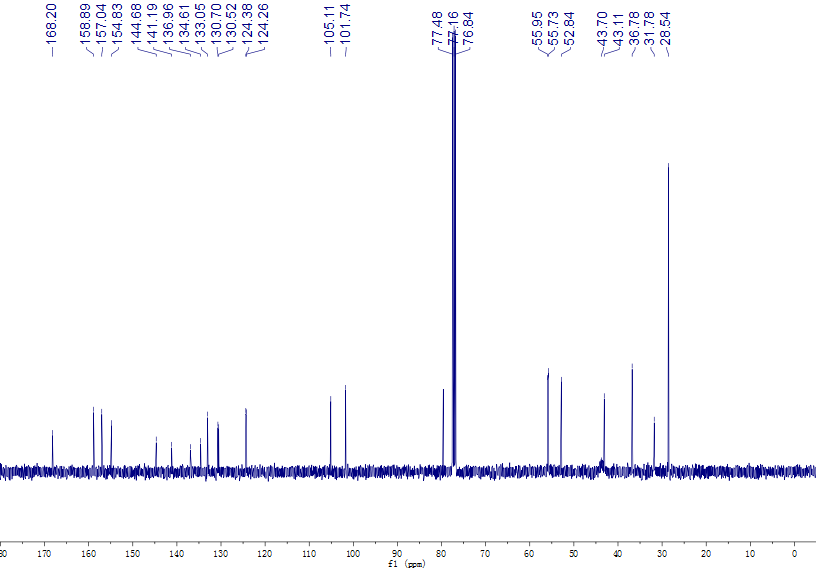
**

^13^C NMR, CDCl_3_

101M, 298K

^13^C-NMR of **7a.**

**
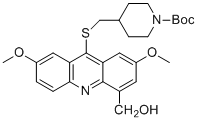
7b
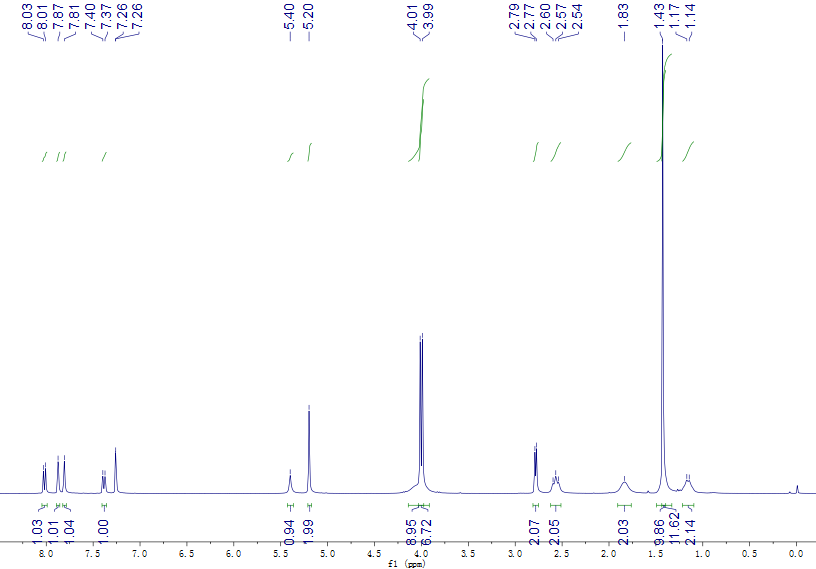
**

^1^H NMR, CDCl_3_

400M, 298K

^1^H-NMR of **7b.**

**
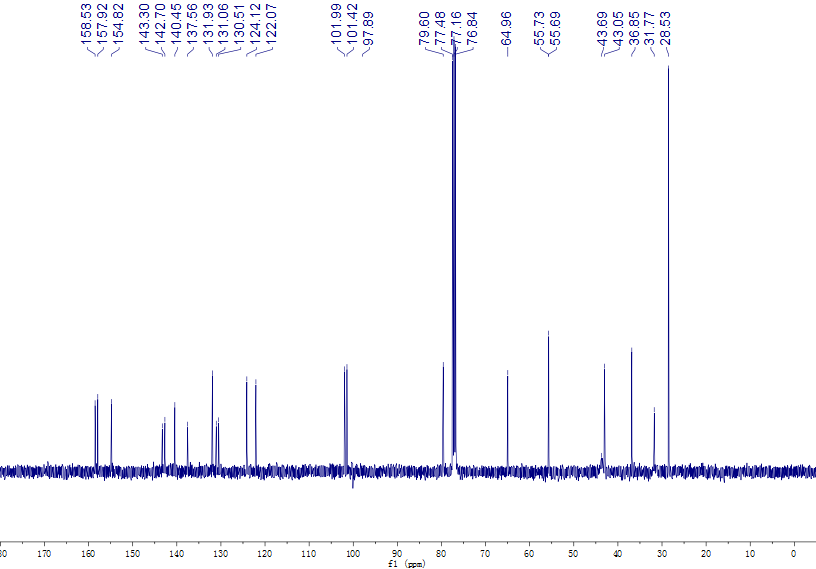

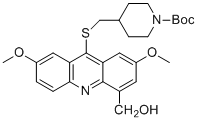
**

^13^C NMR, CDCl_3_

101M, 298K

^13^C-NMR of **7b.**


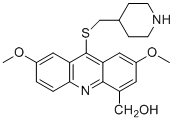
**7
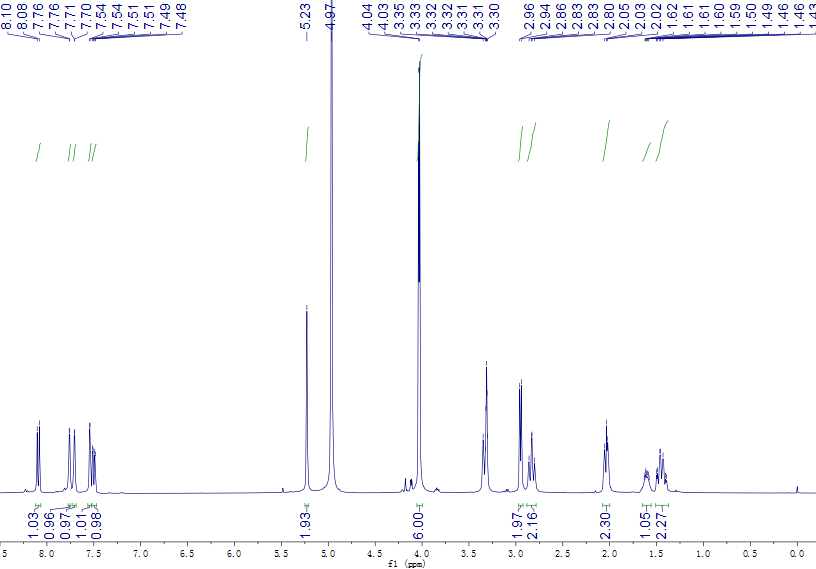
**

^1^H NMR, MeOD

400M, 298K

H_2_O

^1^H-NMR of **7.**

**
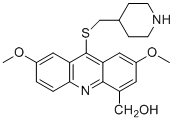

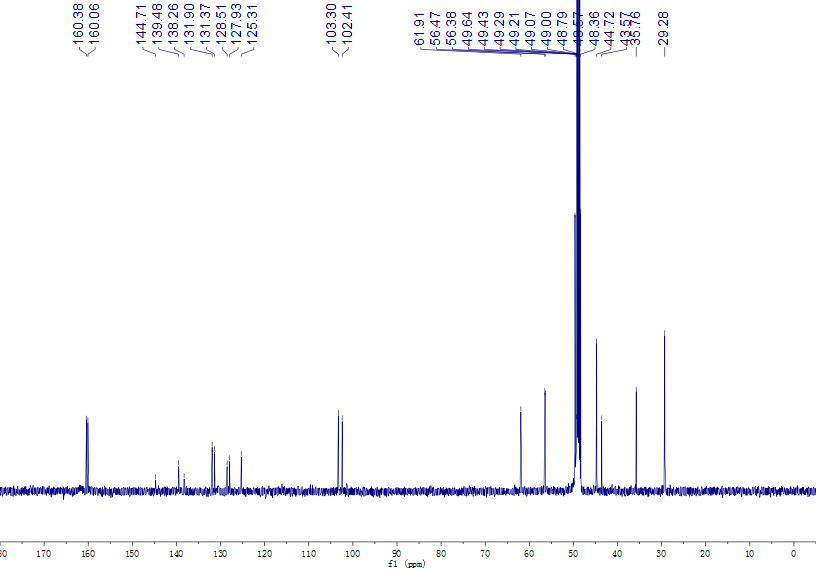
**

^13^C NMR, MeOD

101M, 298K

^13^C-NMR of **7.**

**8**

**
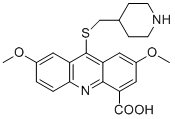

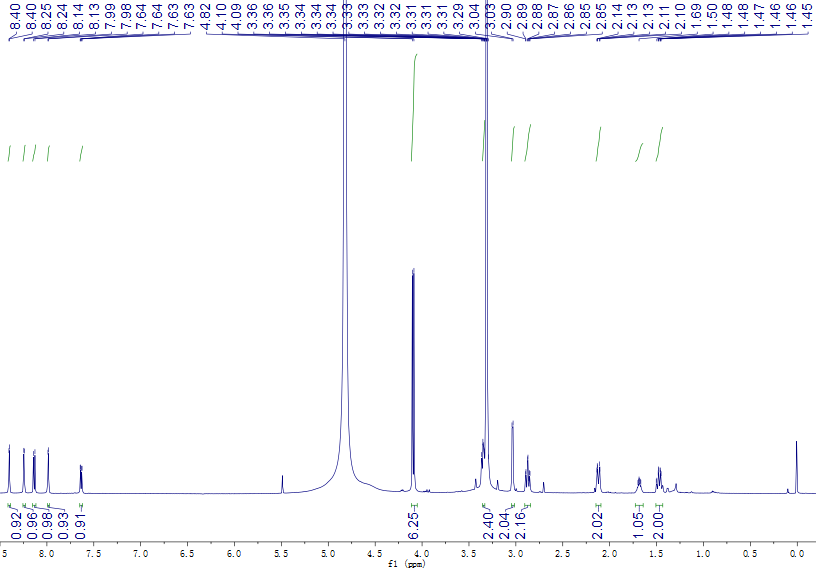
**

^1^H NMR, MeOD

400M, 298K

H_2_O

^1^H-NMR of **8.**

**
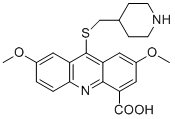

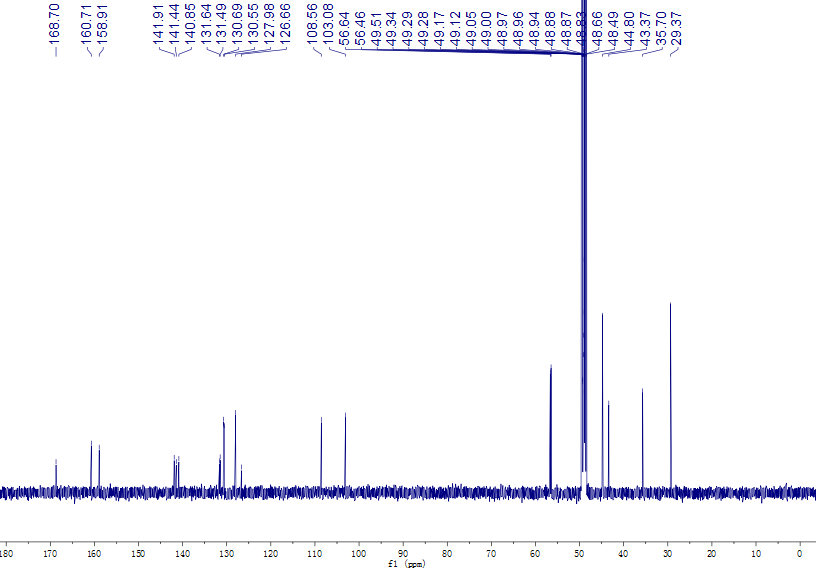
**

^13^C NMR, MeOD

126M, 298K

^13^C-NMR of **8.**

**9a**

**
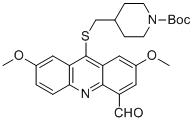

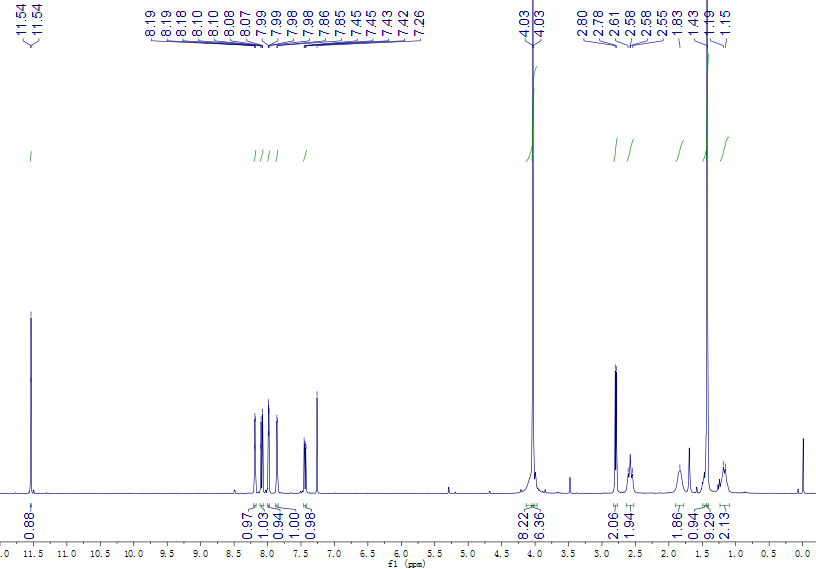
**

^1^H NMR, CDCl_3_

400M, 298K

^1^H-NMR of **9a.**

**
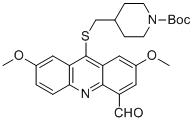

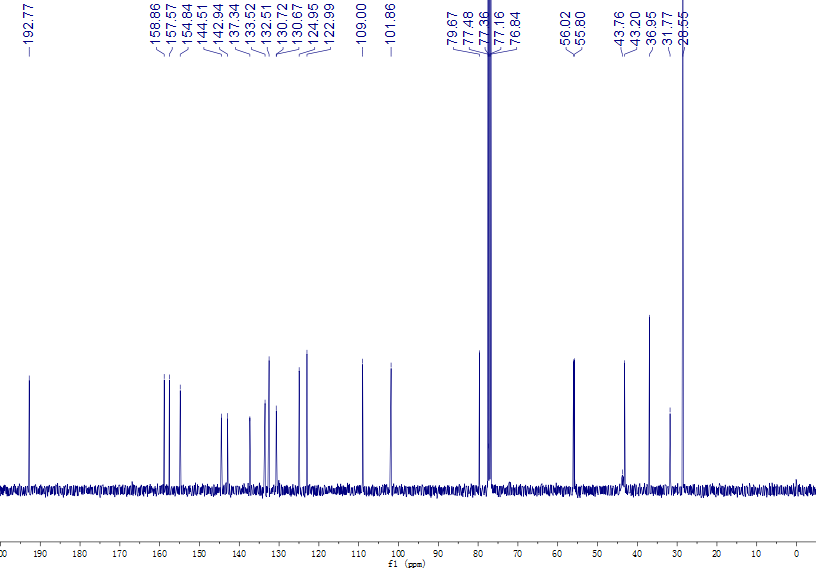
**

^13^C NMR, CDCl_3_

101M, 298K

^13^C-NMR of **9a.**

**9b**

**
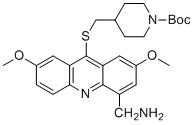

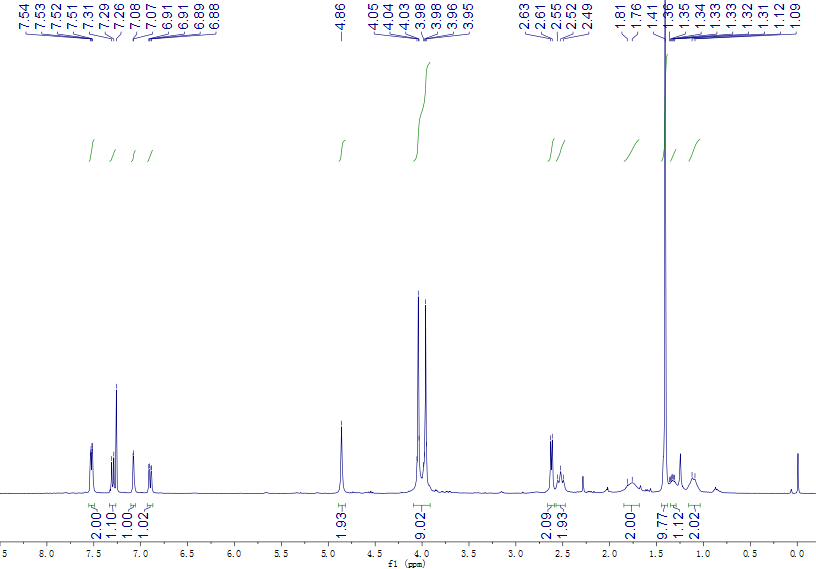
**

^1^H NMR, CDCl_3_

400M, 298K

^1^H-NMR of **9b.**

**
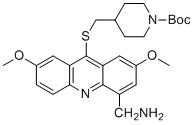

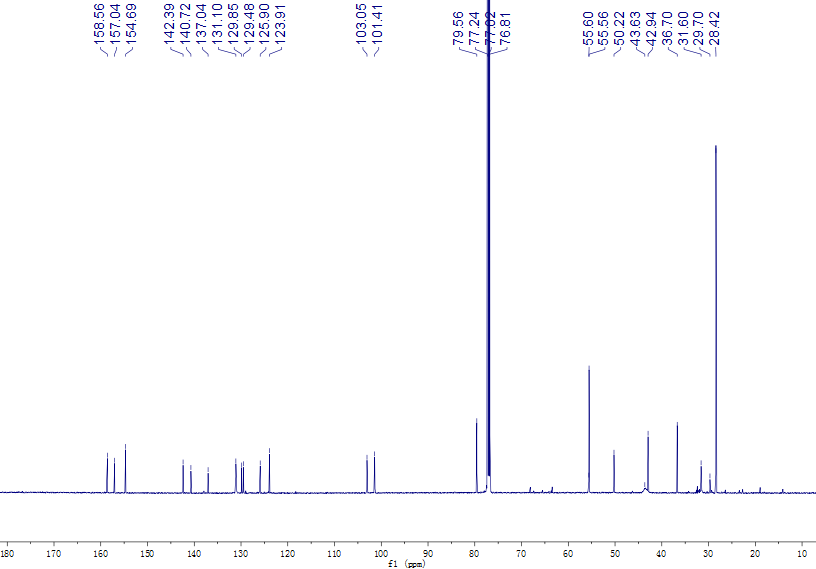
**

^13^C NMR, CDCl_3_

151M, 298K

^13^C-NMR of **9b.**

**9**

**
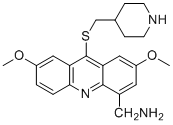

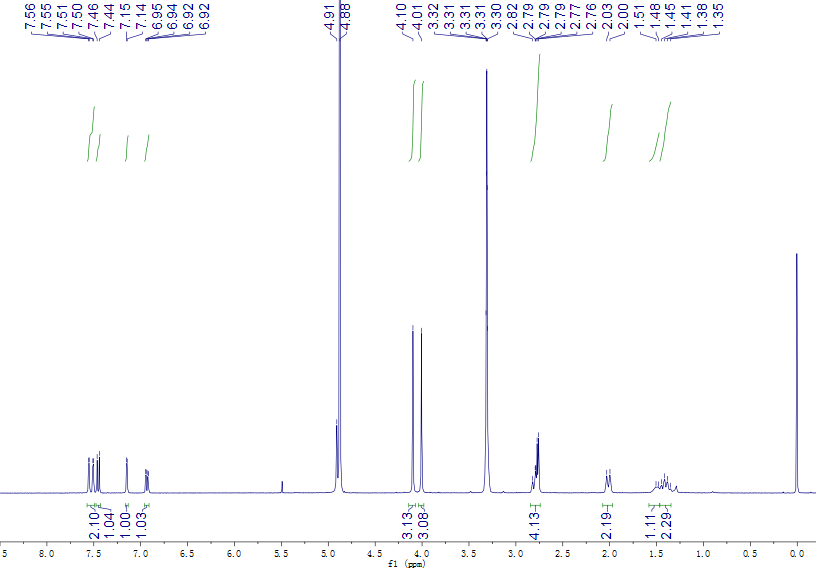
**

^1^H NMR, MeOD

400M, 298K

H_2_O

^1^H-NMR of **9.**

**
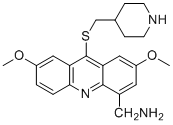

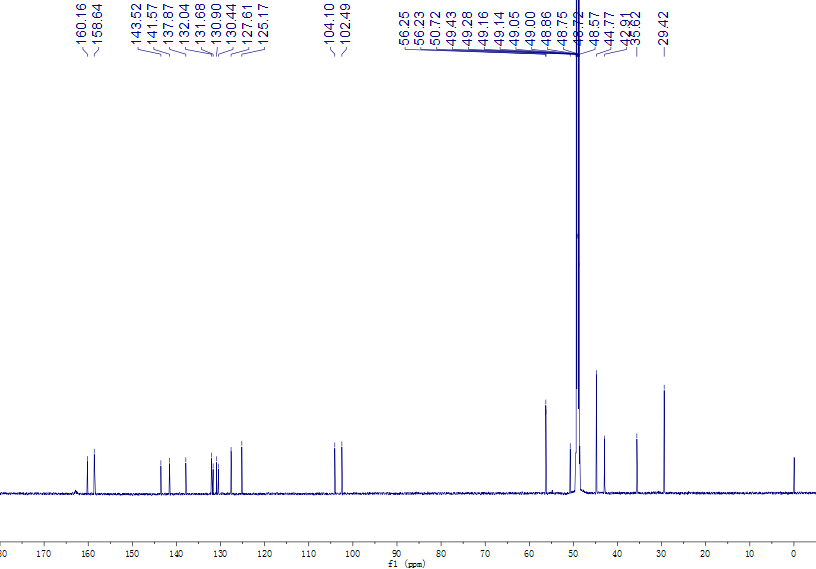
**

^13^C NMR, MeOD

151M, 298K

^13^C-NMR of **9.**

**10a**


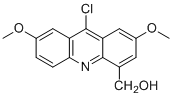
**
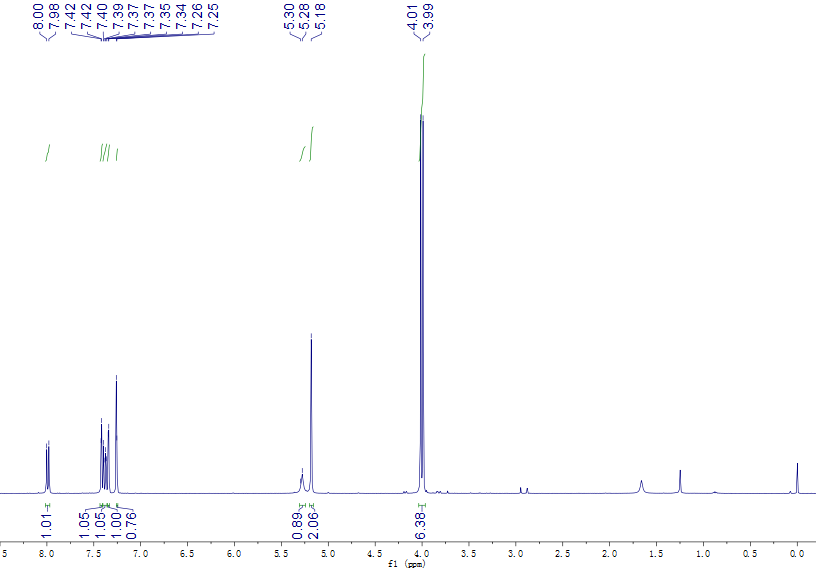
**

^1^H NMR, CDCl_3_

400M, 298K

^1^H-NMR of **10a.**

**
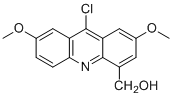

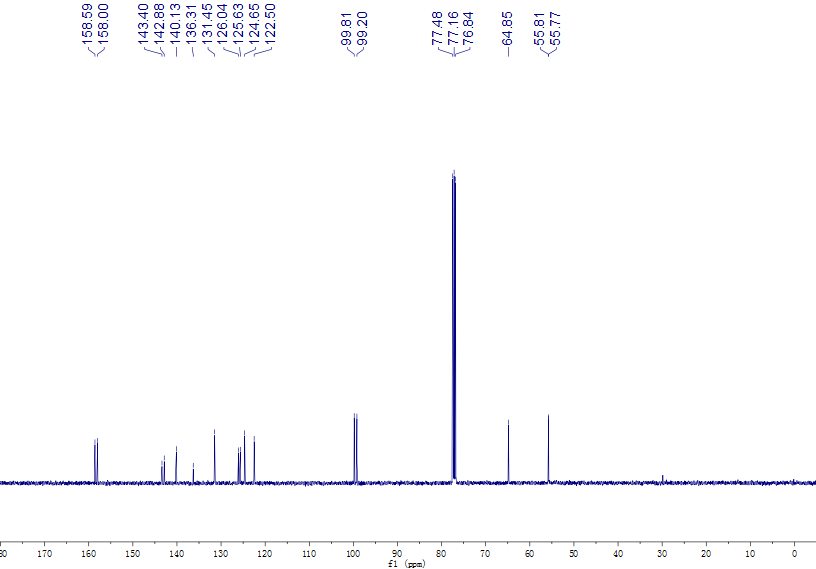
**

^13^C NMR, CDCl_3_

101M, 298K

^13^C-NMR of **10a.**

**10b**

**
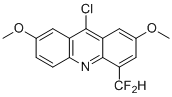

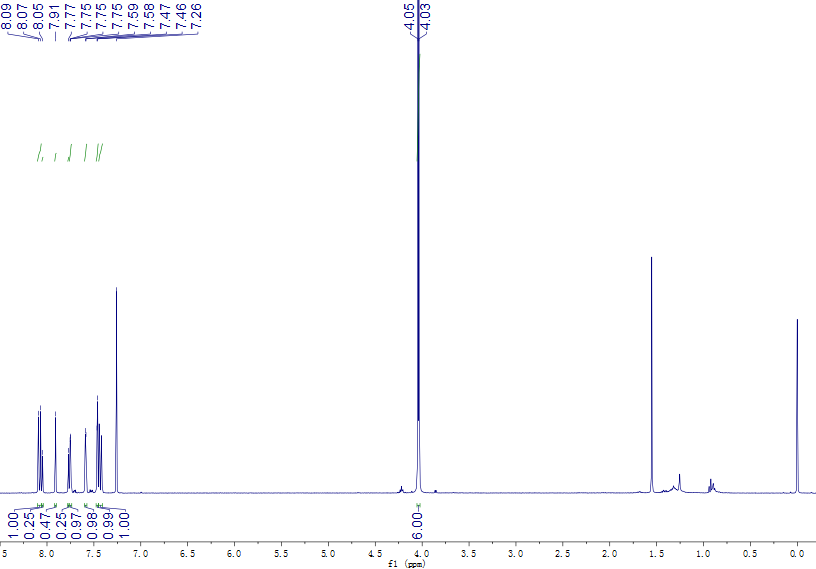
**

^1^H NMR, CDCl_3_

400M, 298K

^1^H-NMR of **10b.**

**
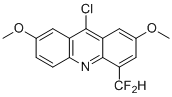

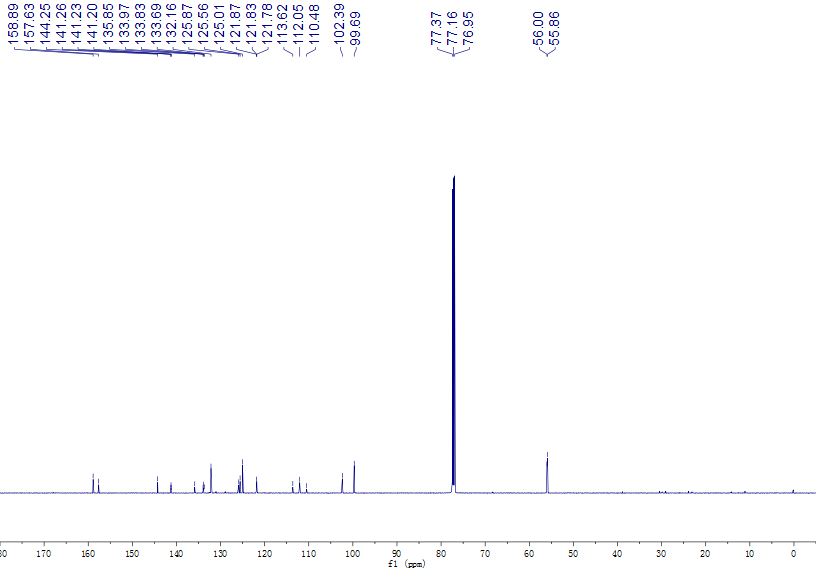
**

^13^C NMR, CDCl_3_

151M, 298K

^13^C-NMR of **10b.**

**10**

**
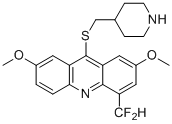

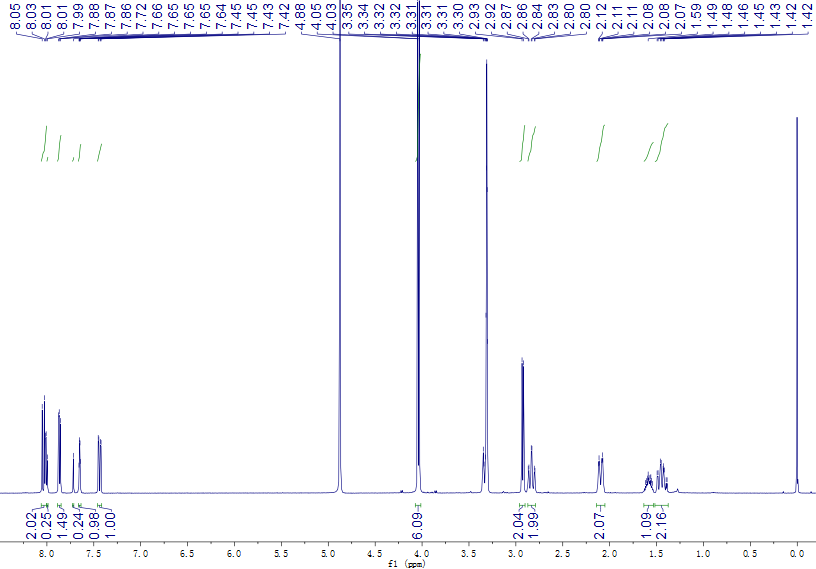
**

^1^H NMR, MeOD

400M, 298K

H_2_O

^1^H-NMR of **10.**

**
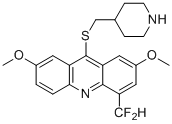

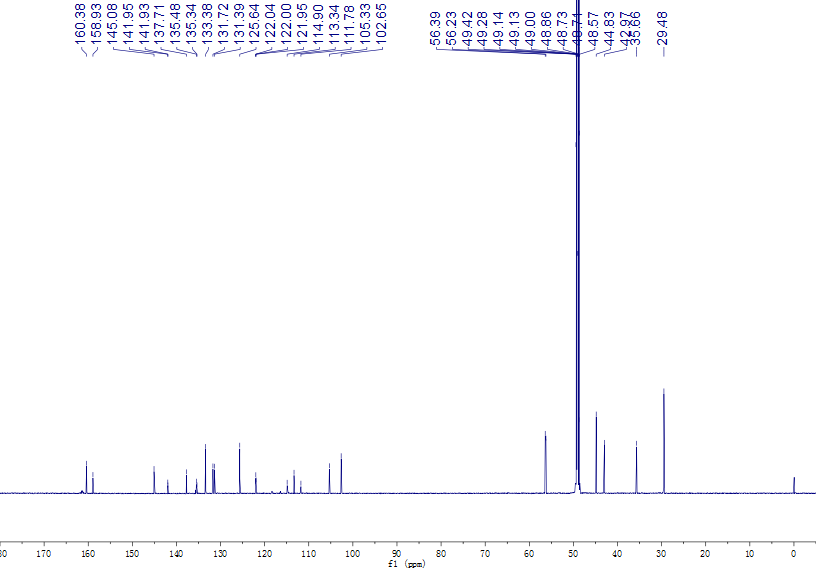
**

^13^C NMR, MeOD

151M, 298K

^13^C-NMR of **10.**

**11a**


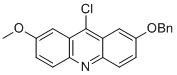
**
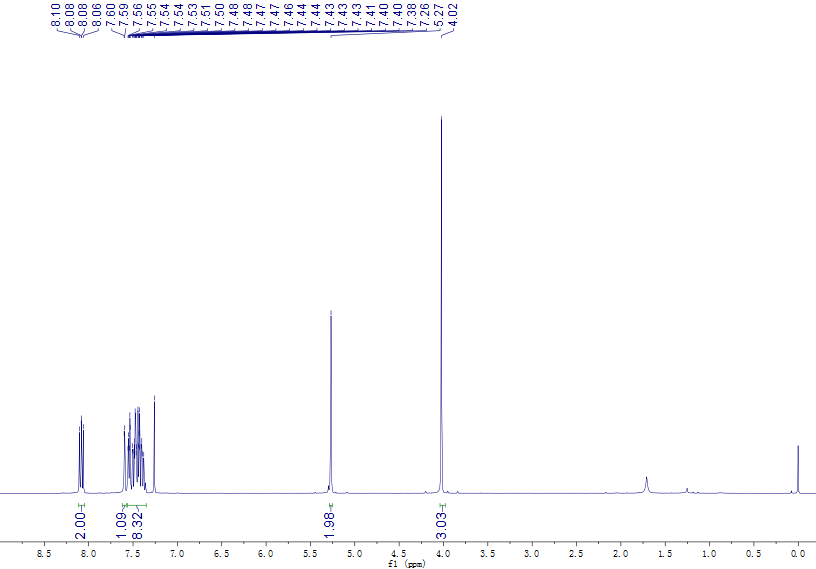
**

^1^H NMR, CDCl_3_

400M, 298K

^1^H-NMR of **11a.**

**
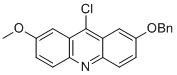

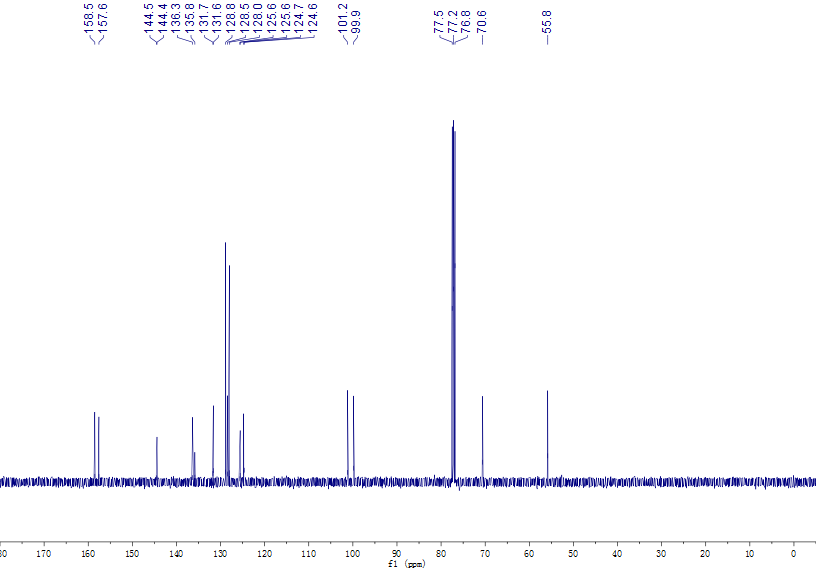
**

^13^C NMR, CDCl_3_

100M, 298K

^13^C-NMR of **11a.**

**11**


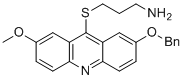
**
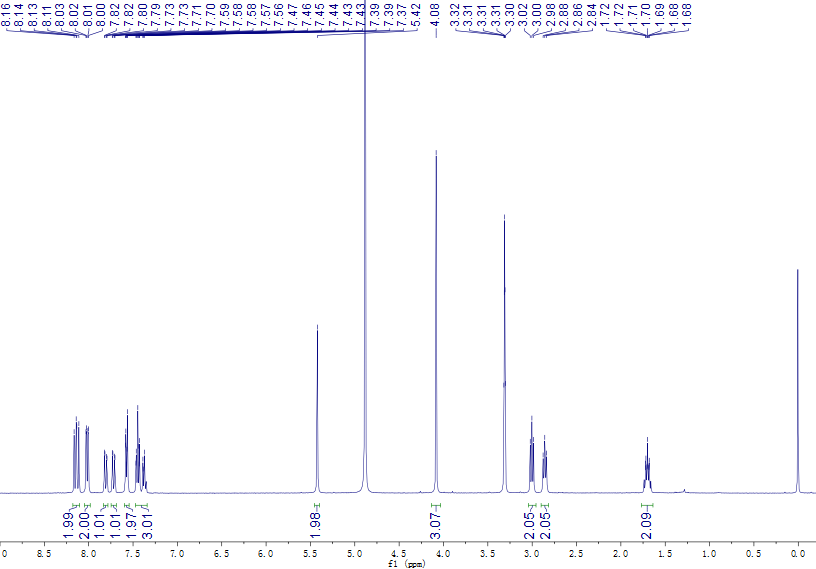
**

^1^H NMR, MeOD

400M, 298K

^1^H-NMR of **11.**

H_2_O

**
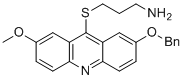

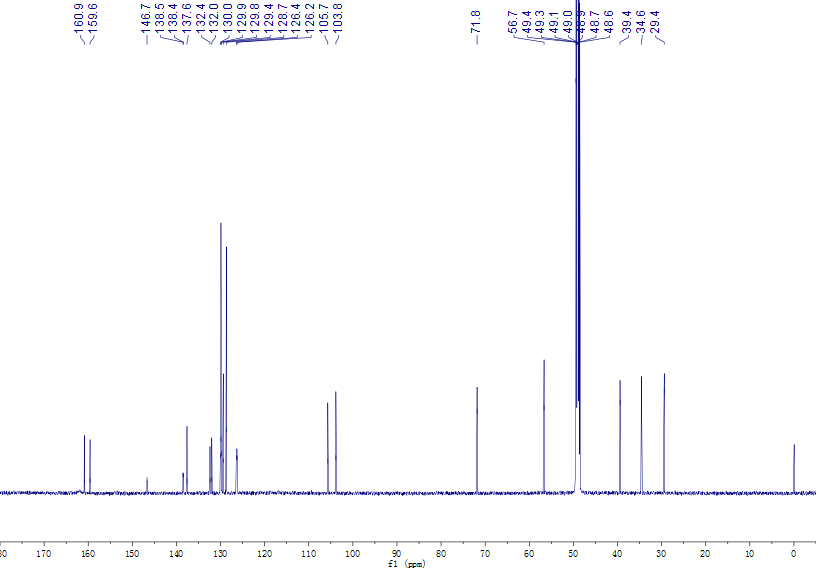
**

^13^C NMR, MeOD

151M, 298K

^13^C-NMR of **11.**

**12a**

**
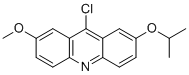

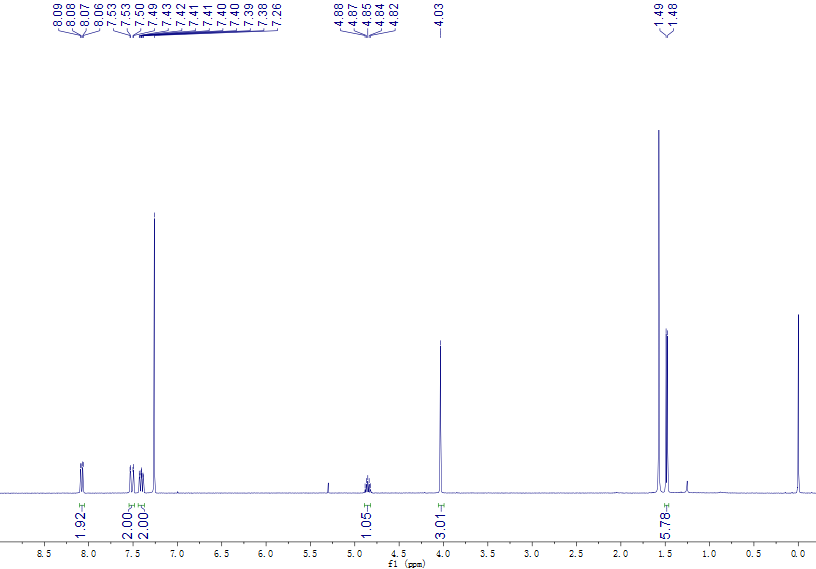
**

^1^H NMR, CDCl_3_

400M, 298K

^1^H-NMR of **12a.**

**
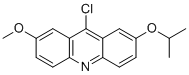

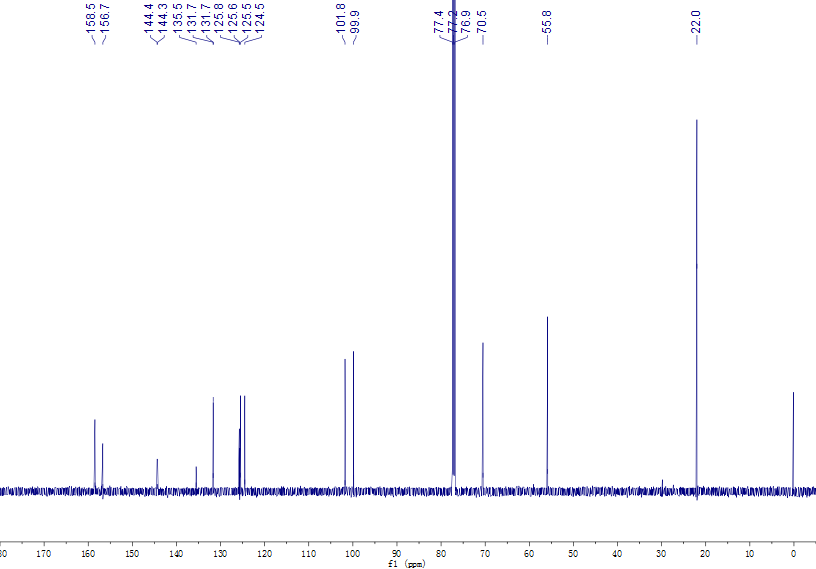
**

^13^C NMR, CDCl_3_

101M, 298K

^13^C-NMR of **12a.**

**12**


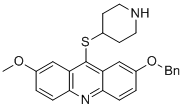
**
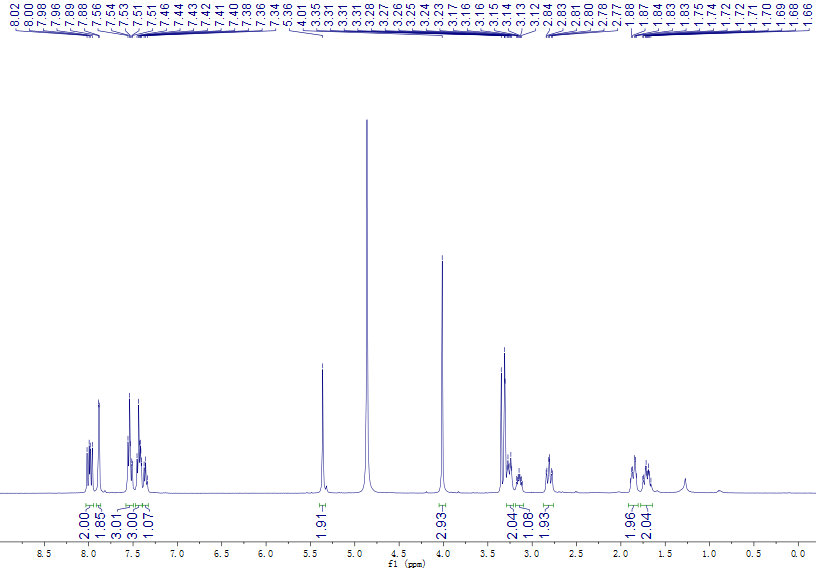
**

^1^H NMR, MeOD

400M, 298K

H_2_O

^1^H-NMR of **12.**

**
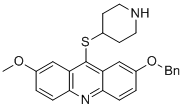

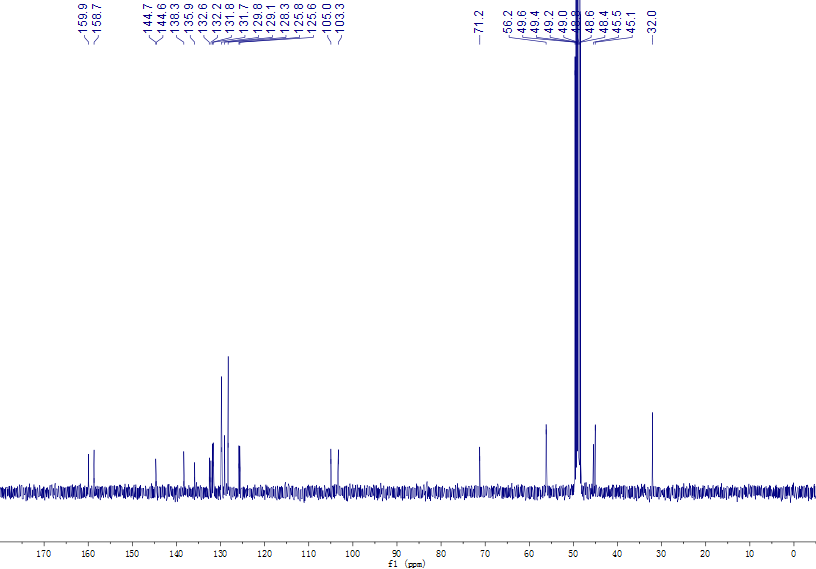
**

^13^C NMR, MeOD

101M, 298K

^13^C-NMR of **12.**


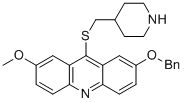
**13
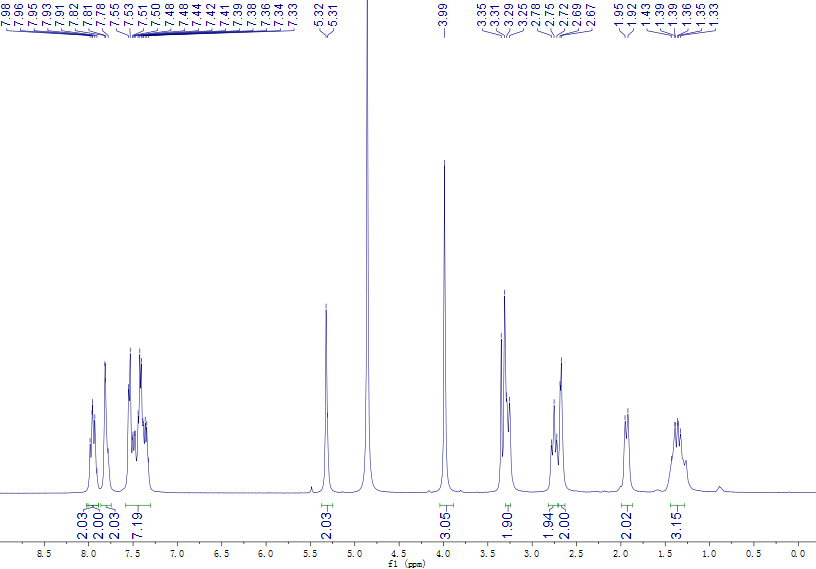
**

H_2_O

^1^H NMR, MeOD

400M, 298K

^1^H-NMR of **13.**

**
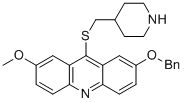

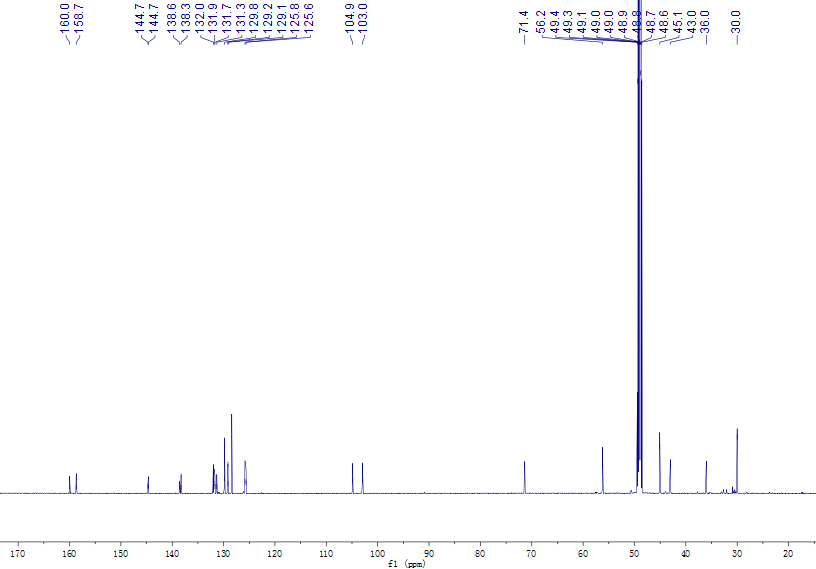
**

^13^C NMR, MeOD

126M, 298K

^13^C-NMR of **13.**

**14**

^1^H NMR, MeOD

400M, 298K

H_2_O

^1^H-NMR of **14.**

^13^C NMR, MeOD

151M, 298K

^13^C-NMR of **14.**

**15a**

^1^H NMR, CDCl_3_

400M, 298K

^1^H-NMR of **15a.**

^13^C NMR, CDCl_3_

101M, 298K

^13^C-NMR of **15a.**

**15**

^1^H-NMR of **15.**

^1^H NMR, MeOD

400M, 298K

H_2_O

^13^C NMR, MeOD

101M, 298K

^13^C-NMR of **15.**

**16a**

^1^H NMR, CDCl_3_

400M, 298K

^1^H-NMR of **16a.**

^13^C NMR, CDCl_3_

101M, 298K

^13^C-NMR of **16a.**

**16b**

^1^H NMR, CDCl_3_

400M, 298K

^1^H-NMR of **16b.**

^13^C NMR, CDCl_3_

101M, 298K

^13^C-NMR of **16b.**

**16**

^1^H NMR, MeOD

400M, 298K

H_2_O

^1^H-NMR of **16.**

^13^C NMR, MeOD

126M, 298K

^13^C-NMR of **16.**

**17a**

^1^H NMR, CDCl_3_

400M, 298K

^1^H-NMR of **17a.**

^13^C NMR, CDCl_3_

101M, 298K

^13^C-NMR of **17a.**

**17b**

^1^H NMR, CDCl_3_

400M, 298K

^1^H-NMR of **17b.**

^13^C NMR, CDCl_3_

126M, 298K

^13^C-NMR of **17b.**

**17**

H_2_O

^1^H NMR, MeOD

400M, 298K

^1^H-NMR of **17.**

^13^C NMR, MeOD

126M, 298K

^13^C-NMR of **17.**

**18a**

^1^H NMR, CDCl_3_

400M, 298K

^1^H-NMR of **18a.**

^13^C NMR, CDCl_3_

126M, 298K

^13^C-NMR of **18a.**

**18b**

^1^H NMR, CDCl_3_

400M, 298K

^1^H-NMR of **18b.**

^13^C NMR, CDCl_3_

126M, 298K

^13^C-NMR of **18b.**

**18**

^1^H NMR, MeOD

400M, 298K

H_2_O

^1^H-NMR of **18.**

^13^C NMR, MeOD

126M, 298K

^13^C-NMR of **18.**

**19a**

^1^H NMR, CDCl_3_

400M, 298K

^1^H-NMR of **19a.**

^13^C NMR, CDCl_3_

126M, 298K

^13^C-NMR of **19a.**

**19b**

^1^H NMR, CDCl_3_

400M, 298K

^1^H-NMR of **19b.**

^13^C NMR, CDCl_3_

126M, 298K

^13^C-NMR of **19b.**

**19**

^1^H NMR, MeOD

400M, 298K

H_2_O

^1^H-NMR of **19.**

^13^C NMR, MeOD

101M, 298K

^13^C-NMR of **19.**

**20a**

^1^H NMR, CDCl_3_

400M, 298K

^1^H-NMR of **20a.**

^13^C NMR, CDCl_3_

101M, 298K

^13^C-NMR of **20a.**

**20**

^1^H NMR, MeOD

400M, 298K

H_2_O

^1^H-NMR of **20.**

^13^C NMR, MeOD

151M, 298K

^13^C-NMR of **20.**

**21a**

^1^H NMR, CDCl_3_

400M, 298K

^1^H-NMR of **21a.**

^13^C NMR, CDCl_3_

101M, 298K

^13^C-NMR of **21a.**

**21b**

^1^H NMR, CDCl_3_

400M, 298K

^1^H-NMR of **21b.**

^13^C NMR, CDCl_3_

101M, 298K

^13^C-NMR of **21b.**

**21c**

^1^H NMR, CDCl_3_

400M, 298K

^1^H-NMR of **21c.**

^13^C NMR, CDCl_3_

101M, 298K

^13^C-NMR of **21c.**

**21**

^1^H NMR, MeOD

400M, 298K

H_2_O

^1^H-NMR of **21.**

^13^C NMR, MeOD

151M, 298K

^13^C-NMR of **21.**

**22a**

^1^H NMR, CDCl_3_

400M, 298K

^1^H-NMR of **22a.**

^13^C NMR, CDCl_3_

101M, 298K

^13^C-NMR of **22a.**

**22b**

^1^H NMR, CDCl_3_

400M, 298K

^1^H-NMR of **22b.**

^13^C NMR, CDCl_3_

101M, 298K

^13^C-NMR of **22b.**

**22c**

^1^H NMR, CDCl_3_

400M, 298K

^1^H-NMR of **22c.**

^13^C NMR, CDCl_3_

151M, 298K

^13^C-NMR of **22c.**

**22**

^1^H NMR, MeOD

400M, 298K

H_2_O

^1^H-NMR of **22.**

^13^C NMR, MeOD

151M, 298K

^13^C-NMR of **22.**
